# Efficient Hydro‐ and Organogelation by Minimalistic Diketopiperazines Containing a Highly Insoluble Aggregation‐Induced, Blue‐Shifted Emission Luminophore**

**DOI:** 10.1002/chem.202102861

**Published:** 2021-10-22

**Authors:** Martin Molkenthin, Werner M. Nau, Boris J. Nachtsheim

**Affiliations:** ^1^ Institut für Organische und Analytische Chemie Universität Bremen Leobener Straße 7 28359 Bremen Germany; ^2^ Department of Life Sciences and Chemistry Jacobs University Bremen Campus Ring 1 28759 Bremen Germany

**Keywords:** fluorescence, gels, peptides, sol-gel-process, supramolecular chemistry

## Abstract

We report the synthesis, gelation abilities and aggregation‐induced, blue‐shifted emission (AIBSE) properties of two minimalistic diketopiperazine‐based gelators. Despite containing a highly insoluble luminophore that makes up more than half of their respective molecular masses, efficient hydrogelation by multiple stimuli for one and efficient organogelation for the other compound are reported. Insights into the aggregation and gelation properties were gained through examination of the photophysical and material properties of selected gels, which are representative of the different modes of gelation. The synthesis of the gelators is highly modular and based on readily available amino acid building blocks, allowing the efficient and rapid diversification of these core structures and fine‐tuning of gel properties.

## Graphical abstract

**Minimalistic and highly efficient hydro‐ and organogelators** were obtained by the combination of a highly insoluble luminophore capable of aggregation‐induced blue shifted emission (AIBSE) with natural amino‐acid derived diketopiperazines. The gelators were investigated with regard to their aggregation and gelation behavior, and their photophysical and material properties. The modular approach demonstrates a potent strategy to turn an otherwise poorly soluble polycyclic aromatic compound into hydro‐ and organogelators.

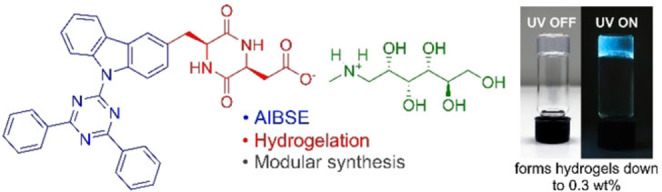

## Introduction

Compounds and materials that show aggregation‐induced emission (AIE) have been of ever growing interest in the last two decades.^[1]^ Self‐assembling, supramolecular materials that show AIE are especially interesting due to their wide array of functionalities and possible applications.^[2]^

One of the most diverse supramolecular materials are gels, especially at their interface with AIE, as gelators that contain AIE luminophores can exemplify the gelation process and lead to a better understanding of it.^[3]^ Closely related to the AIE phenomenon is the so‐called aggregation‐induced blue‐shifted emission (AIBSE) phenomenon.^[4]^ There have only been a handful of molecules described so far, which are capable of AIBSE as well as hydro‐ or organogelation.^[5]^ Over the last decades, a plethora of different small molecules have been shown to form hydrogels.^[6]^ However, the development of new hydrogelators and the understanding of their gelation processes remain a challenge.^[7]^ One of the most studied classes of low‐molecular weight gelators (LMWGs) are peptide‐based compounds.[^7b^, ^8^] Some (hydro‐)gelators based on peptides containing AIEgens (aggregation‐induced emission luminogens) have been described before.[^3e^, ^9^] Their structures are often complex and/or their gelation abilities dependent on the luminophore. 2,5‐Diketopiperazines (DKPs) have been shown to be minimalistic yet powerful hydro‐ and organogelators, owing to their ability to self‐assemble through strong hydrogen bonding.^[10]^ Moreover, they can be conveniently synthesized from easily available, natural amino acids in high yields and within few synthetic steps, which make them highly interesting compounds for the development of new hydrogelators.^[11]^ So far, there is only one known example for a DKP‐bound AIEgen, which however has not been reported to form gels.^[12]^

**Scheme 1:**
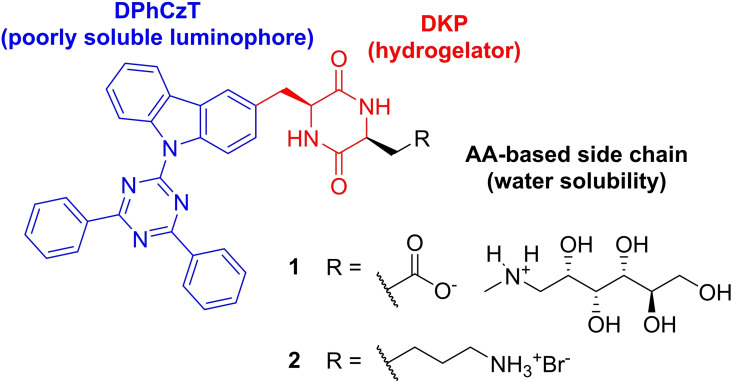
Concept for a minimalistic DKP and **DPhCzT**‐based hydrogelator and structures of compounds studied for their gelation abilities (**1**, **2**).

Herein, we report the synthesis of two minimalistic DKPs capable of efficient organo‐ and hydrogelation that exhibit AIBSE, and describe their aggregation behavior and their luminescent properties. They contain the heterocyclic compound 9‐(4,6‐diphenyl‐1,3,5‐triazin‐2‐yl)carbazole (**DPhCzT**, Scheme 1, in blue), which makes up more than 50 % of their respective molecular masses. Due to its poor solubility in most solvents it is a challenging luminophore on the one hand, but on the other hand is appealing due to its intriguing photophysical properties, its ease of synthesis and possible modifications.^[13]^ The investigated target structures **1** and **2** are shown in Scheme 1.

The lysine side chain of **2** and the aspartic acid side chain of **1** were chosen to maximize the solubility of the gelators. Lysine, aspartic acid and glutamic acid derived side chains with free amino or carboxylic acid groups are a common structural motif in DKPs which can modulate gelation abilities through protonation or deprotonation and thereby maximize the hydrophilicity and solubility of the gelators.[^10a^, ^14^]. The *N*‐methyl‐d‐glucamine (meglumine) containing salt **1** was prepared since meglumine has been shown to drastically improve the solubility of an otherwise hydrophobic carboxylic acid salt in water compared to its sodium salt.^[15]^

## Results and Discussion

### Synthesis

**Scheme 2:**
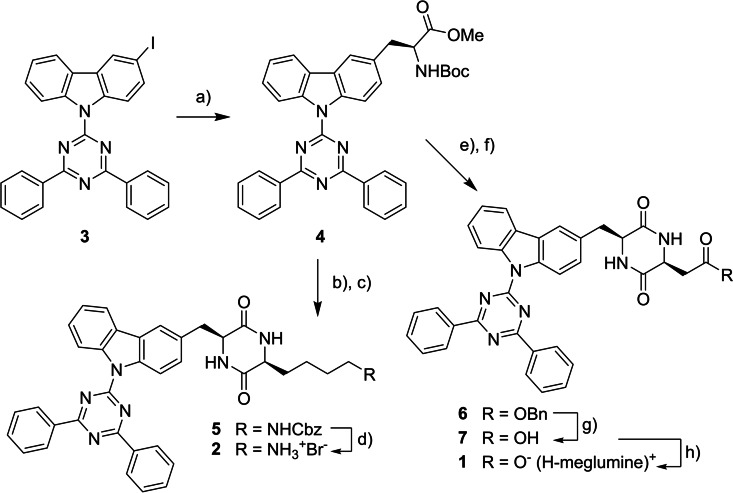
Synthesis of diketopiperazines **1** and **2**. Conditions: a) Pd_2_dba_3_, SPhos, (*R*)‐BocNHCH(CH_2_ZnI)CO_2_Me, DMF/THF, 30 °C, 51 %. b) i. TFA, DCM, rt, 18 h; ii. NEt_3_, Boc‐Lys(*Z*)‐OH, DCM/DMF, HBTU, 0 °C ‐ rt, 2.5 d, 90 %. c) i. TFA, DCM, rt, 18 h; ii. NEt_3_, AcOH, MeCN, *iso*‐BuOH, 100 °C, 3 h, 90 %. d) HBr/AcOH, TFA, rt, 75 min, 86 %. e) i. TFA, DCM, rt, 18 h; ii. NEt_3_, Boc‐Asp(OBn)‐OH, DCM/DMF, HBTU, 0 °C‐rt, 2.5 d, 87 %. f) i. TFA, DCM, rt, 18 h; ii. NEt_3_, AcOH, MeCN, *iso*‐BuOH, 100 °C, 4 h, 81 %. g) BBr_3_, DCM, −15 °C‐rt, 20 h, 84 %. h) *N*‐methyl‐d‐glucamine, EtOH, 50 °C, 3 h, 85 %. See Scheme 1 for the structure of (H‐meglumine)^+^. For detailed synthetic procedures and the full synthesis plan, see the Supporting Information.

Gelators **1** and **2** were synthesized from the protected amino acid **4**. The synthesis of the key intermediate **4** was accomplished by a palladium‐catalyzed negishi cross‐coupling reaction of the iodinated heterocycle **3** with an iodoalanine derivative in 51 % yield (Scheme 2).^[16]^

Building block **4** was then converted into lysine‐ and aspartic acid‐derived, protected diketopiperazines **5** and **6** in high yields via Boc‐deprotection, amide coupling, second Boc‐deprotection and acid‐catalyzed cyclization (**5**: 81 % yield over four steps, **6**: 70 % yield over four steps (see the Supporting Information for a full scheme).[^11a^, ^17^] The deprotection of **5** proceeded smoothly in 86 % yield using hydrobromic acid in acetic acid.^[18]^ The deprotection of **6** proved to be difficult at first (see chapter 11 of the Supporting Information). However, we were delighted to find that the deprotection proceeded in high yield of 84 % using an excess of boron tribromide in dry dichloromethane.^[19]^ Finally, the free acid **7** was converted to its meglumine salt **1** in 85 % yield by simple heating in ethanol.^[15]^ Notably, all steps proceeding from **4** were performed without the use of column chromatography, which underlines the robustness of the synthesis of the chosen target structures.

### General luminescence properties of the synthesized DPhCzT derivatives

Compound **4** and all derivatives showed blue fluorescence in the solid state (Figure S2). Compounds **1**, **2** and **7** showed almost the same fluorescence emission spectra in the solid state, with quantum yields ranging between 3.0 %–3.9 % (Table S7). **DPhCzT** has been described to show ultra‐long phosphorescence at room temperature in the solid state,^[13b]^ but none was found for **3** or any of its derivatives. In a recent publication by chen et al., the long‐lived phosphorescence of **DPhCzT** was attributed to an isomer present as a trace impurity, in which the carbazole unit is replaced by a benzo[*f*]indole unit. The compound stemmed from a 1*H*‐benzo[*f*]indole impurity present in commercial carbazole, which reacted in the same way as carbazole, forming the isomer.^[20]^ In this synthesis, the commercial carbazole was iodinated and the product of the reaction recrystallized prior to the synthesis of **3**, which has most likely removed the impurity and would explain why no phosphorescence was found for the presently prepared samples of **3** or any of its derivatives.

**Figure 1:**
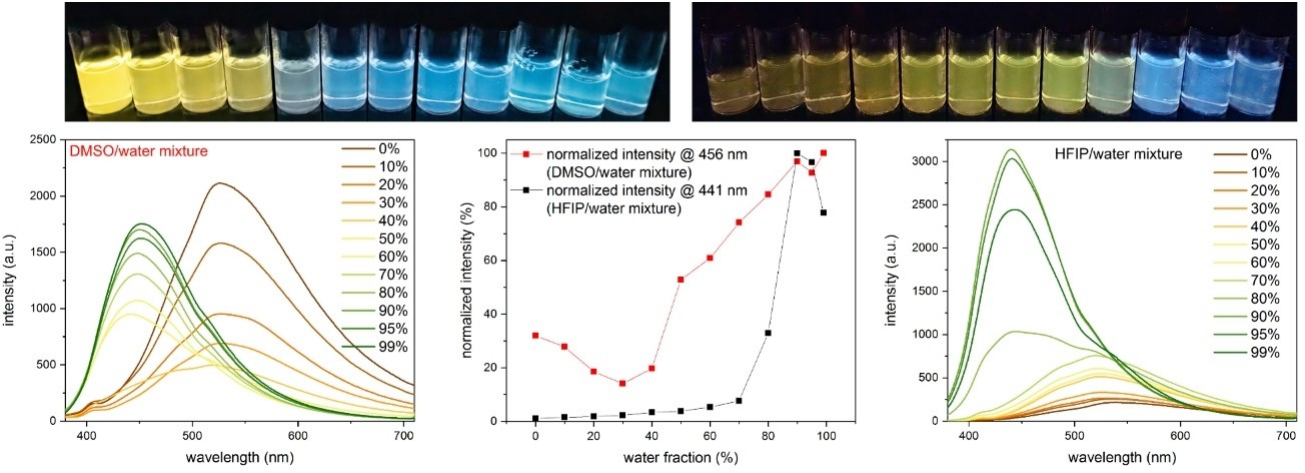
AIBSE‐behavior of **2** in DMSO and HFIP with increasing water content (from left to right). (Left) fluorescence emission spectra of DMSO/water mixtures (water content in %). (Middle) comparison of normalized fluorescence emission intensities at the respective maximum caused by aggregation. (Right) fluorescence emission spectra in HFIP/water mixtures (water content in %). Excitation at 365 nm and c=100 μM in all cases.

Diketopiperazines **1** and **2** showed AIBSE properties in DMSO/water mixtures (Figures 1 and S4, S7). With increasing water content, fluorescence emission changed from a yellow/slightly greenish color to a blue color (Figures S3, S5 and S6, S8). In hexafluoroisopropanol (HFIP)/water mixtures, both compounds showed AIBSE behavior with increasing water content as well (Figures 1 and S9–10, S12–13). In contrast to the DMSO/water mixture, there was a strong increase of the emission intensity with increasing water content, which is explained by the exceptionally low fluorescence quantum yields of **1** and **2** in HFIP (*Φ*≤0.001) compared to other organic solvents (Table S2, S3). In contrast to this, the quantum yields in 99 % water and 1 % HFIP mixtures are much higher (*Φ*=0.026 for **1** and *Φ*=0.020 for **2**, Table S1).

### Gelation experiments

After compound **2** was synthesized, its gelation abilities were investigated. **2** was not completely soluble at a concentration of 10 mg/mL in any common organic solvent, not even upon heating. Upon the addition of small amounts of trifluoroacetic acid, **2** readily dissolved in DCM, CHCl_3_ and HFIP, resulting in a bright yellow solution that showed increased UV light absorption (Figure S27). We also found the same reaction towards TFA with **3** in DCM, as well as any derivative of it, except for **1**. We hypothesize that this as a solubilizing, de‐aggregating hydrogen bonding interaction between TFA and the heterocyclic nitrogens. The addition of a co‐solvent to the HFIP/TFA solution of **2** finally induced gelation accompanied by a decolorization (Table 1, Figure S42). Translucent gels were formed with water, DMF and DMSO as co‐solvents. Opaque gels formed with methanol, isopropyl alcohol, tetrahydrofuran, 1,4‐dioxane and ethyl acetate as co‐solvents. Notably, mixtures with acetonitrile, acetone and ethyl acetate only formed gels after prolonged standing while simultaneously becoming more translucent. Gels with DMF and DMSO as co‐solvents became transparent after three days (Figure S43). **2** formed gels with all tested co‐solvents except for toluene and dichloromethane. When methanol was added to both of these solutions, gelation occurred within 24 h. In all cases, the gelation was not reversible by heating.

**Table 1:** Gelation properties of **2** in HFIP/co‐solvent mixtures^.[a]^

Co‐solvent	**2** ^[b]^	Co‐solvent	**2** ^[b]^
water	TG	dimethyl sulfoxide	TG/G^[c]^
methanol	OG	tetrahydrofuran	OG
isopropyl alcohol	OG	1,4‐dioxane	OG
acetonitrile	Sol/TG^[c]^	ethyl acetate	Sol/OG^[c]^
acetone	Sol/TG^[c]^	toluene	S/OG^[d]^
dimethyl formamide	TG/G^[c]^	dichloromethane	S/OG^[d]^

[a] A co‐solvent (500 μL) was added to a solution of **2** (10 mg) and TFA (15 μL) in HFIP (500 μL). [b] 1 h after addition of the co‐solvent. G: transparent gel; OG: opaque gel; S: solution; TG: translucent gel; [c] after 3 d. [d] 24 h after addition of MeOH (150 μL).

The critical gelator concentration (CGC) for the HFIP/water (1 : 1) gel was determined to be 0.4 wt% (7.4 mM) and gels showed blue fluorescence (Figure S60).

**Figure 2:**
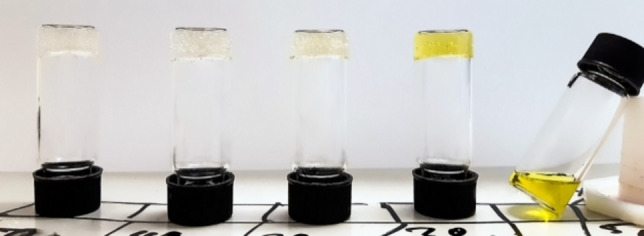
Gelation behavior of **2** in HFIP/water/TFA mixtures at 0.5 wt% with different water fractions. From left to right: (50, 40, 30, 20, 10) vol% H_2_O. See Table S6 for more information.

Further experiments were performed to find the smallest fraction of water that causes a HFIP/TFA/water mixture of **2** to form a stable gel (Figure 2, Table S6). At 0.5 wt% of **2**, we found that a water fraction of 10 % did not cause gelation, while a water fraction of 20 % or higher caused gelation. The gel obtained from a water fraction of 20 % had an intense yellow color that did not fade over time, showing the presence of TFA‐N‐heterocycle interactions within the gel.

At higher water fractions, the gels were almost colorless (30 % and 40 % water) or colorless (50 % water). (Protic) co‐solvents could influence TFA interactions with the gelator in a twofold way: On the one hand, solubilizing hydrogen bonding between TFA and the heterocycle might be broken up, leading to aggregation through aggregation of the heterocycles. On the other hand, co‐solvents might break up solubilizing hydrogen bonding between TFA, HFIP and the DKP residue since HFIP has been shown to be significantly potent at solubilizing peptides,^[21]^ leading to gelation through aggregation by hydrogen bonding of the DKP moieties.

**Figure 3:**
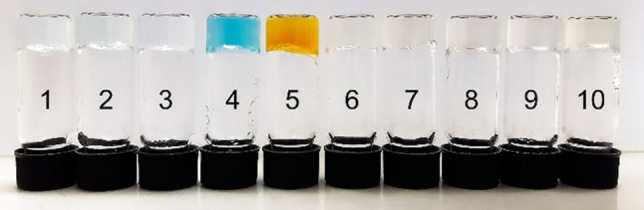
Hydrogels from **1** (1 wt% in H_2_O) and different gelation triggers (3.0 equiv. each), 24 h after addition of the trigger. 1) Zn(OAc)_2_⋅2H_2_O; 2) Ni(OAc)_2_⋅3H_2_O; 3) MgSO_4_⋅7H_2_O; 4) CuSO_4_⋅H_2_O; 5) FeSO4⋅7H_2_O; 6) AcOH; 7) TFA; 8) glucono‐δ‐lactone; 9) *n*Bu_4_ *N*BF_4_; 10) Ph_2_I^+^(OTf)^−^_._

Next, we investigated the hydrogelation properties of the more hydrophilic **1**. To our delight, **1** was able to form pure hydrogels through a sol‐gel process. Heating finely suspended **1** in water at 80 °C for a few minutes caused a seeming dissolution of the solid. A clear sol instead of a solution formed that showed blue fluorescence, indicating aggregation (Figures S34 and S54). Filtering the concentrated sol through a syringe filter (0.3 μm) yielded a filtrate showing no visible fluorescence upon irradiation with a 365 nm UV‐lamp, confirming the presence of large aggregates in the unfiltered sol. The concentration of **1** had no significant influence on the fluorescence emission spectrum (Figure S71). Similarly, draper et al. recently showed the effect of temperature on the solution of a peptide‐based hydrogelator and its gelation properties.^[22]^ We found the sol to be stable for months at room temperature without any gelation or precipitation occurring over time. The addition of different gelation triggers to the sol, such as metal ions, acids or other ions, which have been shown to have dramatic effects on gel properties,^[23]^ then led to hydrogelation and to the formation of translucent or transparent hydrogels (Figure 3).

Diaryliodonium salts are known photo acids.^[24]^ However, the addition of the salt to the sol already led to gelation. When the hydrogel prepared from it was irradiated with UV light, fast decomposition of the gel occurred. Very homogenous gels were obtained by gelation with glucono‐δ‐lactone (GdL).^[25]^ Using GdL, hydrogels of **1** could be obtained with a CGC as low as 0.3 wt% (3.9 mM). The gelation was in all cases not reversible by heating. This indicates a cation exchange or protonation of **1** taking place prior to gelation, forming the much less water‐soluble free acid **7** or different metal salts thereof, which then form the gel network. The indication is supported by the fact that no gels were formed when 0.25 equivalents or less of GdL were added to a 1 wt% sol of **1** in water, indicating that solely the cation‐exchanged species are responsible for the gel formation. All hydrogels (except for those from copper and iron salts) showed blue fluorescence (examples in Figures S34 and S56). The gels remained stable for as long as they were observed (>10 months) when kept from light and tightly closed. Only the Ph_2_I^+^(OTf)^−^ containing gel showed significant sensitivity towards light.

**Figure 4:**
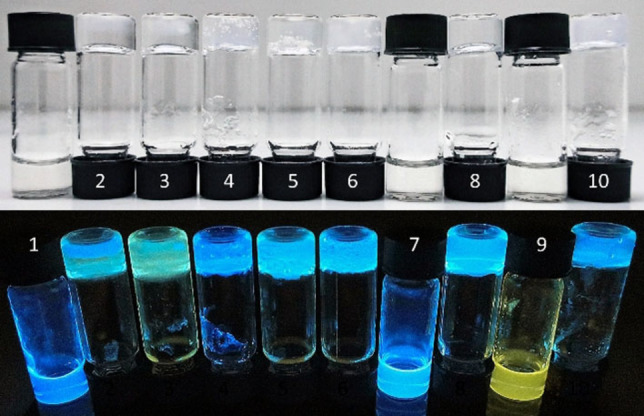
Water/organic solvent mixtures of **1** (10 mg/mL) under ambient light (top) and under 365 nm UV‐light irradiation (bottom), 24 h after heating to 80 °C. Co‐solvents: 1) MeOH; 2) EtOH; 3) *i*‐PrOH; 4) HFIP; 5) acetone; 6) acetonitrile; 7) DMSO; 8) DMF; 9) THF; 10) 1,4‐dioxane.

**1** was insoluble at concentrations of 10 mg/mL in pure organic solvents, even when heated. Therefore, we further explored the gelation properties of **1** in mixed water/organic solvent systems without additives (Table 2, see Table S4 for more details). At room temperature, **1** only dissolved completely in a water/THF mixture. This was indicated by a change in fluorescence emission to a yellow/slightly greenish color and confirmed by filtering the solution, which did not change its fluorescence emission intensity (Figure S51). In contrast, gels formed from mixtures with co‐solvents HFIP, acetone, acetonitrile and 1,4‐dioxane within 17 h of addition of the co‐solvent (Figure S35). Clear sols formed from methanol, DMSO and DMF as co‐solvents. No gels formed in ethanol and *iso*‐propanol mixtures, instead slight precipitation occurred. At 80 °C, solutions were formed from mixtures with methanol, ethanol, *iso*‐propanol and acetone as co‐solvents, as indicated by a change in fluorescence emission from a blue to a yellow/slightly greenish color. The acetonitrile gel and DMF sol both turned to a sol with a yellowish fluorescence containing solid particles showing blue fluorescence, while the HFIP and 1,4‐dioxane gels and the DMSO sol remained largely unchanged at 80 °C (Figure S36). After cooling down, all mixtures formed gels except for the mixtures containing methanol, DMSO and THF (Figure 4 and S37).

**Table 2:** Comparison of gelation properties of **1** and **1**+meglumine in H_2_O/organic solvent mixtures.^[**a**]^

Co‐ solvent	**1** ^[b]^	**1**+ meglumine^[b,c]^	Co‐solvent	**1** ^[b]^	**1**+ meglumine^[b,c]^
methanol	Sol	G	acetonitrile	TG	G
ethanol	TG	G	DMSO	Sol	G
*i*‐PrOH	TG	G	DMF	G	G
HFIP	TG	PG	THF	S	S
acetone	G	G	1,4‐dioxane	TG	TG

[a] A co‐solvent (500 μL) was added to a solution of **1** (10 mg) in H_2_O (500 μL). The mixture was left standing for 17 h, then heated to 80 °C for 10 min, then left standing for 24 h. [b] G: transparent gel; TG: translucent gel; S: solution; PG: partly gel. [c] With 10 mg/mL meglumine. For more detailed gelation properties, see Tables S4, S5.

The gels from mixtures containing ethanol, *iso*‐propanol, acetone, acetonitrile and DMF showed reversible gelation by heating to 80 °C and then resting at room temperature for up to 17 h (Table S4).

Full gelation of the mixtures did not necessarily afford the same blue fluorescence indicating aggregation as seen in solid **1**, its sol or its GdL gel, as exemplified by the water/*i*‐PrOH gel, which showed a much greener fluorescence. This could either be a solvent effect or might indicate partly disaggregated (solvated) **DPhCzT** moieties within the gel.

When experimenting with additives for these mixtures, we found that addition of more meglumine (10 mg/mL) to the sol of **1** increased its gelation abilities as well as the transparency of the obtained gels in all mixtures except for the water/HFIP mixture (Figure S38, Table 2, see Table S5 for more details).

**Figure 5:**
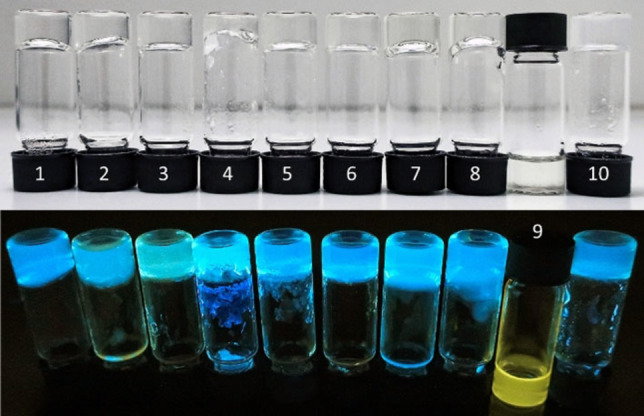
Water/organic solvent mixtures of **1**+meglumine (both 10 mg/mL) under ambient light (top) and under 365 nm UV‐light irradiation (bottom), 24 h after heating to 80 °C. (Co‐solvents: 1) MeOH; 2) EtOH; 3) *i*‐PrOH; 4) HFIP; 5) acetone; 6) acetonitrile; 7) DMSO; 8) DMF; 9) THF; 10) 1,4‐dioxane.

Initially, there were only minor differences between the mixtures with and without meglumine 17 h after addition of the co‐solvents. While the same co‐solvents caused gelation and sol formation, the main differences were slight precipitate formation with the co‐solvent HFIP and a weaker gel resulting from the co‐solvent acetonitrile (Figure S38). However, when these mixtures were heated to 80 °C, the same yellow/greenish fluorescence emission indicating solution was observed in all mixtures except for the HFIP mixture, which contained insoluble blue fluorescent particles (Figure S39). 17 h after cooling down, all mixtures except for the water/THF mixture had formed gels (Figure 5 and S40).

While **1** alone could not form gels in a water/methanol or water/DMSO mixture at 10 mg/mL, transparent gels were formed with additional meglumine. Moreover, samples of **1** with additional meglumine formed transparent gels with ethanol, isopropyl alcohol and acetonitrile, whereas **1** alone formed translucent gels (Table 2). Notably, the HFIP containing mixture gelated only partly, dividing into gel and a gel‐like precipitate. Gelation was reversible for all gels with added meglumine under heating except for the insoluble precipitate formed in the water/HFIP mixture.

### Fluorescence measurements

**Figure 6:**
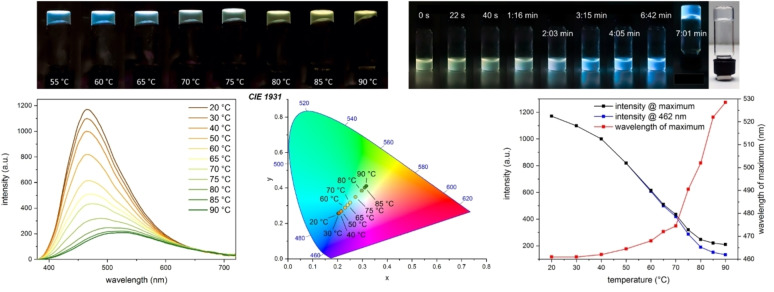
(Top left) temperature dependent fluorescence of the gel of **1** (10 mg/mL) from water:acetonitrile (1 : 1, with 10 mg/mL meglumine) while heating, and while cooling down (top right). (Left) temperature dependent fluorescence emission spectra while heating. (Middle) CIE 1931 chromaticity plot of the temperature dependent fluorescence emission spectra. For the table of emission color coordinates, see Figure S64. (Right) temperature dependence of the maximum, the aggregation maximum (462 nm) and the wavelength of the maximum of the gel above. Excitation at 365 nm in all cases.

To quantify these observations, we initially measured the temperature dependent fluorescence emission spectra of the water/acetonitrile gel of **1** (10 mg/mL) with added meglumine (Figure 6). With increasing temperature, the fluorescence emission intensity decreased and its maximum became more redshifted. Above 70 °C, the red‐shifting of the maximum accelerated noticeably with higher temperatures and the fluorescence emission spectrum converged to almost the same spectrum as that of **1** dissolved in water/THF, indicating solution. The temperature dependent behavior of the gel was also studied (Figures 6 and S62‐S65). It melted completely after 10 min at 90 °C, beginning very slowly at 80–85 °C, although a noticeable shift in fluorescence emission was already visible at 70 °C (Figure S65). Upon cooling down, the solution quickly formed a gel after the fluorescence emission had changed back to blue, which indicated aggregation (Figure S66). This shows that the gel structure remains stable for a short time even after almost complete disaggregation of the **DPhCzT** moieties, as shown by the fluorescence spectra. It further implies that the gel network is mainly stabilized by the hydrogen‐bonding of the DKP ring systems. To rule out that the change in fluorescence emission is only an effect of temperature, the experiment was repeated with the GdL gel of **1** (1 wt% in H_2_O). For this gel, gelation was not reversible by heating and it showed a decrease of fluorescence intensity with increasing temperature, but no shift of the fluorescence emission (Figures S68 and S69). In this case, the gel is possibly mainly stabilized by the hydrophobic interactions of the **DPhCzT** moieties, which are driven to aggregate by the aqueous environment. Both results are in good alignment with findings by makarević et al., who found that gels of bis(amino acid) oxalyl amides were stabilized mainly by lipophilic interactions in aqueous environments and by intermolecular hydrogen bonding in organic solvents.^[26]^

The effect of aggregation on the fluorescence emission could also be seen for water/THF solutions of **1**. Adding GdL to a filtered solution of **1** (6.7 mg/mL) in 33 % THF and 67 % water or **1** (6.0 mg/mL) in a 40 % THF and 60 % water led to a change in fluorescence emission from slightly greenish/yellow to blue within seconds and rose well until after a gel was formed.

**Figure 7:**
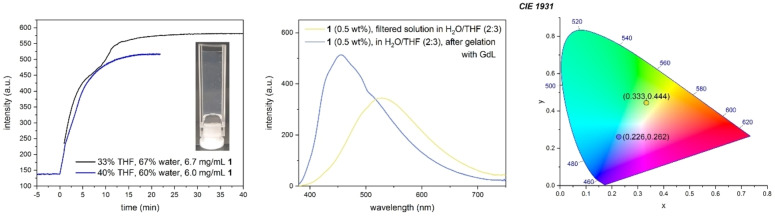
(Left) evolution of the fluorescence emission intensity of the aggregation maximum (456 nm) of a filtered water/THF solutions of **1** after addition of GdL (12 equiv. at *t*=0), and picture showing the resulting gel 1 h after addition of GdL (40/60 % mixture, ambient light). (Middle) fluorescence emission spectrum of the 40/60 % mixture before and 1 h after the addition of GdL, showing AIBSE. (Right) CIE 1931 chromaticity plot of the spectra. Excitation at 365 nm for all spectra.

Through this effect, the gel formation could be tracked via monitoring the fluorescence emission intensity of the aggregation‐induced maximum over time, directly after the addition of GdL (Figure 7).

The form of the observed curve for the 33/67 % mixture is in excellent agreement with results from chen et al., who investigated the self‐assembly mechanism for a naphthalene‐dipeptide hydrogelator. The mixture shows the same two plateaus that were found during the hydrogelation of a naphthalene‐dipeptide hydrogelator with GdL, whereas the first plateau corresponded to a drop of the pH of the mixture to that of the pk_a_ of the carboxylic acid group of the peptide.^[27]^ In their work, the authors used thioflavin T (ThT) to track the aggregation of the peptides and the gelation process via the fluorescence emission of ThT, which was incorporated into the gel during gelation. By incorporation into the gel, it showed an increase in fluorescence intensity due to restricted intramolecular rotation (RIR).^[28]^ Furthermore, the AIBSE phenomenon has been shown to be caused by RIR in a theoretical study.^[4]^ This implies that the observed AIBSE in our case is caused by RIR of the **DPhCzT** moieties, giving direct experimental evidence for the theoretical study.

The results also show that even a very slight change in solvent composition can cause huge differences in the self‐assembly process, as the 40/60 % mixture aggregates too fast for the plateau to really show despite a slightly lower concentration of **1**. The resulting gels were only stable for two days, after which they both degraded to a solution containing a voluminous precipitate.

**Figure 8:**
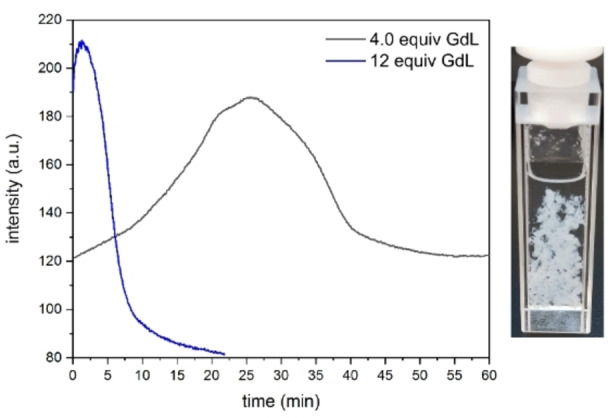
Evolution of the fluorescence emission intensity of the aggregation maximum (456 nm; excitation at 365 nm) of a filtered water/THF (1 : 1) solution of **1** (5 mg/mL) after addition of GdL, and picture (ambient light) showing the resulting suspension 1 h after addition of 12 equivalents GdL.

When the ratio of THF to water was further raised to 50 : 50, no gel formed. Instead, addition of GdL to this solution led to precipitate formation shortly after (Figure 8). Addition of 12 equivalents of GdL resulted in a very fast increase of fluorescence emission intensity at 456 nm within 5 min, followed by a sharp decline to a lower intensity than before the addition due to the scattering of light by the precipitate.

The addition of 4.0 equivalents of GdL instead resulted in a much slower increase in fluorescence emission intensity, but led to the same result. The at first accelerating increase was significantly slowed down 20 min after the addition and declined significantly between 25 min and 40 min to a level similar to that before the addition, which was also due to the formation of light‐scattering precipitate. The explanation for the drastic effect of the THF content on the gelation process can be rationalized by the better solvation of the **DPhCzT** moieties with a higher THF content. If the content is too high, the gel state, which presents a kinetic trap for aggregation, can be overcome and the thermodynamic minimum (precipitation) reached.^[29]^

**Figure 9:**
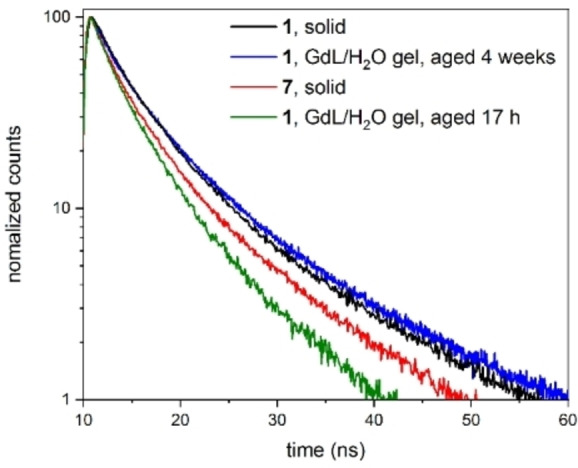
Comparison of fluorescence lifetime decay profiles (excitation at 373 nm). Comparison of GdL gels of **1** to the solids **1** and **7**. For an enlarged picture see Figure S74.

To further investigate the aggregation of the **DPhCzT** moieties, fluorescence lifetimes as well as quantum yields of the solids and selected gels were measured, and the radiative and non‐radiative decay rates *k*_r_ and *k*_nr_ were calculated (Table 3). **1** showed an average fluorescence lifetime of *τ*_avg_=(6.30±0.16) ns as a solid, a slightly shorter lifetime of *τ*_avg_=(5.93±0.13) ns as a sol in water and a significantly shorter lifetime of *τ*_avg_=(4.37±0.14) ns as 17 h old GdL gel (Figure 9). For **2**, the same reduction in fluorescence lifetime was observed after gelation as for **1** (Table 3, Figure S76).

**Table 3:** Overview of photophysical properties of selected compounds and formulations. For detailed formulations, see chapter 10.1 of the Supporting Information.

Compound	Entry	*λ*_em_ [nm]^[a]^	*τ*_avg_ [ns]^[b]^	***Φ*_F_** [%]^[c]^	*k*_r_ [10^8^ s^−1^]^[d]^	*k*_nr_ [10^8^ s^−1^]^[d]^
**1**	Solid	465	6.30±0.16	3.9	0.062	1.53
**2**	Solid	466	5.54±0.13	3.8	0.069	1.74
**7**	Solid	463	5.46±0.17	3.0	0.055	1.78
**1**	Solution in THF/H_2_O (2/3) (6.0 mg/mL)	527	4.67±0.35	1.2	0.026	2.12
**1**	H_2_O sol (1 wt%)	466	5.93±0.13 (10.3±0.1)^[e]^	3.5	0.059	1.63
**1**	GdL/H_2_O gel 17 h old (1 wt%)	462	4.37±0.14	2.5	0.057	2.23
**1**	GdL/H_2_O gel 4 weeks old (1 wt%)	462	6.61±0.08	2.3	0.035	1.48
**1**	H_2_O/MeCN+meglumine gel (10 mg/mL)	462	6.45±0.24	2.7	0.042	1.51
**2**	H_2_O/HFIP+TFA gel 17 h old (10 mg/mL)	445	4.37±0.11	1.9	0.043	2.24

[a] Excitation at 365 nm. [b] Excitation at 373 nm. [c] The absolute error of the measurement was Δ*Φ*=±0.1 %. [d] *k*_r_ and *k*_nr_ were calculated using the equations *k*_r_=*Φ*_F_/*τ*_avg_ and *k*_nr_=(1−*Φ*_F_)/*τ*_avg_. [e] Average fluorescence lifetime when deaerated. For spectroscopic data of diluted solutions of **1** and **2** in organic solvents, see Tables S2 and S3.

As found before by hydrogelation experiments, the GdL gel of **1** should consist mainly of the free acid **7** since substoichiometric amounts of GdL did not cause gelation. However, after aging for four weeks, the lifetime of the GdL gel increased to *τ*_avg_=(6.61±0.08) ns, which is longer than that of **7** (*τ*_avg_=(5.46±0.17) ns) and very similar to solid **1**, indicating a similar microenvironment. The at first significantly decreased lifetime after gelation can be rationalized by a competing aggregation of the **DPhCzT** moieties on the one hand and the DKP ring systems on the other hand. First, the gel structure forms through hydrogen bonding of the DKP ring systems, leaving aggregates of the **DPhCzT** moieties out of equilibrium compared to the sol. Over time, the gel structure reorganizes to a local kinetic minimum at which aggregation interactions are maximized, leading to conformationally stabilized excited states, explaining the longer lifetime of the aged gel. The water/acetonitrile gel of **1** with added meglumine, in contrast, showed a significantly increased fluorescence lifetime 17 h after gelation compared to the sol of **1** in water. A recent study by debnath et al. has shown that π‐stacking dominates hydrogen bonding at higher temperatures for the gelation of Fmoc‐tyrosyl‐leucine.^[30]^ In our case, this could be a possible explanation for the increased lifetime, as dominating π‐stacking would then lead to initially conformationally stabilized excited states. A second explanation for the increased lifetimes of the aged GdL gel and the water/acetonitrile+meglumine gel of **1** might be a partial displacement of oxygen from the gel structure. The effect of oxygen on the fluorescence lifetime of **1** was shown by deaerating the aqueous sol of **1**, which led to an almost doubling of the lifetime (Table 3). A partial displacement of oxygen could take place through the formation of hydrophobic domains within the gel after gelation, and the effect increased by further rearrangement of the **DPhCzT** moieties after gelation as described above.

The fluorescence quantum yields were highest in the solid state and in the aqueous sol of **1** in water, ranging between 3.0 % and 3.9 %, and were in all cases decreased in the gel state, ranging from 1.9 % to 2.7 %. Interestingly, although the H_2_O/GdL gel of **1** showed a significantly increased average fluorescence lifetime, it also showed significantly decreased radiative and non‐radiative decay rates after aging for four weeks and a slightly decreased quantum yield (2.5 % before aging and 2.3 % afterwards, which is within the error range of the measurement and might be attributed to slight degradation of the gel, although gels were found to be stable for months). The H_2_O/MeCN+meglumine gel of **1** shows similar behavior with decreased radiative and non‐radiative decay rates and quantum yield compared to the sol and solid of **1**, despite the increased fluorescence lifetime. The highest non‐radiative decay rates were observed in the THF/H_2_O solution of **1**, in the 17 h old H_2_O/GdL gel of **1** and in the H_2_O/HFIP+TFA gel of **2** and were very similar (2.12 ⋅ 10^8^ s^−1^ to 2.24 ⋅ 10^8^ s^−1^). This further indicates that a concentrated aqueous solution or a freshly prepared gel which is dominated by hydrogen bonding leads to (initially) conformationally destabilized excited states, while a prolonged time after gelation or after gelation at high temperatures leads to a gel with favorable π‐stacking and conformationally stabilized excited states.

### Material properties

**Figure 10:**
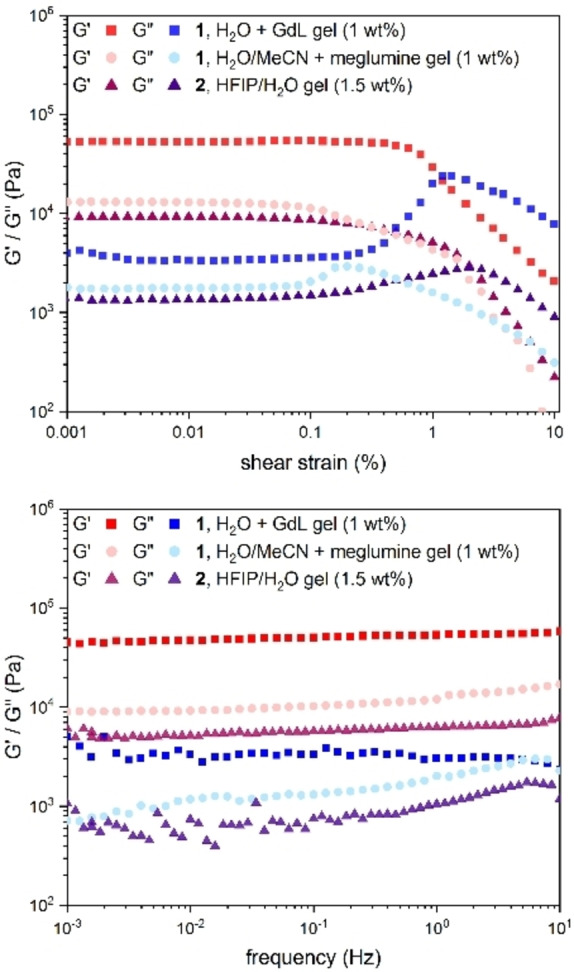
Comparison of frequency sweeps of the GdL (12 equiv.) gel of **1** (1.5 wt%), the water/acetonitrile (1 : 1)+meglumine (10 mg/mL) gel of **1** (10 mg/mL) and of the water/HFIP (1 : 1)+TFA (1 %) gel of **2** (15 mg/mL). All gels aged 7 days prior to the measurement. Single pictures are also in the Supporting Information, Figures S82‐S84.

Rheological experiments were performed on the GdL/water gel of **1**, the water/acetonitrile+meglumine gel of **1** and the water/HFIP+TFA gel of **2** (Figure 10).

Despite showing gel formation by tube‐inversion already 30 min after addition of GdL, the resulting gel was too unstable to be transferred to the rheometer even after 17 h. However, after aging for several days, the gel could be transferred and measured without problems. Amplitude sweep experiments showed a mechanical stability for all gels, aged one week, up to 0.1 % shear strain. The experiments confirmed that all gels were physical hydrogels and not viscoelastic fluids since the storage modulus *G’* was in all cases about one order of magnitude higher than the loss modulus *G”*. Frequency sweep experiments further showed only a weak dependency of *G’* and *G”* on frequency, which are both in a range common for DKPs with aromatic residues.[^10a^, ^10h^, ^14a^, ^14b^]

**Figure 11:**
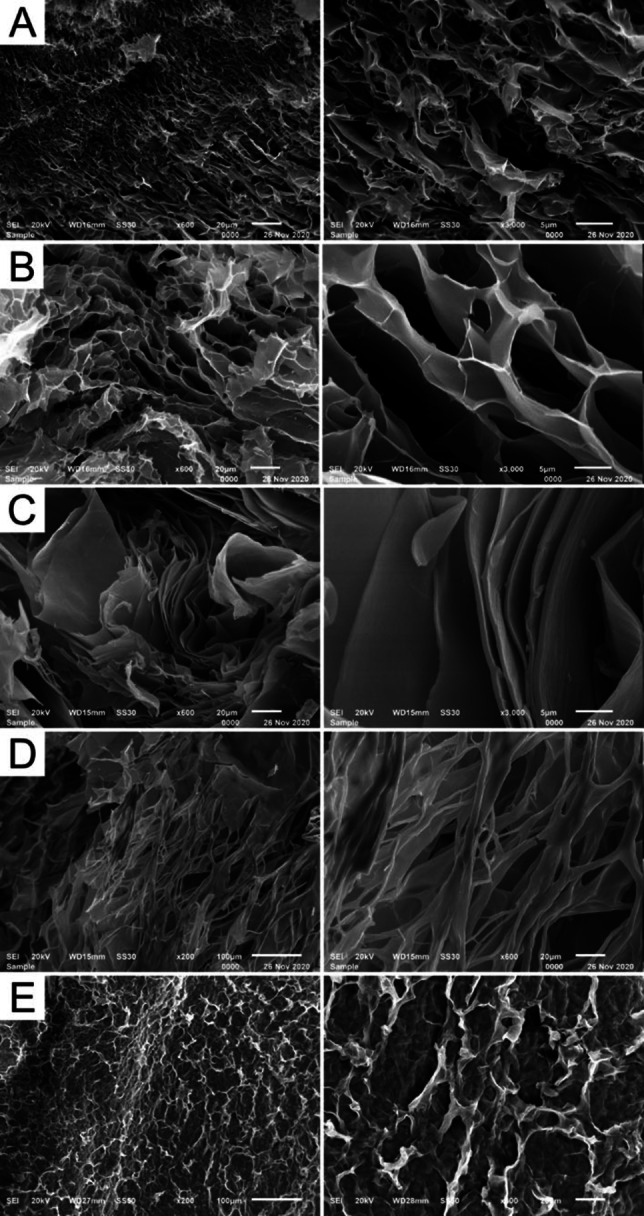
SEM images of select xerogels of **1** and **2**. A) Freeze dried sol of **1** in water (1 wt%); B) *n*Bu_4_NBF_4_ (3.0 equiv.) gel of **1** (1 wt%) in water, aged four weeks; C) GdL gel of **1** (1 wt%, 12 equiv. GdL), aged 7 d; D) water/acetonitrile (1 : 1) gel of **1** (10 mg/mL) with additional meglumine (10 mg/mL), aged 7 d; E) water/HFIP (1 : 1)+TFA (1 %) gel of **2** (10 mg/mL), aged 7d. For detailed formulations, see chapter 10.1 of the Supporting Information. For bigger pictures, see Figures S77–S81.

Selected gels and the sol of **1** in water were further analyzed for their microstructures by performing scanning electron microscopy (SEM) after freeze‐drying (Figure 11). The freeze‐dried sol of **1** showed a highly porous network of thin ribbons, as an effect of its aggregation and possible preorganization in water before freeze‐drying (Figure 11A) The ribbons were approx. 60 nm ‐ 200 nm thick (with most around ∼100 nm). The *n*Bu_4_ *N*BF_4_ gel of the same sol in comparison shows significantly thicker ribbons (approx. 100 nm ‐ 400 nm) with visible fibers, cross‐linked to a gel‐network and demonstrating the difference between the hydrogel and the viscous sol (Figure 11B). In stark contrast, the GdL/water gel of **1** is characterized by very long lamellar sheets that are approx. 0.1 μm–1 μm thick (average around 0.4 μm, Figure 11C). The water/acetonitrile+meglumine gel of **1** and the water/HFIP+TFA gel of **2** both consisted of a network of much thicker fibers, with a thickness above 1 μm and up to 4 μm for the gel of **2**, and up to 10 μm for the gel of **1** (Figures 11D and 11E).

## Conclusion

In conclusion, our work demonstrates the efficient hydrogelation of minimalistic, DKP‐substituted fluorophores. Although the heterocyclic luminophore **DPhCzT** made up 51.1 % of the mass of hydrogelator **1** and 57.8 % of the mass of gelator **2**, efficient hydrogelation by many triggers was possible for **1** with a CGC down to 0.3 wt% (3.9 mM). Also, a wide variety of organogels could be formed by **2** at a concentration of 1 wt% or below. Aggregation properties were investigated through different fluorescence techniques. The results show the importance of solvent choice, additives and temperature for the aggregation event and the resulting fluorescence properties. Moreover, the delicate structural balance needed between hydrophilic and hydrophobic aggregation interactions to avoid competing interactions is demonstrated. We believe our work demonstrates the usefulness of the minimalistic, modular system consisting of a DKP and a luminophore for the synthesis and study of efficient hydrogelators containing challenging hydrophobic fluorophores. Quick and manifold structural variations are easy possible for further fine‐tuning the gel properties through variation of the amino acid side‐chains or counter‐ions.

## Experimental Section

For full experimental procedures as well as spectroscopic and analytical data for all compounds including copies of NMR spectra, see the Supporting Information.

## Conflict of interest

The authors declare no conflict of interest.

## Supporting information

As a service to our authors and readers, this journal provides supporting information supplied by the authors. Such materials are peer reviewed and may be re‐organized for online delivery, but are not copy‐edited or typeset. Technical support issues arising from supporting information (other than missing files) should be addressed to the authors.

Supporting Information
