# Supplementary material for: Efficient Hydro‐ and Organogelation by Minimalistic Diketopiperazines Containing a Highly Insoluble Aggregation‐Induced, Blue‐Shifted Emission Luminophore
Source: Chemistry. 2021 Oct 22;27(66):16488–97. doi: 10.1002/chem.202102861 (PMC9297864; doi:10.1002/chem.202102861)
Supplement: Supplementary file 1 — Supporting Information [file CHEM-27-16488-s001.pdf]

Chemistry–A European Journal

Supporting Information

Efficient Hydro- and Organogelation by Minimalistic Diketopiperazines Containing a Highly Insoluble Aggregation-Induced, Blue-Shifted Emission Luminophore

Martin Molkenthin, Werner M. Nau,* and Boris J. Nachtsheim*

Table of content

1 General Information ... 4

2 Overview of the synthetic routes for gelators 1 and 2 ... 7

3 Overview of synthesized DPhCzT derivatives ... 9

4 Aggregation-induced blue-shifted emission properties of 1 and 2 ... 10

4.1 Aggregation-induced blue-shifted emission of 1 and 2 in DMSO/water mixtures ... 10

4.2 Aggregation-induced blue-shifted emission of 1 and 2 in HFIP/water mixtures ... 12

4.3 Photophysical data of 1 and 2 at c = 100 µM ... 14

5 Photophysical properties of 1 and 2 in solution at c = 10 µM ... 16

5.1 Photophysical properties of 1 in solution ... 16

5.2 Photophysical properties of 2 in solution ... 19

6 Gelation properties ... 23

6.1 Gelation experiments with 1 ... 23

6.2 Gelation experiments with 2 ... 29

7 Fluorescence spectra and life-times ... 31

7.1 Fluorescence spectra and life-times of solids ... 31

7.2 Fluorescence spectra and life-times of sols, gels and solutions ... 33

7.3 Temperature dependent fluorescence emission of gels of 1 ... 39

7.4 Concentration dependent absorption- and fluorescence emission spectra of the aqueous sol of 1 ... 43

7.5 Overview and comparison of photophysical data for solids, gels and concentrated solutions ... 44

8 SEM-images of hydro- and organogels ... 47

9 Rheological experiments ... 49

10 Preparation of compounds ... 50

10.1 Overview and preparation of hydro- and organogels 50
10.2 Preparation of L-serine methyl ester hydrochloride (H-Ser-OMe • HCl, S2). 51
10.3 Preparation of *N*-(*tert*-butoxycarbonyl)-L-serine methyl ester (Boc-Ser-OMe, S3) 51
10.4 Preparation of *N*-(*tert*-butoxycarbonyl)-3-iodo-L-alanine methyl ester (S4) .. 52
10.5 Preparation of 3-iodo-9*H*-carbazole (S6) 53
10.6 Preparation of 2-chloro-4,6-diphenyl-1,3,5-triazine (S8) 54
10.7 Preparation of 9-(4,6-diphenyl-1,3,5-triazin-2-yl)-3-iodo-9*H*-carbazole (3)... 55
10.8 Preparation of *N*-(*tert*-butoxycarbonyl)-3-(9-(4,6-diphenyl-1,3,5-triazin-2-yl)-9*H*-carbazol-3-yl)-L-alanine methyl ester (Boc-Ala(3-(DPhCz(3-)T))-OMe) (4) 56
10.9 Preparation of Boc-Lys(Z)-Ala(3-(DPhCz(3-)T))-OMe (S10)........................ 57
10.10 Preparation of cyclo-(Ala(3-(DPhCz(3-)T))-Lys(Z)) (5)................................ 58
10.11 Preparation of *cyclo*-(Ala(3-(DPhCz(3-)T))-Lys) hydrobromide (2)............... 60
10.12 Preparation of Boc-Asp(OBn)-Ala(3-(DPhCz(3-)T))-OMe (S11) 61
10.13 Preparation of *cyclo*-(Ala(3-(DPhCz(3-)T))-Asp(OBn)) (6) 62
10.14 Preparation of *cyclo*-(Ala(3-(DPhCz(3-)T))-Asp) (7).................................... 63
10.15 Preparation of *cyclo*-(Ala(3-(DPhCz(3-)T))-Asp) *N*-methyl-D-glucamine salt (1) 64
11 Difficulties during and troubleshooting of the synthesis.................................... 66
12 References 67
13 NMR spectra 68

1 General Information

Unless otherwise stated, all reactions with moisture- or oxygen-sensitive reagents were performed using standard Schlenk techniques under a nitrogen atmosphere. Reagents were used as received from their commercial supplier. Dry tetrahydrofuran (THF) and dichloromethane (DCM) were obtained from an *inert* PS-MD-6 solvent purification system. Dry DMF was received from a commercial supplier. Yields refer to isolated yields of compounds estimated to be >95% pure as determined by ^{1}H-NMR spectroscopy.

Flash column chromatography was performed on silica gel (0.040 – 0.063 mm) with the solvents given in the procedures.

^{1}H- and ^{13}C-NMR spectra were recorded on a *Bruker* Avance Neo 600 MHz spectrometer and a *Bruker* Avance Neo 600 MHz spectrometer at 23 °C. Chemical shifts for 1H-NMR spectra were reported as *δ* (parts per million) relative to the residual signal of $CHCl_3$ at 7.26 ppm (s), d_6-DMSO at 2.50 ppm (quin), or d_8-THF at 3.58 ppm and 1.72 ppm (both multiplets). Chemical shifts for ^{13}C-NMR spectra were reported as *δ* (parts per million) relative to the signal of $CDCl_3$ at 77.0 ppm (t), d_6-DMSO at 39.5 ppm (sept.), or d_8-THF at 67.2 ppm and 25.3 ppm (both quin.). The following abbreviations were used to describe splitting patterns: br = broad, s = singlet, d = doublet, t = triplet, q = quartet, quin = quintet, sept = septet, m = multiplet. Coupling constants *J* are given in Hertz.

High resolution (HR) EI mass spectra were recorded on the double focusing mass spectrometer ThemoQuest MAT 95 XL from *Finnigan MAT*. APCI mass spectra were recorded on a *Advion* Expression CMS^{L} via ASAP probe or direct inlet. HR-ESI mass spectra were recorded on a *Bruker* impact II mass spectrometer. All signals were reported with the quotient from mass to charge *m/z*.

IR spectra were recorded on a *Nicolet* Thermo iS10 scientific spectrometer with a diamond ATR unit. The absorption bands are reported in cm^{-1}.

Melting points (Mp.) were determined on a *Büchi* M-5600 Melting Point apparatus at a heating rate of 5 °C/min.

UV-vis measurements were performed on a *Shimadzu* UV-2700.

Fluorescence spectra of solids, solutions and gels were recorded on a *Jasco* FP-8300 fluorescence spectrometer at 20 °C. Absolute quantum yields were determined using

a *Jasco* ILF-835 integrating sphere. Temperature dependent fluorescence spectra were recorded on a *Jasco* FP-8500 fluorescence spectrometer and time-resolved fluorescence intensity measurements were recorded on a *Varian* Cary Eclipse fluorescence spectrophotometer at 25 °C. All spectra are corrected. The excitation and emission bandwidth were both 5 nm, the response time 0.2 s, the data interval 0.5 nm and the scan speed 500 nm/min. For the dilution series, the response time was changed to 1 s and the emission bandwidth to 10 nm.

All non-solid samples were measured in quartz cuvettes with a different path length depending on their concentration (UV and fluorescence measurements): 2x10 mm for gels and similarly concentrated formulations, 3x10 mm for 100 µM solutions and 10x10 mm for 10 uM solutions.

Fluorescence life-time decay curves were measured using an *Edinburgh Instruments* FLS-980-st photoluminescence spectrometer with a *PicoQuant* LDH-P-C 375 laser operating at 373 nm as the excitation source (laser pulse length with FWHM ca. 0.69 ns, or a *PicoQuant* PLS-8-2-633 laser operating at 300 nm (laser pulse length with FWHM ca. 1.17 ns). All life-time decay curves were measured at the maximum of the fluorescence emission of each formulation. The lasers were operated by an external *TTi* TGP110 10MHz Pulse Generator at a pulse frequency of 1 MHz. The fluorescence decay curves were fitted by a mono-, bi- or tri-exponential decay using the formula:

$$f(t) = A + \sum_{i=1}^{\mathrm{n}} B_i e^{-\frac{t}{\tau_i}}$$

With n = 1 for mono, n = 2 for bi-, and n = 3 for tri-exponential decays. Fitting was done *via* an exponential tail fit. The timeframe used for fitting was the time after the intensity of the instrument response function reached <1% intensity to the time the count rate reached ca. 10^1 counts. Average fluorescence life-times were calculated using the formula:

$$\tau_{avg} = \frac{\sum_{i=1}^{n} B_i \tau_i}{\sum_{i=1}^{n} B_i}$$

Rheological experiments were performed on a *Malvern Instruments* Kinexus pro+ rheometer. The geometry was plate-plate with a diameter of 8 mm. All experiments were performed at 22 °C. A solvent trap filled with water was used in all cases.

Amplitude sweep experiments were performed at an angular frequency of 10 rad/s between strains of 0.001% - 10%. Frequency sweep experiments were performed from 0.001 Hz - 10 Hz under a strain of 0.015%, which was within the linear viscoelastic region (LVER) of all gels.

2 Overview of the synthetic routes for gelators 1 and 2

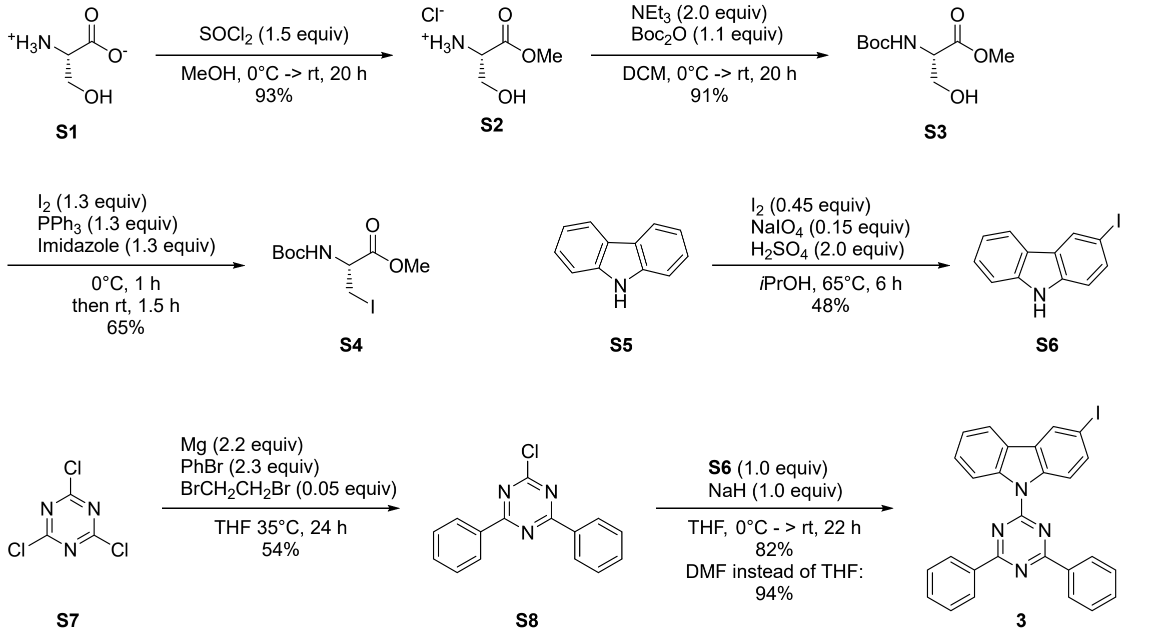

Scheme S1. Synthesis of precursors **3** and **S4**.

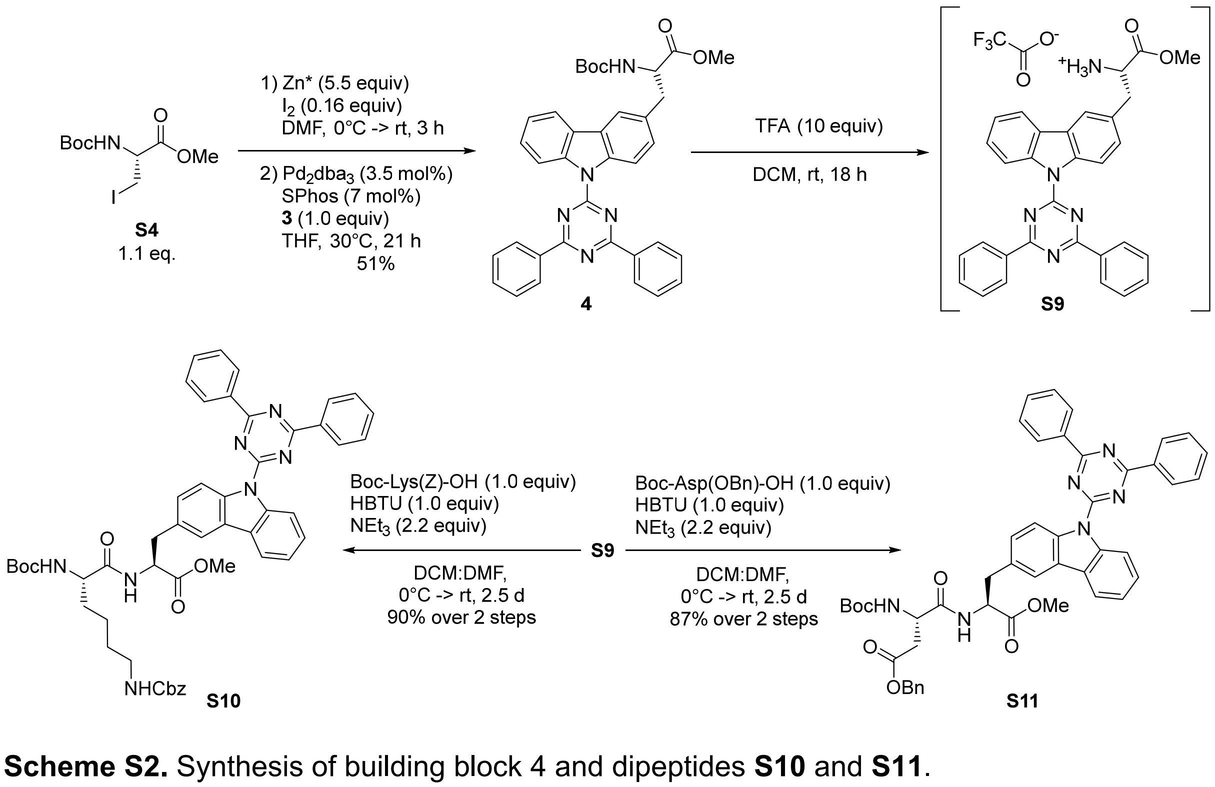

Scheme S2. Synthesis of building block 4 and dipeptides **S10** and **S11**.

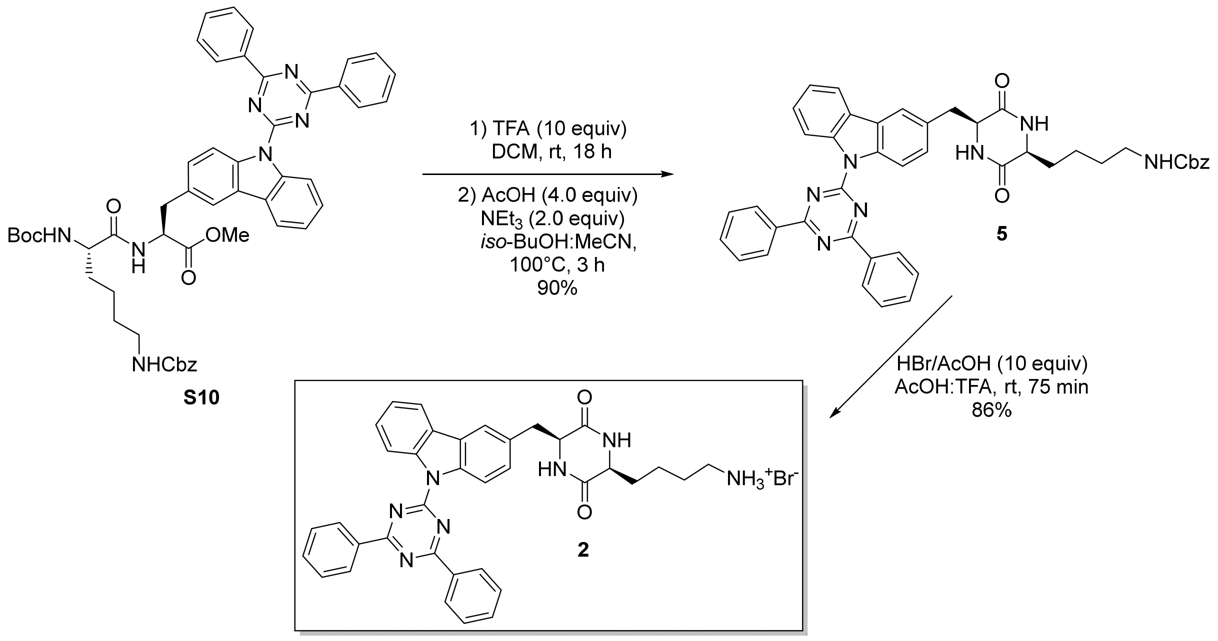

Scheme S3. Synthesis of lysine-derived DKP **2** from dipeptide **S10**.

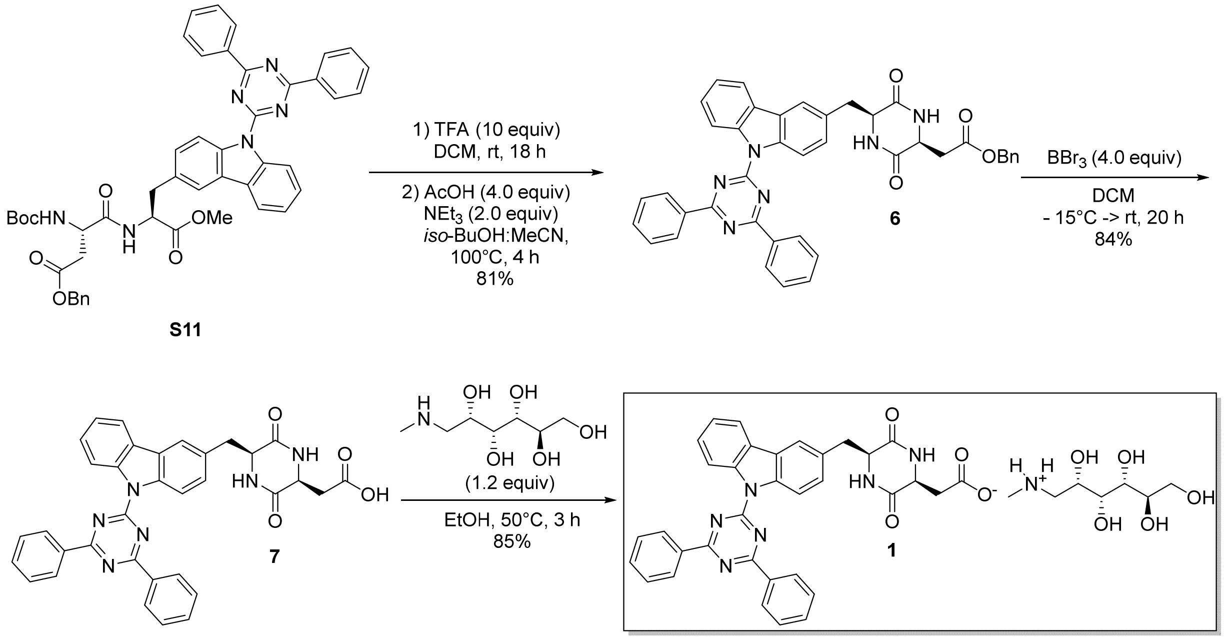

Scheme S4. Synthesis of aspartic acid-derived DKP **1** form dipeptide **S11**.

Note: See chapter 11 for a brief summary of difficulties and troubleshooting of the synthesis.

3 Overview of synthesized DPhCzT derivatives

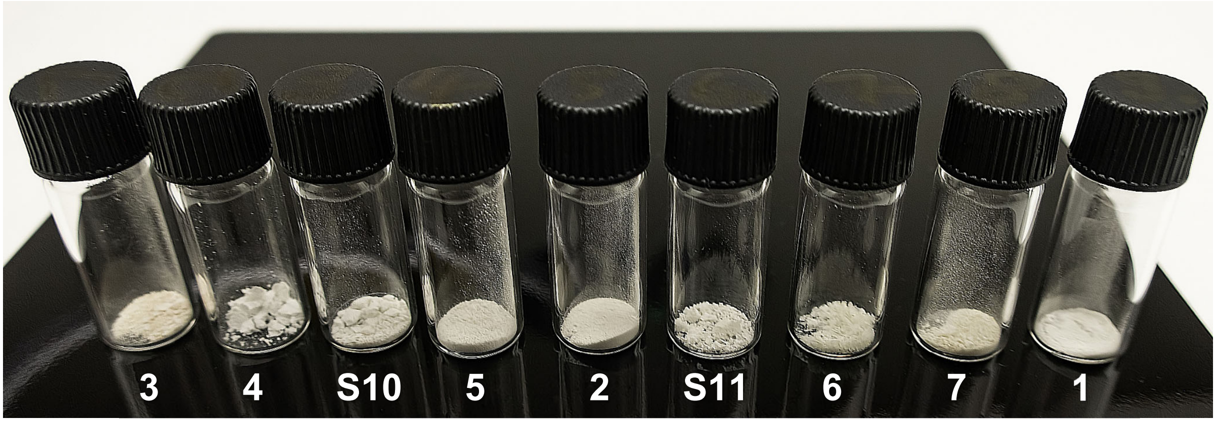

Figure S1. Synthesized **DPhCzT** derivatives in this work at daylight.

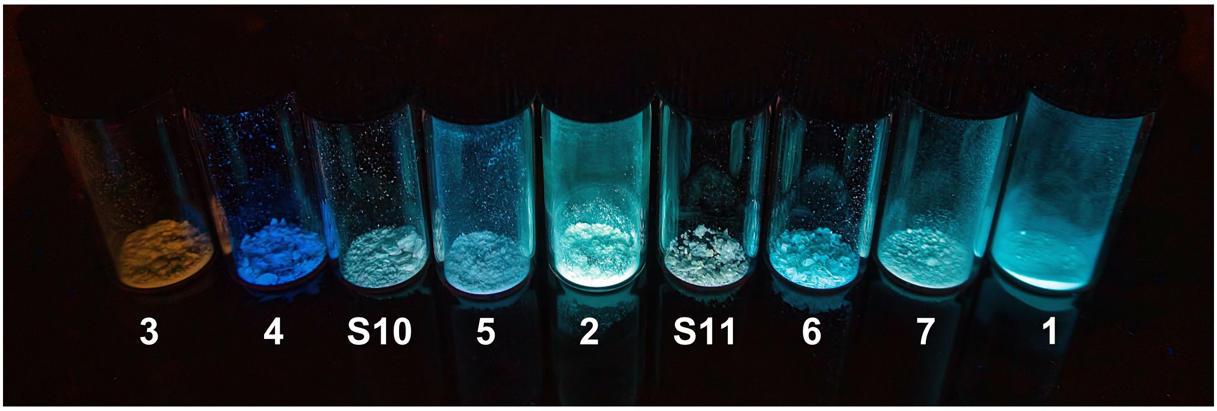

Figure S2. Synthesized **DPhCzT** derivatives in this work under 365 nm UV-light irradiation.

4 Aggregation-induced blue-shifted emission properties of 1 and 2

4.1 Aggregation-induced blue-shifted emission of 1 and 2 in DMSO/water mixtures

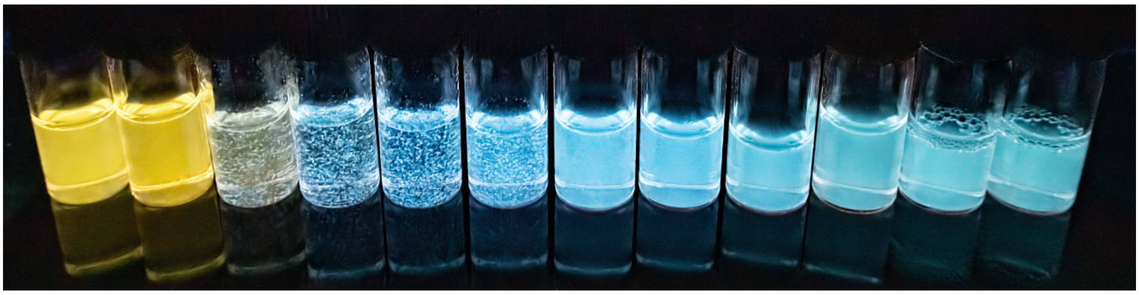

Figure S3. DMSO/water mixtures of **1** (100 µM) upon UV-light irradiation (365 nm). Water content from left to right: 0-10-20-30-40-50-60-70-80-90-95-99%. In some cases, a precipitate formed.

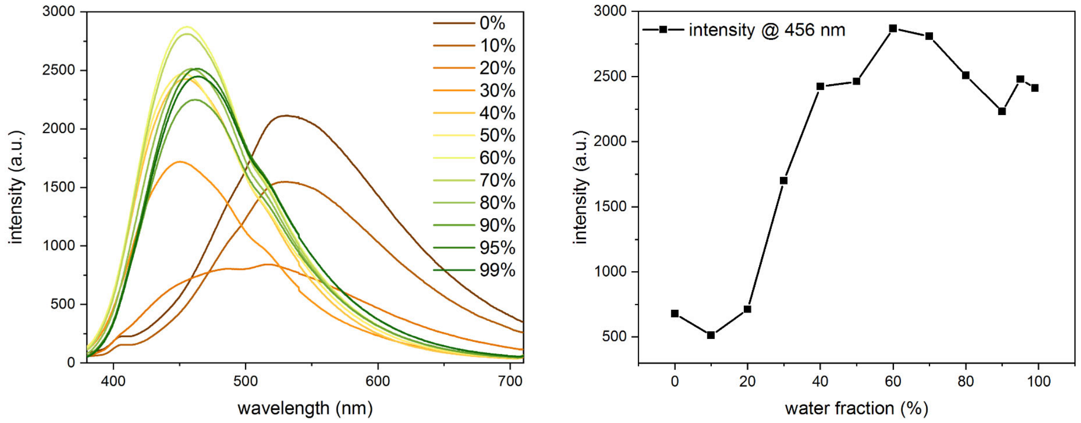

Figure S4. Aggregation of **1** in DMSO/water mixtures (c = 100 µM) by increasing the water content. Fluorescence emission spectra (left, water content in %) and plotting of the intensity at the emerging maximum (456 nm) vs the water content (left). Excitation at 365 nm.

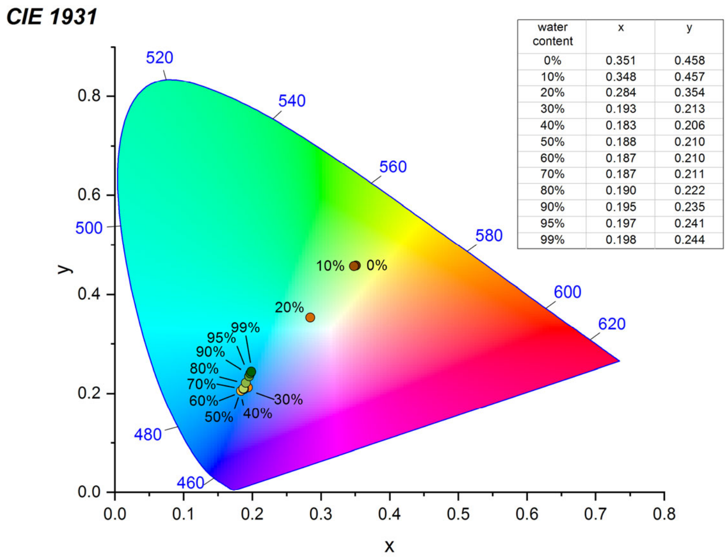

water content	x	y
0%	0.351	0.458
10%	0.348	0.457
20%	0.284	0.354
30%	0.193	0.213
40%	0.183	0.206
50%	0.188	0.210
60%	0.187	0.210
70%	0.187	0.211
80%	0.190	0.222
90%	0.195	0.235
95%	0.197	0.241
99%	0.198	0.244

Figure S5. CIE 1931 chromaticity plot with table of emission color coordinates.

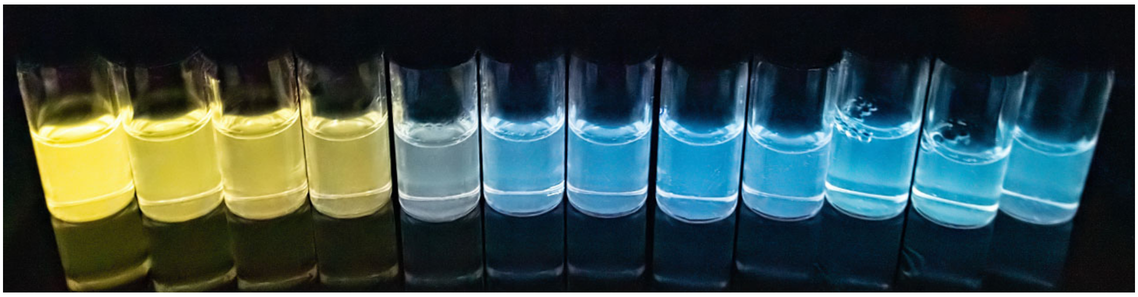

Figure S6. DMSO/water mixtures of **2** (100 µM) upon UV-light irradiation (365 nm). Water content from left to right: 0-10-20-30-40-50-60-70-80-90-95-99%.

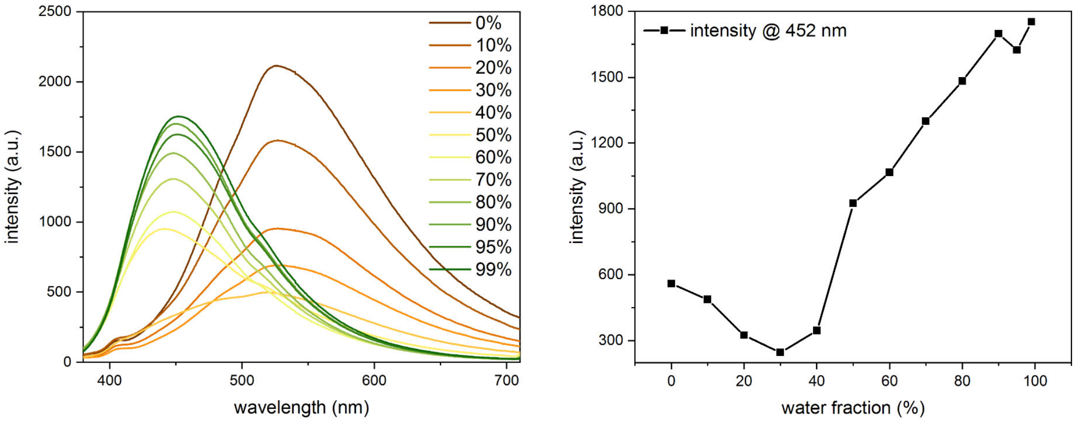

Figure S7. Aggregation of **2** in DMSO/water mixtures (c = 100 µM) by increasing the water content. Fluorescence emission spectra (left, water content in %) and plotting of the intensity at the emerging maximum (452 nm) vs the water content (left). Excitation at 365 nm.

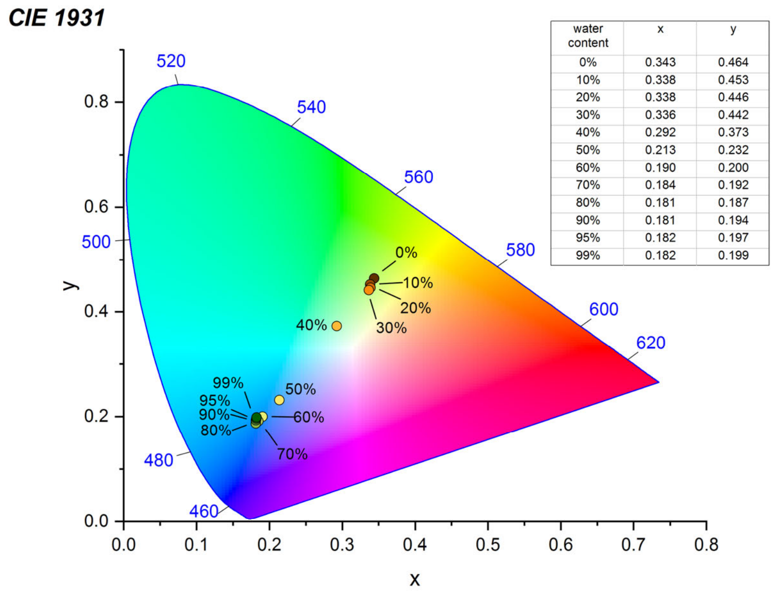

water content	x	y
0%	0.343	0.464
10%	0.338	0.453
20%	0.338	0.446
30%	0.336	0.442
40%	0.292	0.373
50%	0.213	0.232
60%	0.190	0.200
70%	0.184	0.192
80%	0.181	0.187
90%	0.181	0.194
95%	0.182	0.197
99%	0.182	0.199

Figure S8. CIE 1931 chromaticity plot with table of emission color coordinates.

4.2 Aggregation-induced blue-shifted emission of 1 and 2 in HFIP/water mixtures

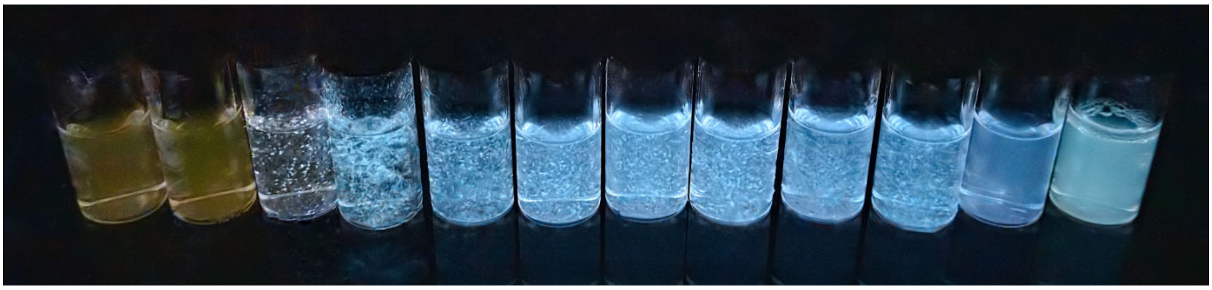

Figure S9. HFIP/water mixtures of **1** (100 µM) upon UV-light irradiation (365 nm). Water content from left to right: 0-10-20-30-40-50-60-70-80-90-95-99%. In some cases, a precipitate formed.

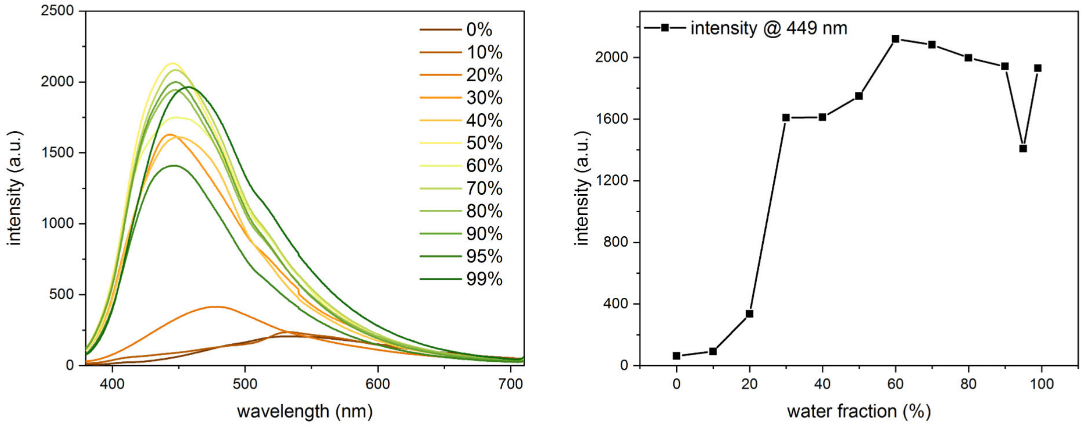

Figure S10. Aggregation of **1** in HFIP/water mixtures (c = 100 µM) by increasing the water content. Fluorescence emission spectra (left, water content in %) and plotting of the intensity at the emerging maximum (449 nm) vs the water content (left). Excitation at 365 nm.

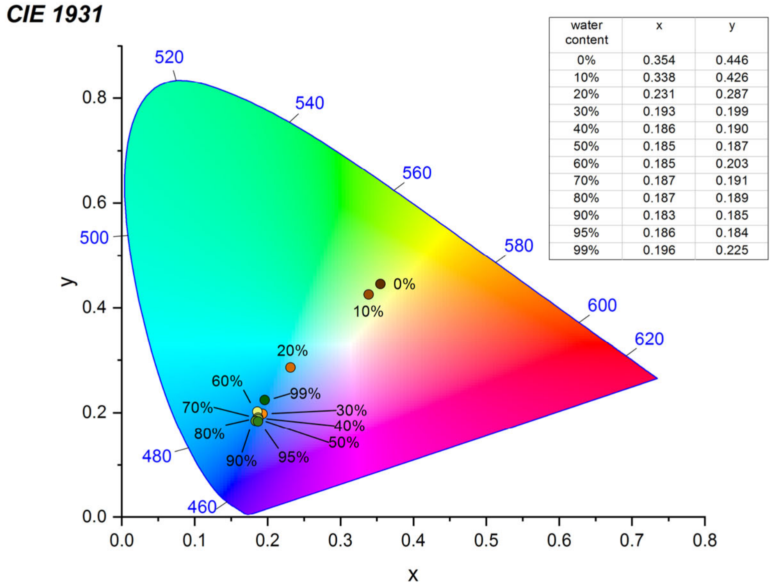

water content	x	y
0%	0.354	0.446
10%	0.338	0.426
20%	0.231	0.287
30%	0.193	0.199
40%	0.186	0.190
50%	0.185	0.187
60%	0.185	0.203
70%	0.187	0.191
80%	0.187	0.189
90%	0.183	0.185
95%	0.186	0.184
99%	0.196	0.225

Figure S11. CIE 1931 chromaticity plot with table of emission color coordinates.

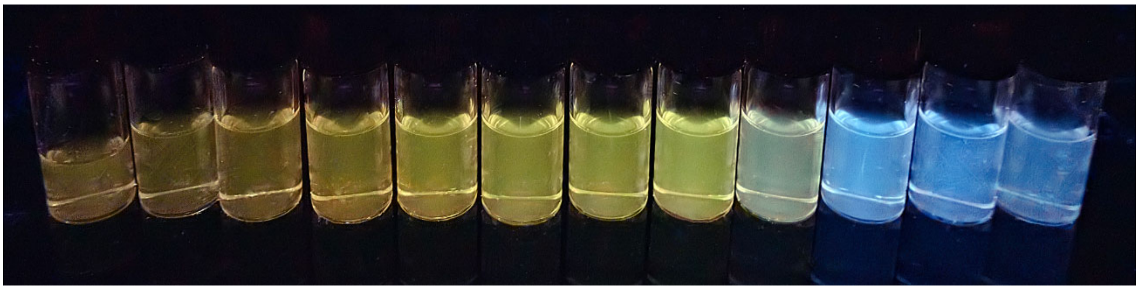

Figure S12. HFIP/water mixtures of **2** (100 μM) upon UV-light irradiation (365 nm). Water content from left to right: 0-10-20-30-40-50-60-70-80-90-95-99%.

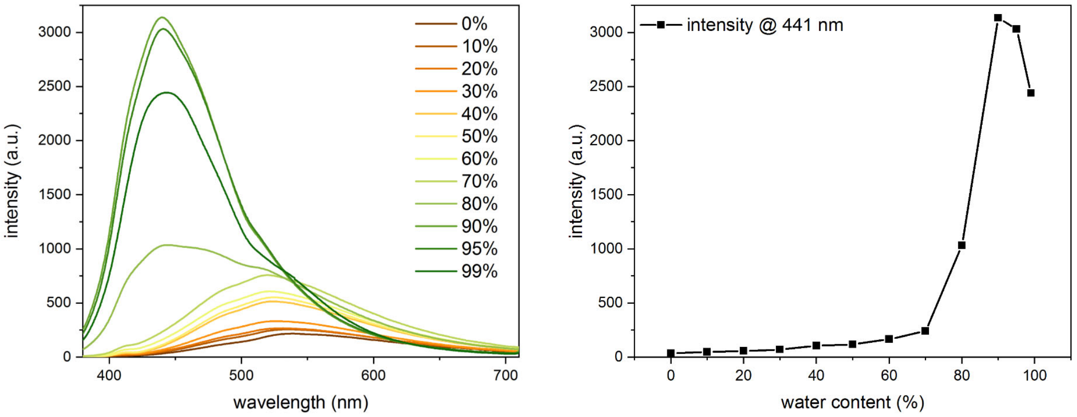

Figure S13. Aggregation of **2** in HFIP/water mixtures (c = 100 μM) by increasing the water content. Fluorescence emission spectra (left, water content in %) and plotting of the intensity at the emerging maximum (441 nm) vs the water content (left). Excitation at 365 nm.

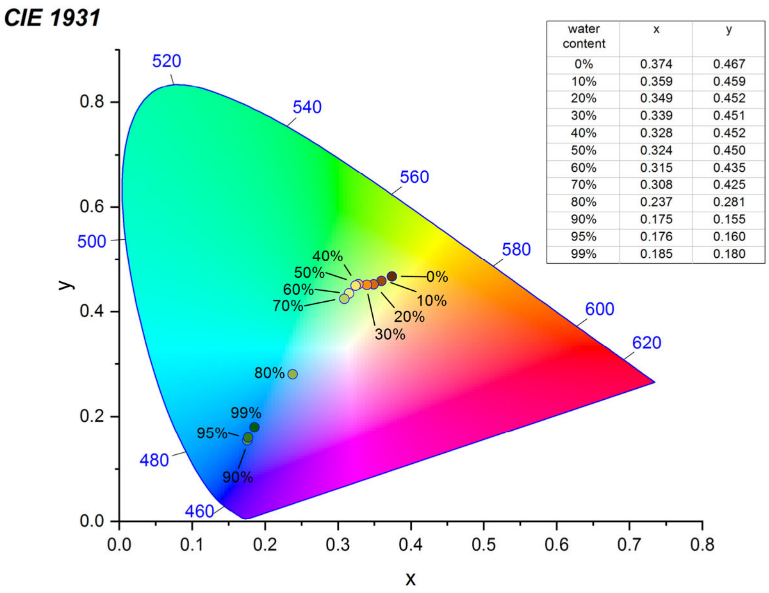

water content	x	y
0%	0.374	0.467
10%	0.359	0.459
20%	0.349	0.452
30%	0.339	0.451
40%	0.328	0.452
50%	0.324	0.450
60%	0.315	0.435
70%	0.308	0.425
80%	0.237	0.281
90%	0.175	0.155
95%	0.176	0.160
99%	0.185	0.180

Figure S14. CIE 1931 chromaticity plot with table of emission color coordinates.

4.3 Photophysical data of 1 and 2 at c = 100 µM

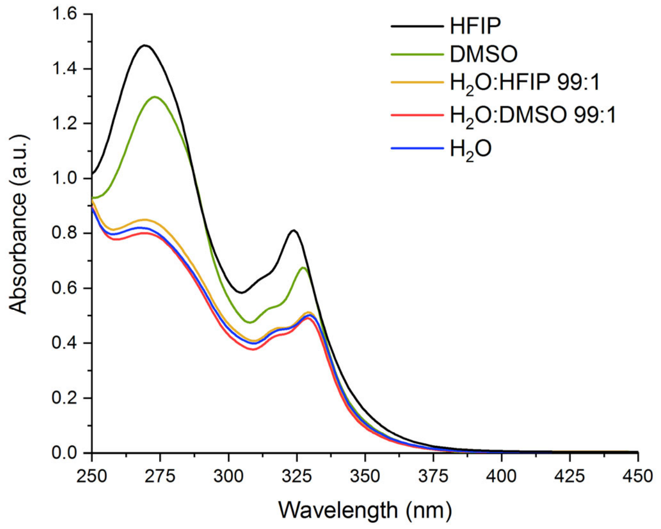

Figure S15. UV-Absorption spectra of **1** in different solvents at c = 100 µM.

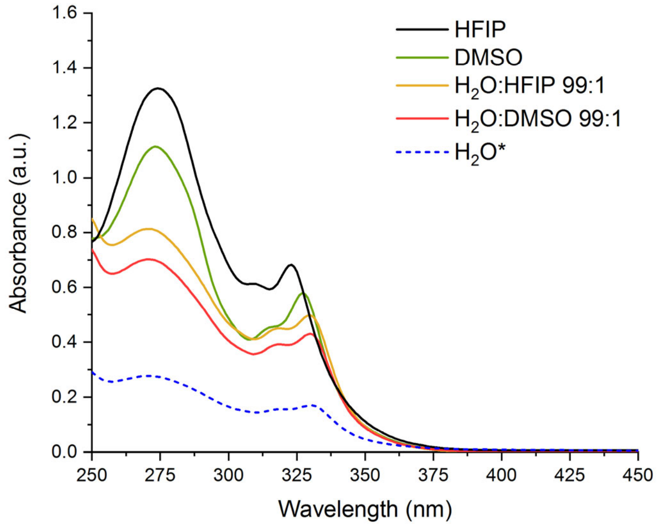

Figure S16. UV-Absorption spectra of **2** in different solvents at c = 100 µM. * Dotted line: **2** was not fully soluble in pure water at c = 100 µM. The saturated solution is estimated to be c ≈ 45 µM based on the absorption of the 10 µM solution, assuming the same absorption coefficient.

Table S1. Photophysical data for **1** and **2** in selected solvents and mixtures of the dilution series (c = 100 uM).

Compound	Solvent	λ_{abs} [nm]	$\lambda_{FL\ max}$ [nm][a]	$\Delta\tilde{\nu}$ [cm^{-1}]	Φ_{FL} [%][b]	τ_{avg} [ns][c]	k_r [$10^8\ s^{-1}$][d]	k_{nr} [$10^8\ s^{-1}$][d]
**1**	DMSO	327	542	12131	2.1	6.11	0.034	1.60
**1**	H_2O DMSO (99:1)	329	456	8465	3.6	8.42	0.043	1.14
**1**	HFIP	324	547	12583	≤ 0.1	-	-	-
**1**	H_2O HFIP (99:1)	329	449	8123	2.6	7.33	0.035	1.33
**1**	H_2O	330	464	8751	3.4	n.d.	-	-
**2**	DMSO	327	537	11959	3.1	7.95	0.039	1.22
**2**	H_2O DMSO (99:1)	330	452	8179	2.6	6.42	0.040	1.52
**2**	HFIP	323	545	12611	≤ 0.1	-	-	-
**2**	H_2O HFIP (99:1)	330	441	7627	2.0	5.53	0.036	1.77
**2**	H_2O	330	465	8798	2.6	n.d.	-	-

[a] Excitation at 365 nm. [b] The absolute error of the measurement was $\Delta\Phi$ = ± 0.1%. [c] Excitation at 300 nm. [d] k_r and k_{nr} were calculated using the equations $k_r = \Phi_F/\tau_{avg}$ and $k_{nr} = (1-\Phi_F)/\tau_{avg}$.

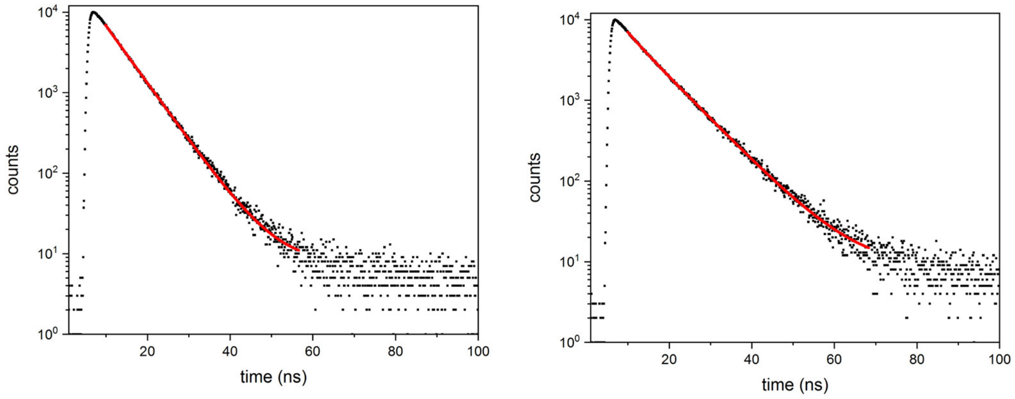

Figure S17. Fluorescence decay profiles of **1** (left) and **2** (right) in DMSO with exponential tail fits (red). Excitation at 300 nm; c = 100 µM. **1**: τ = 6.11 ± 0.01 ns; χ^2 = 1.067. **2**: τ_1 = 3.41 ± 0.47 ns (9.0 %); τ_2 = 8.40 ± 0.04 ns (91.0 %); τ_{avg} = 7.95 ± 0.07 ns; χ^2 = 1.136.

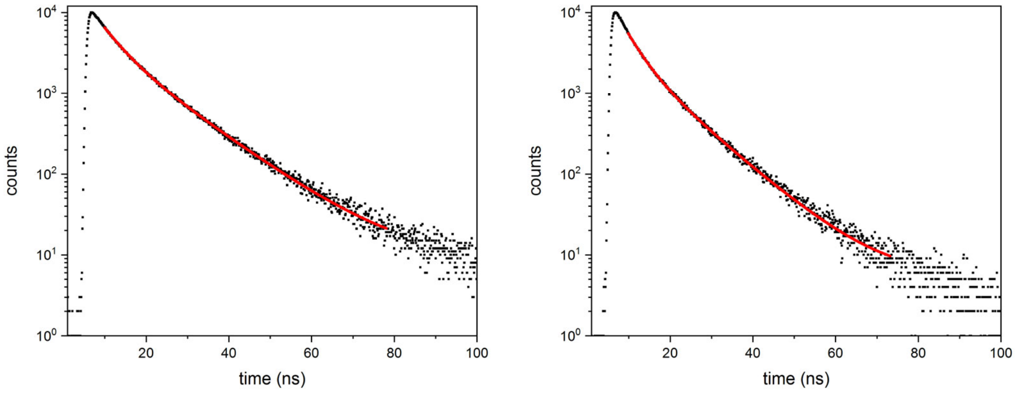

Figure S18. Fluorescence decay profiles of **1** (left) and **2** (right) in H_2O:DMSO (99:1) with exponential tail fits (red). Excitation at 300 nm; c = 100 µM. **1**: τ_1 = 4.70 ± 0.12 ns (49.9 %);

τ_2 = 12.1 ± 0.1 ns (50.1 %); τ_{avg} = 8.42 ± 0.13 ns; χ^2 = 1.048. **2**: τ_1 = 3.70 ± 0.06 ns (56.6 %); τ_2 = 9.96 ± 0.07 ns (43.4 %); τ_{avg} = 6.42 ± 0.07 ns; χ^2 = 0.997.

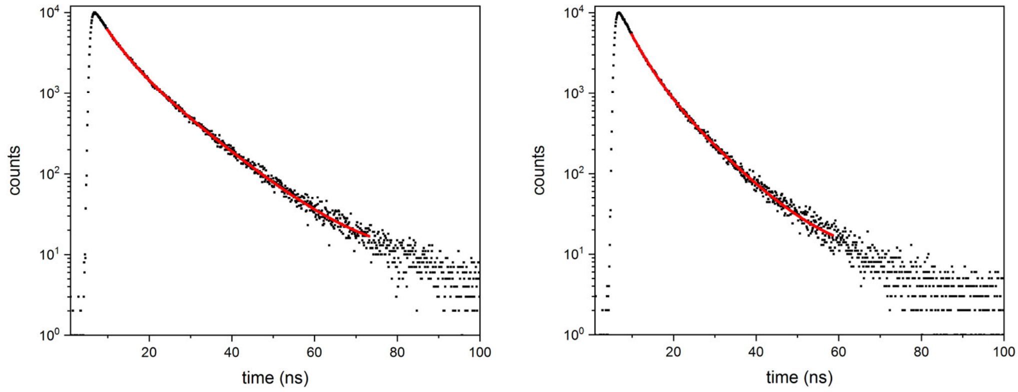

Figure S19. Fluorescence decay profiles of **1** (left) and **2** (right) in H_2O:HFIP (99:1) with exponential tail fits (red). Excitation at 300 nm; c = 100 µM. **1**: τ_1 = 4.15 ± 0.08 ns (51.0 %); τ_2 = 10.6 ± 0.1 ns (49.0 %); τ_{avg} = 7.33 ± 0.08 ns; χ^2 = 1.026. **2**: τ_1 = 3.63 ± 0.09 ns (63.6 %); τ_2 = 8.84 ± 0.20 ns (36.4 %); τ_{avg} = 5.53 ± 0.15 ns; χ^2 = 1.019.

5 Photophysical properties of 1 and 2 in solution at c = 10 µM

5.1 Photophysical properties of 1 in solution

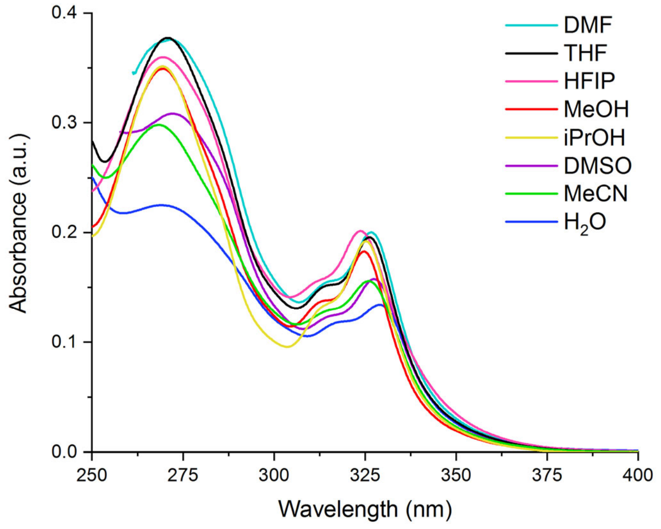

Figure S20. UV-absorption spectra of **1** in different solvents (c = 10 µM). Spectra with solvents DMF and DMSO are slightly cut off due to the too high absorption of the subtracted solvent background.

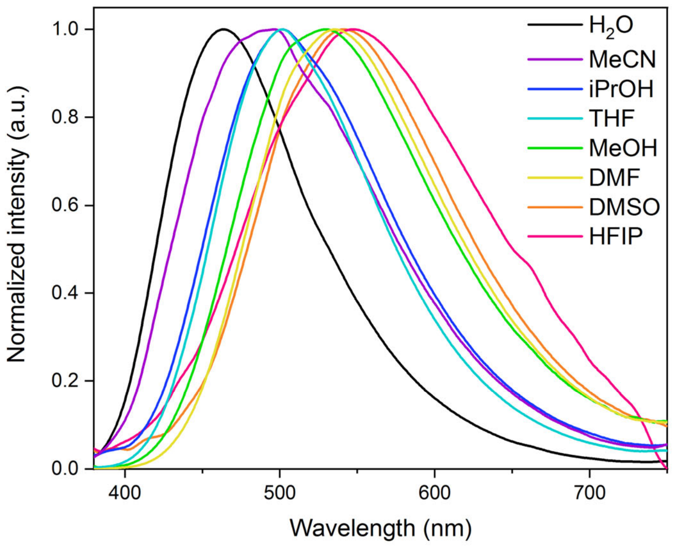

Figure S21. Normalized fluorescence emission spectra of **1** in different solvents (c = 10 uM). Excitation at 335 nm.

Table S2. Photophysical data for **1** in different solvents (c = 10 uM).

Solvent	λ_{abs} [nm]	$\lambda_{FL\ max}$ [nm][a]	$\Delta\tilde{\nu}$ [cm^{-1}]	Φ_{FL} [%][b]	τ_{avg} [ns][c]	k_r [10^8 s^{-1}][d]	k_{nr} [10^8 s^{-1}][d]
DMF	327	536	11924	2.4	6.41	0.037	1.52
DMSO	327	542	12131	2.1	6.05	0.035	1.62
H_2O	329	464	8843	3.4	7.81	0.044	1.24
HFIP	324	547	12583	≤ 0.1	-	-	-
iPrOH	325	502	10849	2.8	6.75	0.041	1.44
MeCN	326	497	10554	2.3	6.10	0.038	1.60
MeOH	325	529	11866	0.9	2.89	0.031	3.43
THF	326	502	10755	5.4	12.6	0.043	0.75

[a] Excitation at 335 nm. [b] The absolute error of the measurement was $\Delta\Phi = \pm 0.1\%$. [c] Excitation at 300 nm. [d] k_r and k_{nr} were calculated using the equations $k_r = \Phi_F/\tau_{avg}$ and $k_{nr} = (1-\Phi_F)/\tau_{avg}$.

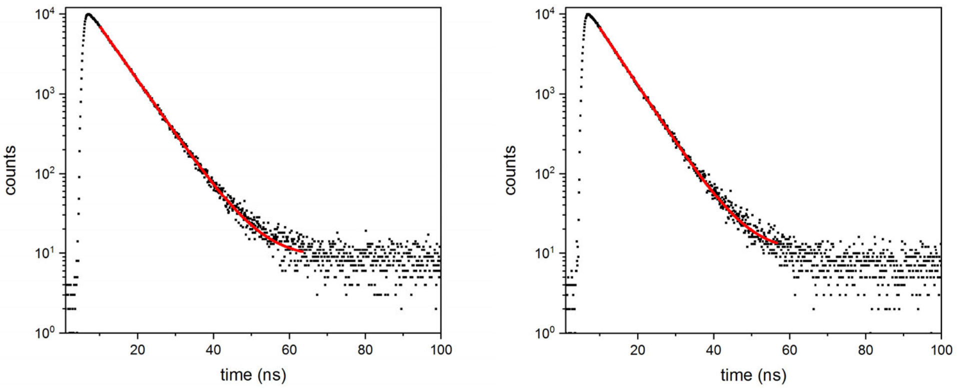

Figure S22. Fluorescence decay profiles of solutions of **1** in DMF (left) and DMSO (right) with exponential tail fits (red). Excitation at 300 nm; c = 10 μM. DMF: τ = 6.41 ± 0.01 ns; χ^2 = 0.974. DMSO: τ = 6.05 ± 0.01 ns; χ^2 = 1.068.

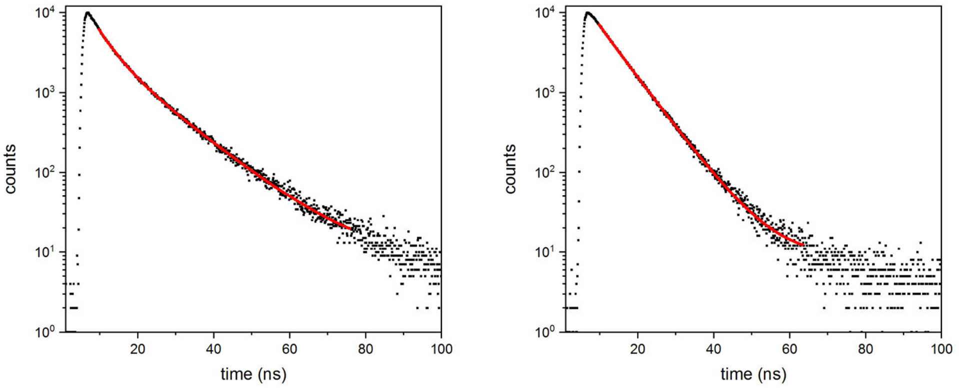

Figure S23. Fluorescence decay profiles of suspension of **1** in H_2O (left) and solution in 2-propanol (right) with exponential tail fits (red). Excitation at 300 nm; c = 10 μM. H_2O: τ_1 = 4.27 ± 0.09 ns (53.1 %); τ_2 = 11.8 ± 0.1 ns (46.9 %); τ_{avg} = 7.81 ± 0.12 ns; χ^2 = 1.033. 2-propanol: τ_1 = 4.29 ± 0.71 ns (13.4 %); τ_2 = 7.13 ± 0.09 ns (86.6 %); τ_{avg} = 6.75 ± 0.25 ns; χ^2 = 1.080.

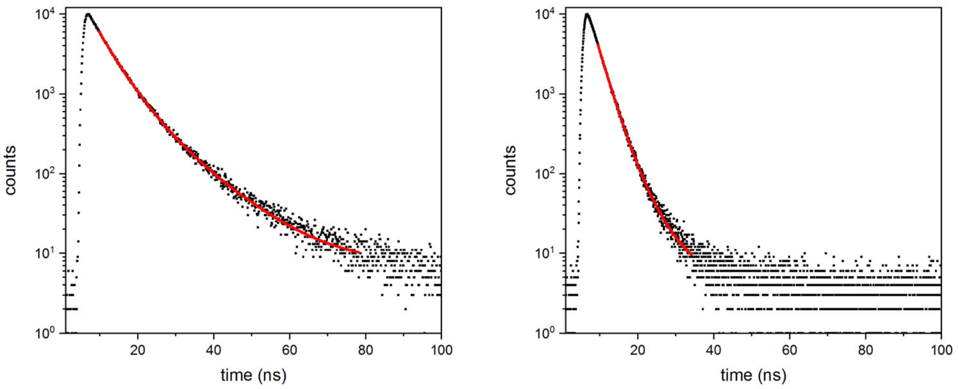

Figure S24. Fluorescence decay profiles of solutions of **1** in acetonitrile (left) and methanol (right) with exponential tail fits (red). Excitation at 300 nm; c = 10 μM. Acetonitrile: τ_1 = 4.75 ± 0.07 ns (79.1 %); τ_2 = 11.2 ± 0.3 ns (20.9 %); τ_{avg} = 6.10 ± 0.15 ns; χ^2 = 1.089.

Methanol: τ_1 = 2.62 ± 0.07 ns (91.7 %); τ_2 = 5.78 ± 0.95 ns (8.3 %); τ_{avg} = 2.89 ± 0.25 ns; χ^2 = 0.977.

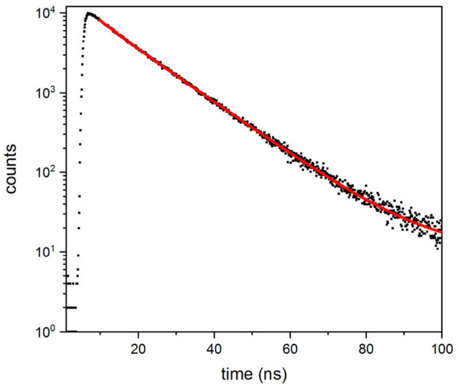

Figure S25. Fluorescence decay profile of a solution of **1** in THF with exponential tail fit (red). Excitation at 300 nm; c = 10 μM. τ_1 = 5.62 ± 0.98 ns (6.9 %); τ_2 = 13.1 ± 0.1 ns (93.1 %); τ_{avg} = 12.6 ± 0.1 ns; χ^2 = 0.989.

5.2 Photophysical properties of 2 in solution

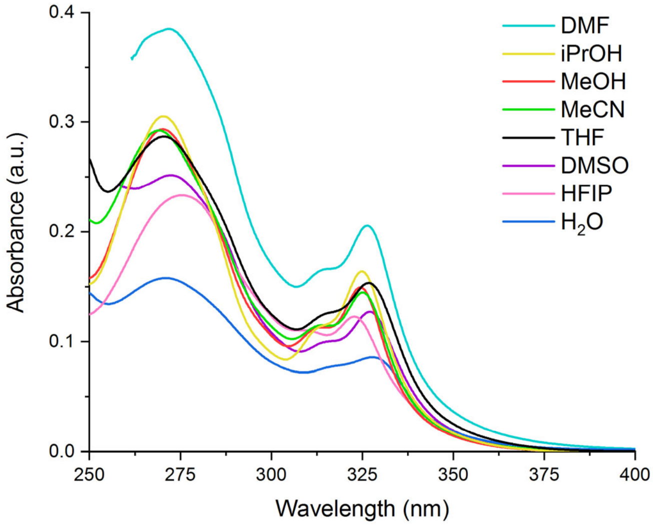

Figure S26. UV-absorption spectra of **2** in different solvents (c = 10 μM). Spectra with solvents DMF and DMSO are slightly cut off due to the too high absorption of the subtracted solvent background.

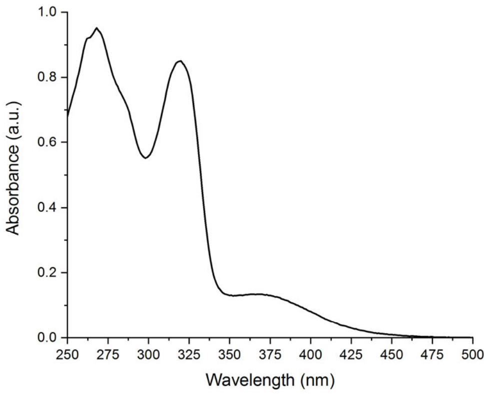

Figure S27. UV-vis absorption spectrum of **2** in DCM containing 1% trifluoroacetic acid (c = 10 µM).

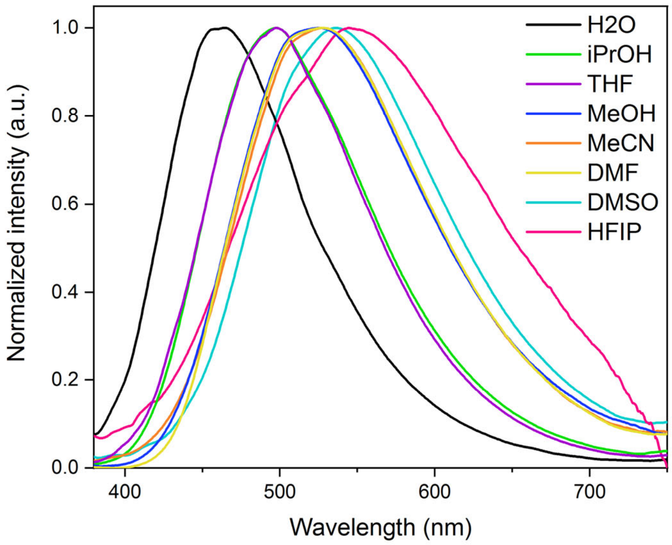

Figure S28. Normalized fluorescence emission spectra of **2** in different solvents (c = 10 uM; no fluorescence was detected in DCM containing 1% TFA). Excitation at 335 nm.

Table S3. Photophysical data for **2** in different solvents (c = 10 uM).

Solvent	λ_{abs} [nm]	$\lambda_{FL\ max}$ [nm][a]	$\Delta\tilde{\nu}$ [cm^{-1}]	Φ_{FL} [%][b]	τ_{avg} [ns][c]	k_r [10^8 s^{-1}][d]	k_{nr} [10^8 s^{-1}][d]
DMF	326	528	11735	2.6	8.23	0.032	1.18
DMSO	327	537	11959	3.1	8.23	0.038	1.18
H_2O	329	465	8890	2.6	6.57	0.040	1.48
HFIP	323	545	12611	≤ 0.1	-	-	-

iPrOH	325	498	10689	3.6	9.12	0.039	1.06
MeCN	325	528	11830	1.7	6.51	0.026	1.51
MeOH	324	525	11817	1.2	3.74	0.032	2.64
THF	327	498	10501	3.9	12.4	0.031	0.78

[a] Excitation at 335 nm. [b] The absolute error of the measurement was $\Delta\Phi$ = ± 0.1%. [c] Excitation at 300 nm. [d] k_r and k_{nr} were calculated using the equations $k_r = \Phi_F/\tau_{avg}$ and $k_{nr} = (1-\Phi_F)/\tau_{avg}$.

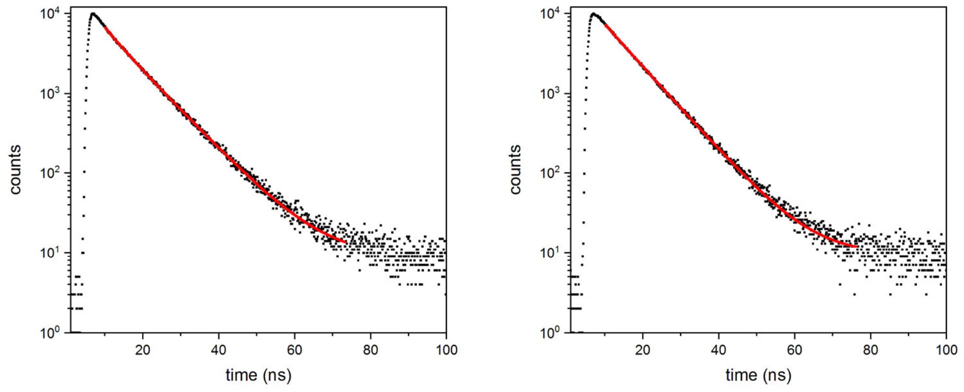

Figure S29. Fluorescence decay profiles of solutions of **2** in DMF (left) and DMSO (right) with exponential tail fits (red). Excitation at 300 nm; c = 10 µM. DMF: τ_1 = 4.35 ± 0.53 ns (15.2 %); τ_2 = 8.92 ± 0.10 ns (84.8 %); τ_{avg} = 8.23 ± 0.17 ns; χ^2 = 1.051. DMSO: τ = 8.23 ± 0.01 ns; χ^2 = 1.029.

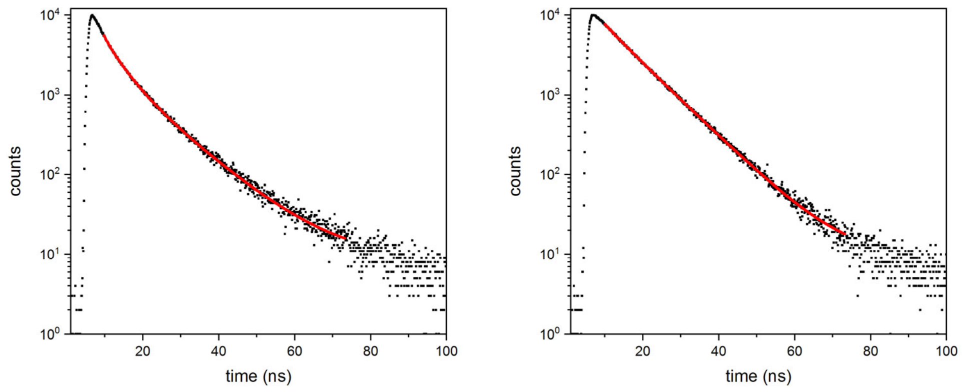

Figure S30. Fluorescence decay profiles of suspension of **2** in H_2O (left) and solution in 2-propanol (right) with exponential tail fits (red). Excitation at 300 nm; c = 10 µM. H_2O: τ_1 = 1.52 ± 0.45 ns (10.8 %); τ_2 = 4.65 ± 0.26 ns (54.9 %); τ_3 = 11.3 ± 0.2 ns (34.2 %); τ_{avg} = 6.57 ± 0.22 ns; χ^2 = 1.053. 2-propanol: τ_1 = 4.33 ± 0.77 ns (6.9 %); τ_2 = 9.47 ± 0.05 ns (93.1 %); τ_{avg} = 9.12 ± 0.10 ns; χ^2 = 1.017.

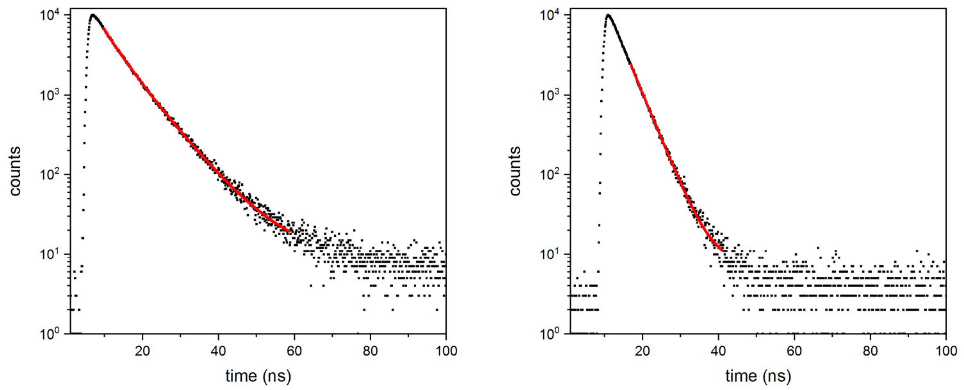

Figure S31. Fluorescence decay profiles of solutions of **2** in acetonitrile (left) and methanol (right) with exponential tail fits (red). Excitation at 300 nm; c = 10 μM. Acetonitrile: τ_1 = 4.73 ± 0.17 ns (50.4 %); τ_2 = 8.31 ± 0.15 ns (49.6 %); τ_{avg} = 6.51 ± 0.23 ns; χ^2 = 1.034. Methanol: τ = 3.74 ± 0.02 ns; χ^2 = 0.990.

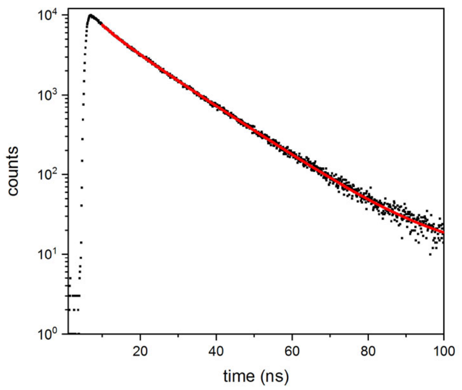

Figure S32. Fluorescence decay profile of a solution of **2** in THF with exponential tail fit (red). Excitation at 300 nm; c = 10 μM. τ_1 = 4.97 ± 0.26 ns (16.1 %); τ_2 = 13.8 ± 0.1 ns (83.9 %); τ_{avg} = 12.4 ± 0.1 ns; χ^2 = 1.041.

6 Gelation properties

6.1 Gelation experiments with 1.

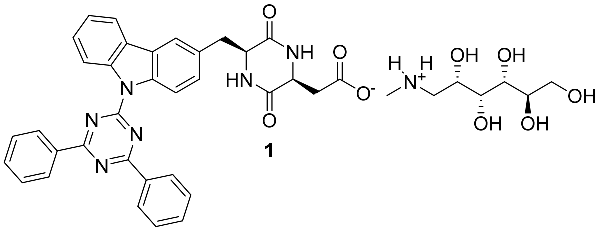

Figure S33. Structure of gelator **1**.

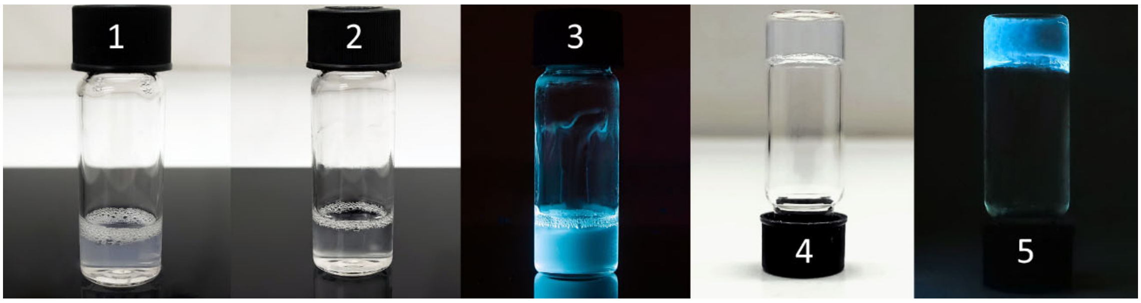

Figure S34. 1) Suspension of **1** in water (1 wt%) after vortexing; 2) sol of **1** in water (1 wt%) obtained from heating the suspension at 80 °C for 10 min; 3) the same sol under 365 nm UV-light irradiation; 4) gel of the same sol 17 h after addition of glucono-δ-lactone (GdL; 5.0 equiv); 5) the same gel under 365 nm UV-light irradiation.

Table S4. Gelation properties of **1** in water/organic solvent mixtures.[a]

Co-Solvent	1 h after addition	17 h after addition	At 80 °C[b]	1 h after heating	17 h after heating	Re-heating to 80 °C[b]	1 h after re-heating	1 d after re-heating
MeOH	Sol	Sol	S	Sol	Sol	S	Sol	Sol
2. EtOH	Sol	Sol	S	Sol	G	S	Sol	G
iPrOH	Sol	Sol	S	G	G	S	G	G
HFIP	P	G	G	G	G	G	G	G
Acetone	P	G	S	G	G	S	G	G
MeCN	P	G	Sol	G	G	Sol	G	G

DMSO	Sol	Sol	Sol	Sol	Sol	Sol	Sol	Sol
DMF	Sol	Sol	Sol	G	G	Sol	G	G
THF	S	S	S	S	S	S	S	S
1,4-Dioxane	G	G	G	G	G	G	G	G

[a] A co-solvent (500 µL) was added to a solution of **1** (10 mg) in H_2O (500 µL). S: Solution. G: Gel. P: Precipitate. [b] 10 minutes at 80 °C.

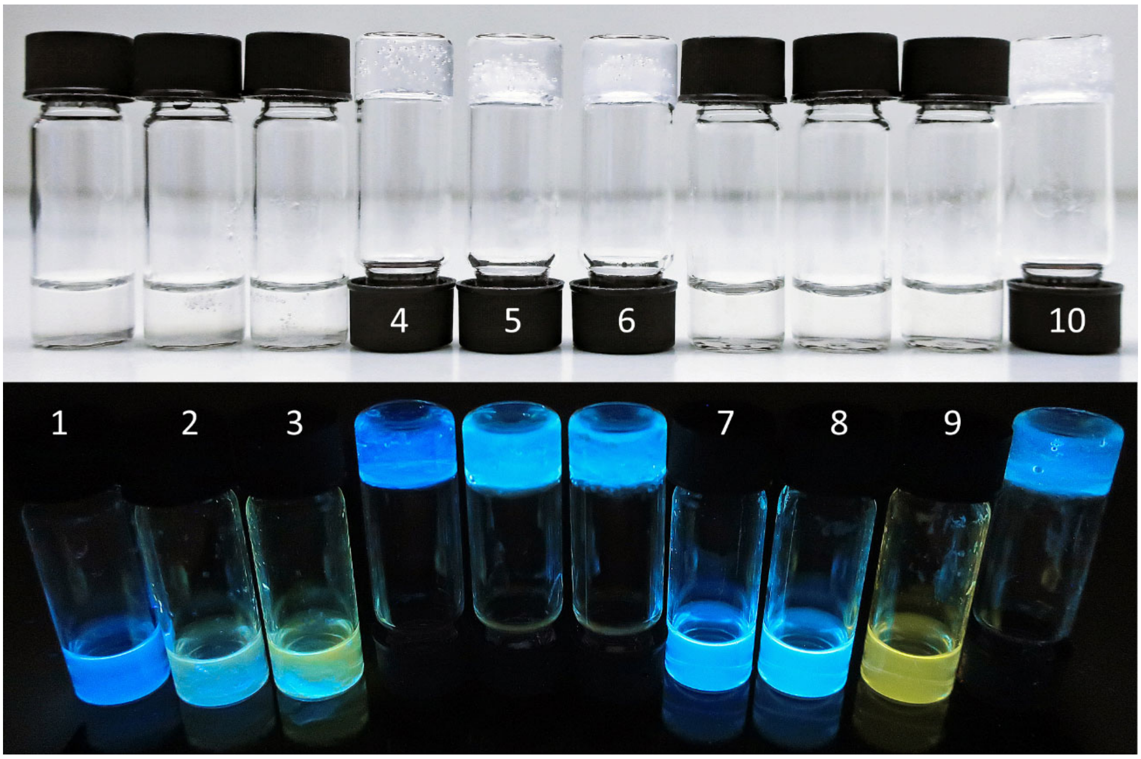

Figure S35. Water/organic solvent mixtures (1:1 v/v) of **1** (10 mg/mL) under ambient light (top) and under 365 nm UV-light irradiation (bottom), 17 h after addition of the co-solvent (see Table S4 for details). Co-solvents: 1) MeOH; 2) EtOH; 3) *i*-PrOH; 4) HFIP; 5) acetone; 6) acetonitrile; 7) DMSO; 8) DMF; 9) THF; 10) 1,4-dioxane.

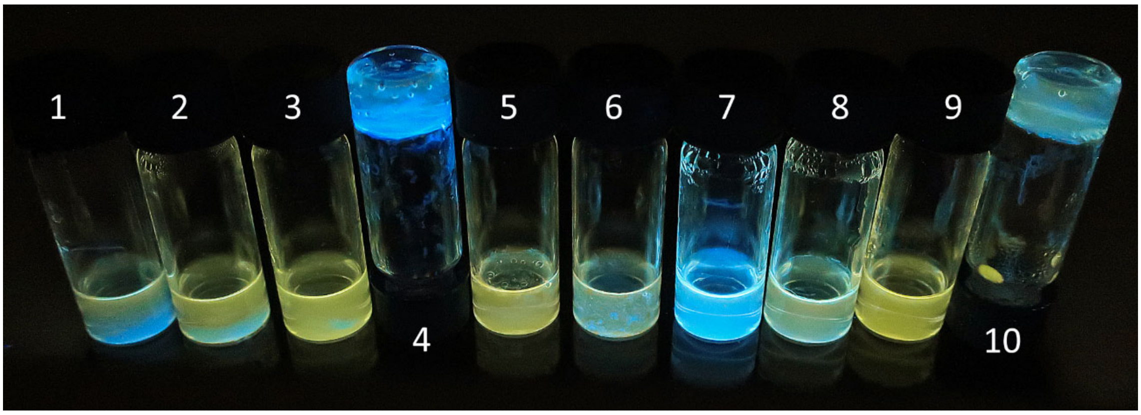

Figure S36. Water/organic solvent mixtures (1:1 v/v) of **1** (10 mg/mL) under 365 nm UV-light irradiation, 20 - 100 s after heating to 80 °C for 10 min (see Table S4 for details). Co-solvents: 1) MeOH; 2) EtOH; 3) *i*-PrOH; 4) HFIP; 5) acetone; 6) acetonitrile; 7) DMSO; 8) DMF; 9) THF; 10) 1,4-dioxane.

The blue fluorescence of mixtures 1, 2 and 3 in Fig. S36 was not present after removing the heat-source and immediately irradiating the mixtures. It is an effect of the aggregation happening upon cooling down due to the delay between the removal of the heat source and taking the picture.

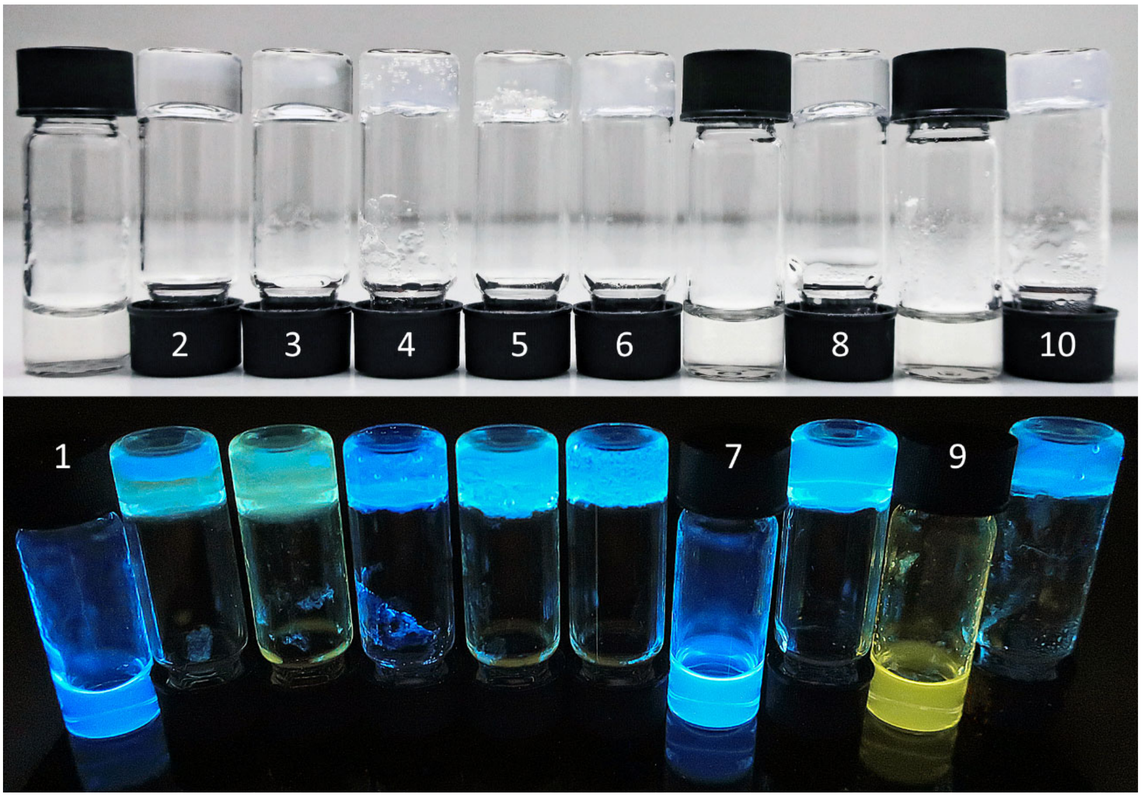

Figure S37. Water/organic solvent mixtures (1:1 v/v) of **1** (10 mg/mL) under ambient light (top) and under 365 nm UV-light irradiation (bottom), 24 h after heating to 80 °C (see Table S4 for

details). Co-solvents: 1) MeOH; 2) EtOH; 3) *i*-PrOH; 4) HFIP; 5) acetone; 6) acetonitrile; 7) DMSO; 8) DMF; 9) THF; 10) 1,4-dioxane.

Table S5. Gelation properties of **1** with meglumine in water/organic solvent mixtures.[a]

Co-Solvent	1 h after addition	17 h after addition	At 80 °C[b]	1 h after heating	17 h after heating	Re-heating to 80 °C[b]	1 h after reheating	1 d after reheating
MeOH	Sol	Sol	S	G	G	S	G	G
EtOH	P	Sol	S	G	G	S	G	G
iPrOH	P	Sol	Sol[c]	G	G	S	G	G
HFIP	P	G	P	P	G+S	S+P	P	G+P
Acetone	P	G	S	G	G	S	G	G
MeCN	P	G	S	G	G	S	G	G
DMSO	Sol	Sol	S	G	G	S	G	G
DMF	Sol	Sol	S	G	G	S	G	G
THF	S	S	S	S	S	S	S	S
1,4-Dioxane	G	G	S	G	G	S	G	G

[a] A co-solvent (500 µL) was added to a solution of **1** (10 mg) and meglumine (10 mg) in H_2O (500 µL). S: Solution. G: Gel. P: Precipitate. [b] 10 minutes at 80 °C. [c] Fluorescence indicated a solution, yet the sol was slightly turbid.

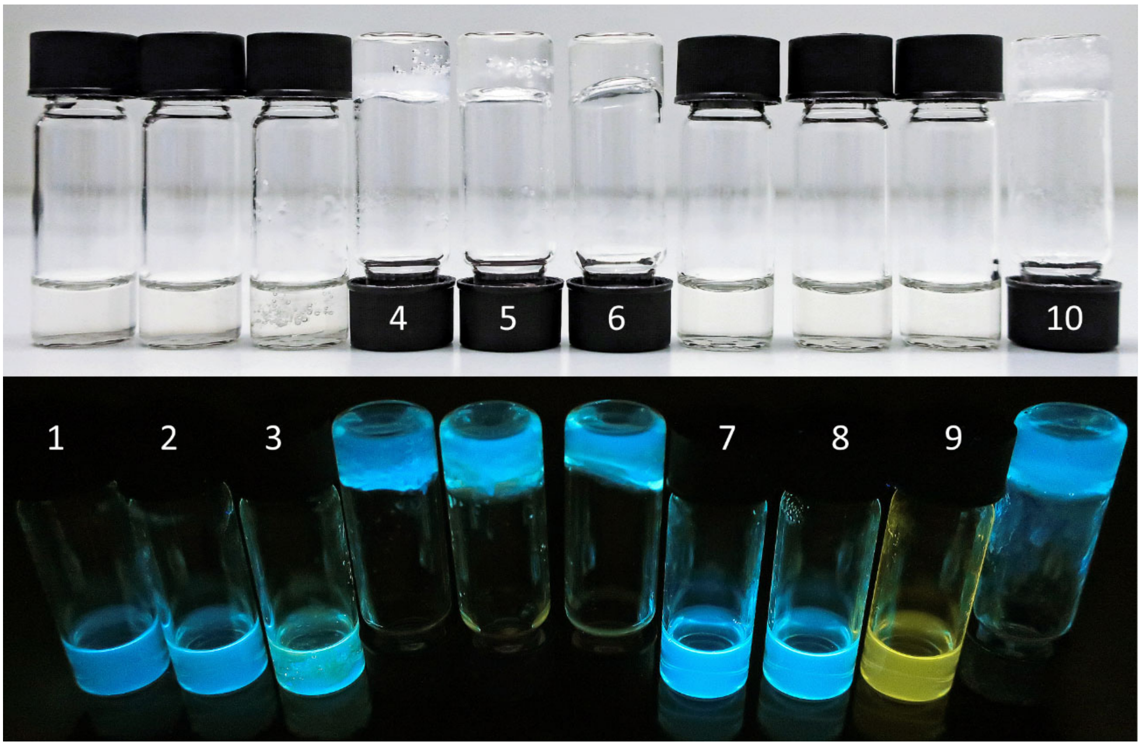

Figure S38. Water/organic solvent mixtures (1:1 v/v) of **1** + meglumine (both 10 mg/mL) under ambient light (top) and under 365 nm UV-light irradiation (bottom), 17 h after addition of the co-solvent (see Table S5 for details). Co-solvents: 1) MeOH; 2) EtOH; 3) *i*-PrOH; 4) HFIP; 5) acetone; 6) acetonitrile; 7) DMSO; 8) DMF; 9) THF; 10) 1,4-dioxane.

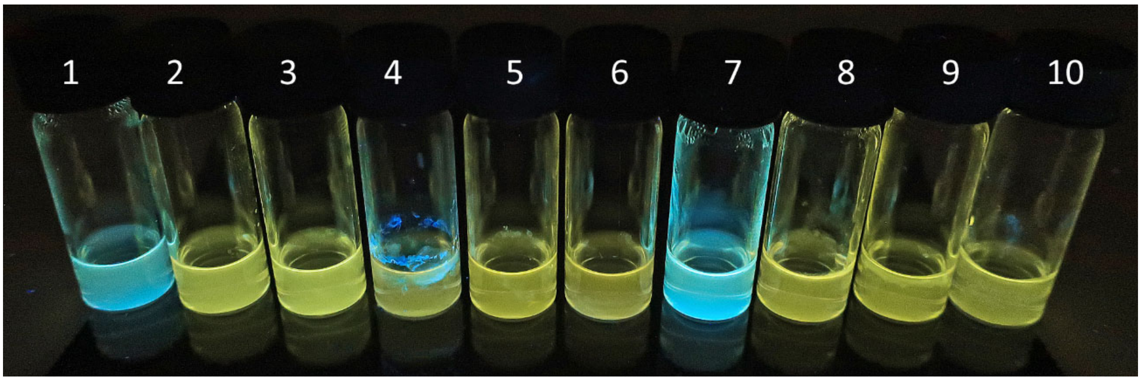

Figure S39. Water/organic solvent mixtures (1:1 v/v) of **1** + meglumine (both 10 mg/mL) under 365 nm UV-light irradiation, 20 - 100 s after heating to 80 °C for 10 min (see Table S5 for details). Co-solvents: 1) MeOH; 2) EtOH; 3) *i*-PrOH; 4) HFIP; 5) acetone; 6) acetonitrile; 7) DMSO; 8) DMF; 9) THF; 10) 1,4-dioxane.

The blue fluorescence of mixtures 1 and 7 in Fig. S39 was not present after removing the heat-source and immediately irradiating the mixtures. It is an effect of the aggregation happening upon cooling down due to the delay between the removal of the heat source and taking the picture.

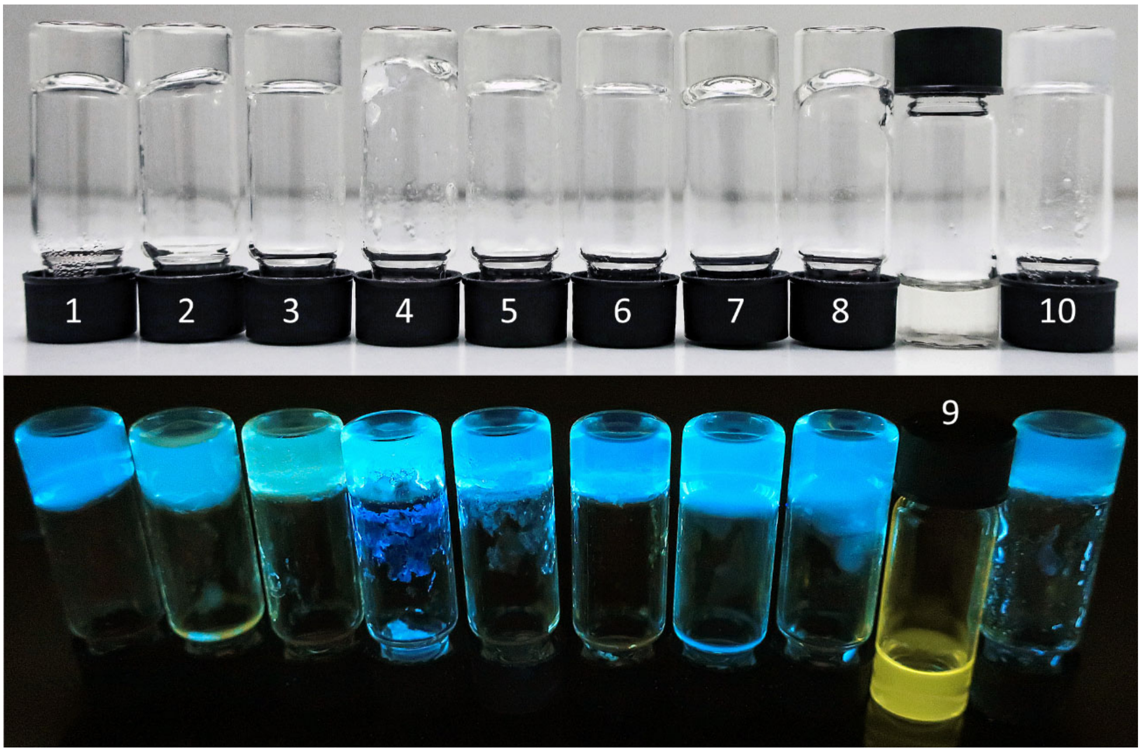

Figure S40. Water/organic solvent mixtures (1:1 v/v) of **1** + meglumine (both 10 mg/mL) under ambient light (top) and under 365 nm UV-light irradiation (bottom), 24 h after heating to 80 °C (see Table S5 for details). Co-solvents: 1) MeOH; 2) EtOH; 3) *i*-PrOH; 4) HFIP; 5) acetone; 6) acetonitrile; 7) DMSO; 8) DMF; 9) THF; 10) 1,4-dioxane.

6.2 Gelation experiments with 2.

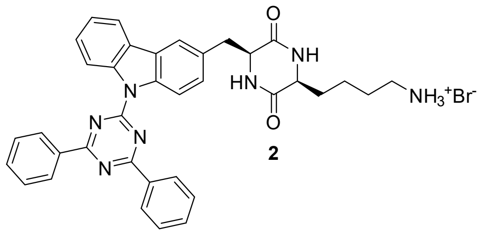

2

Figure S41. Structure of gelator **2**.

Gelation by co-solvents. A co-solvent (500 µL) was added to a solution of **2** (10 mg) and TFA (15 µL) in HFIP (500 µL). Co-solvents added from left to right in figures **S1** and **S2**: 1) H_2O, 2) MeOH, 3) *i*-PrOH, 4) acetone, 5) CH_3CN, 6) DMF, 7) DMSO, 8) THF, 9) 1,4-dioxane, 10) ethyl acetate, 11) toluene, 12) DCM.

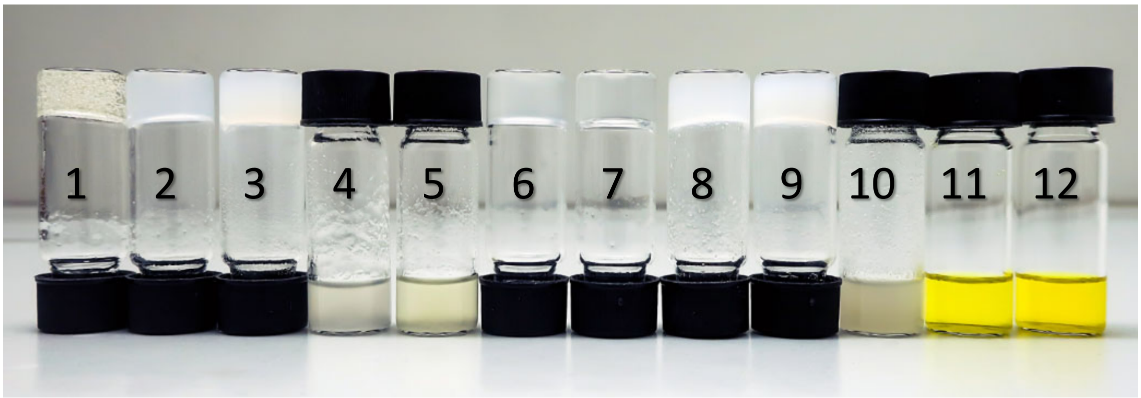

Figure S42. Mixed HFIP/co-solvent gels of **2**, 1 h after addition of the co-solvent. See description above for details

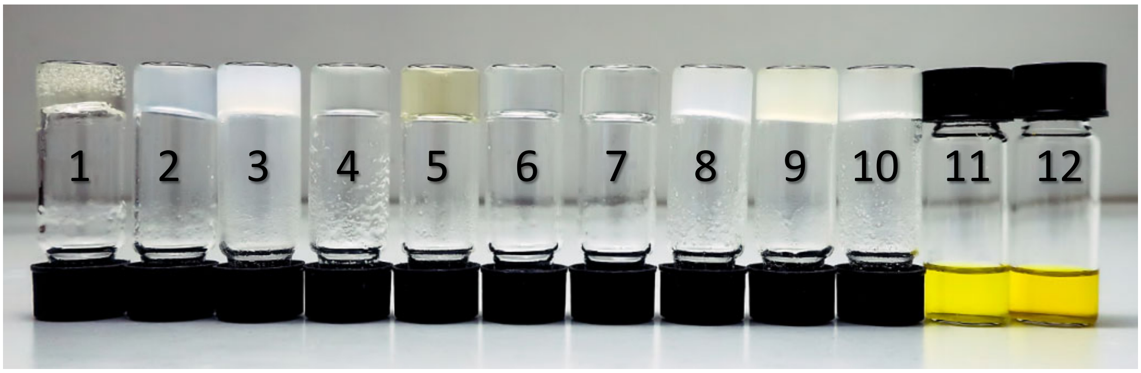

Figure S43. Mixed HFIP/co-solvent gels of **2**, 3 days after addition of the co-solvent. See description above for details.

Minimum water fraction needed to be added to induce gelation of **2** (0.5 wt%) and TFA (5 µL) in HFIP.

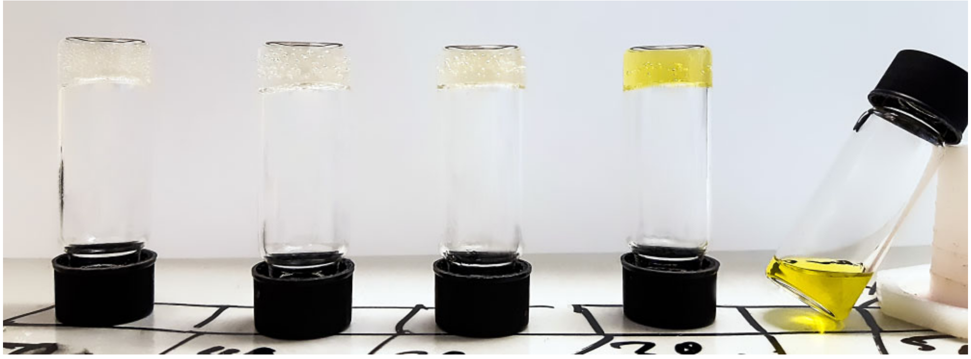

Figure S44. Gels of **2** from HFIP/TFA mixtures at 0.5 wt% with different water fractions, 24 h after the addition of water. From left to right: (50, 40, 30, 20, 10) vol% H_2O.

Table S6. Calculation of solvent fractions for water/HFIP gelation experiments with **2**.

Vol% H_2O	**2** /mg	TFA /mg	H_2O /mg (≙/µL)	HFIP /mg	HFIP /µL	mass sum /mg	**2** /wt%	Total volume /µL
10%	5	7.5	64	923	577	1000	0.50	646
20%	5	7.5	134	854	534	1000	0.50	673
30%	5	7.5	209	779	487	1000	0.50	701
40%	5	7.5	291	698	436	1001	0.50	732
50%	5	7.5	380	608	380	1001	0.50	765

7 Fluorescence spectra and life-times

7.1 Fluorescence spectra and life-times of solids

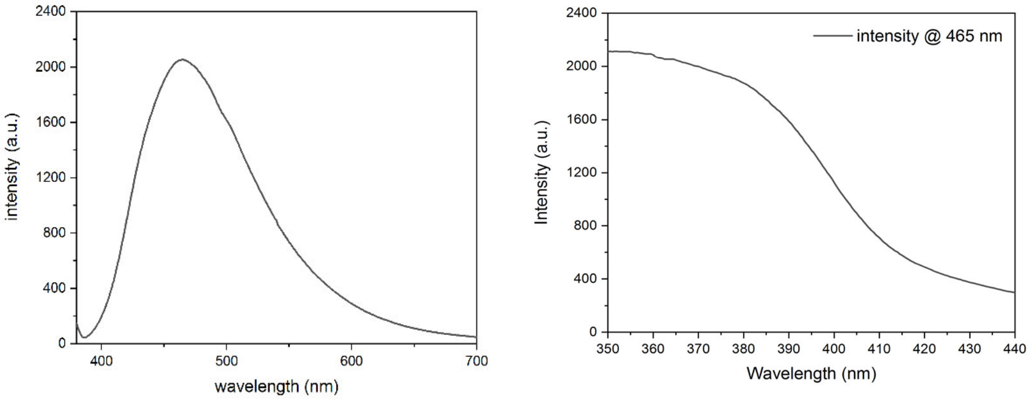

Figure S45. Fluorescence spectrum of solid **1** (excitation at 365 nm, left) and excitation spectrum (right). The absolute quantum yield of the fluorescence was determined to be Φ_F = 0.039 using an integrating sphere.

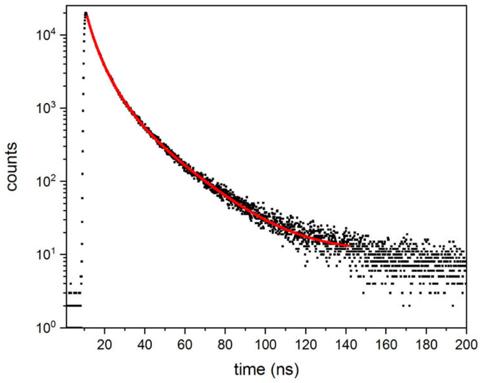

Figure S46. Fluorescence decay profile with exponential tail fit (red) (excitation at 373 nm). τ_1 = 2.88 ± 0.1 ns (43.9%); τ_2 = 6.68 ± 0.2 ns (46.0%); τ_3 = 19.4 ± 0.3 ns (10.1%); τ_{avg} = 6.30 ± 0.16 ns; χ^2 = 1.007.

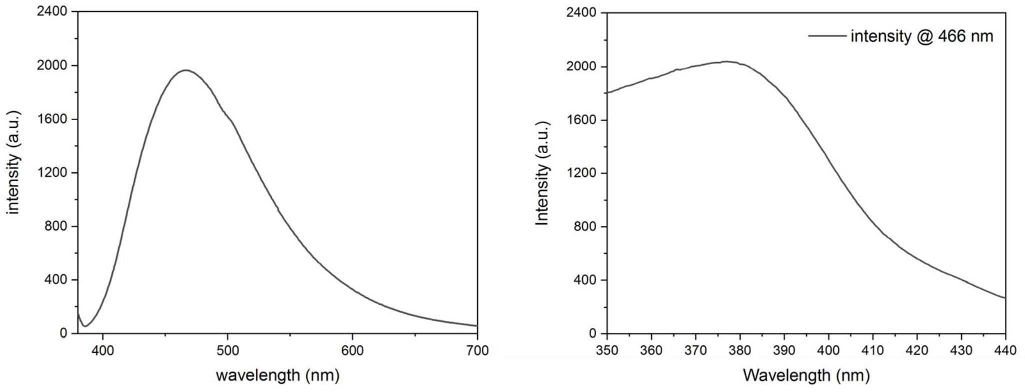

Figure S47. Fluorescence spectrum of solid **2** (excitation at 365 nm, left) and excitation spectrum (right). The absolute quantum yield of the fluorescence was determined to be $\Phi_F = 0.038$ using an integrating sphere.

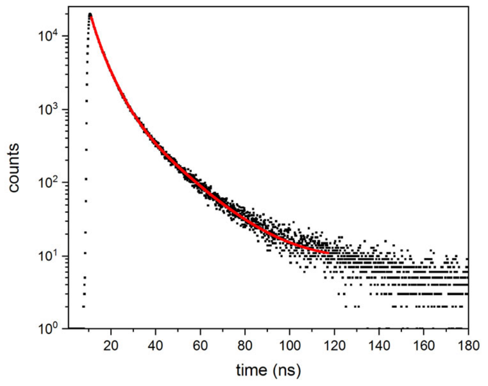

Figure S48. Fluorescence decay profile with exponential tail fit (red) (excitation at 373 nm). τ_1 = 2.6 ± 0.1 ns (37.7%); τ_2 = 6.01 ± 0.14 ns (55.6%); τ_3 = 17.9 ± 0.3 ns (6.8%); τ_{avg} = 5.54 ± 0.13 ns; χ^2 = 1.083.

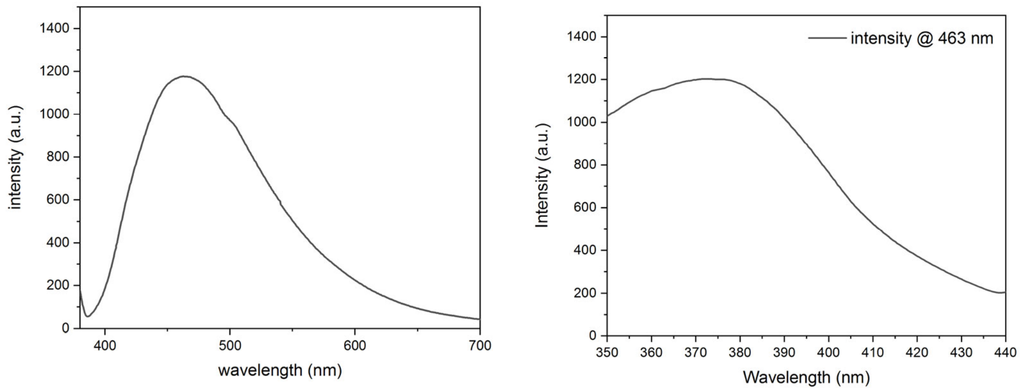

Figure S49. Fluorescence spectrum of solid **7** (excitation at 365 nm, left) and excitation scan (right). The absolute quantum yield of the fluorescence was determined to be $\Phi_F = 0.030$ using an integrating sphere.

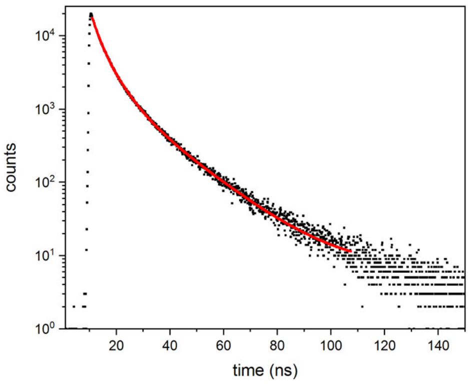

Figure S50. Fluorescence decay profile with exponential tail fit (red) (excitation at 373 nm). τ_1 = 2.56 ± 0.08 ns (49.1%); τ_2 = 6.26 ± 0.23 ns (41.3%); τ_3 = 16.8 ± 0.4 ns (9.6%); τ_{avg} = 5.46 ± 0.17 ns; χ^2 = 1.165.

7.2 Fluorescence spectra and life-times of sols, gels and solutions

1, dissolved in H_2O/THF

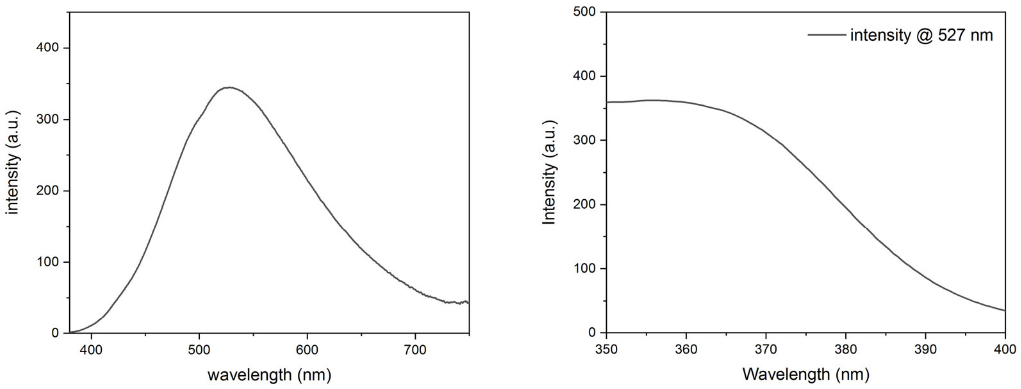

Figure S51. Fluorescence emission spectrum of **1** (6.0 mg/mL) in a 3/2 water/THF mixture (excitation at 365 nm, left) and excitation spectrum (right). The absolute quantum yield of the fluorescence was determined to be Φ_F = 0.012 using an integrating sphere.

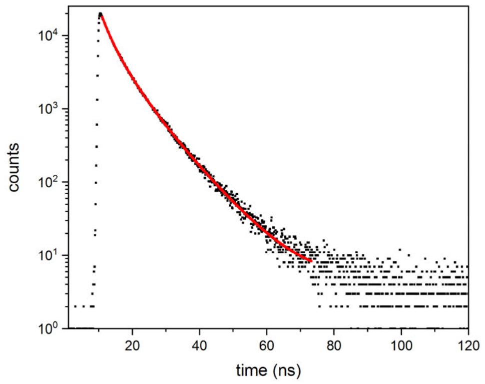

Figure S52. Fluorescence decay profile of **1** (6.0 mg/mL) in a 3/2 water/THF mixture with exponential tail fit (red) (excitation at 373 nm). τ_1 = 1.97 ± 0.19 ns (29.8%); τ_2 = 4.44 ± 0.39 ns (47.9%); τ_3 = 8.8 ± 0.3 ns (22.3%); τ_{avg} = 4.67 ± 0.35 ns; χ^2 = 1.053.

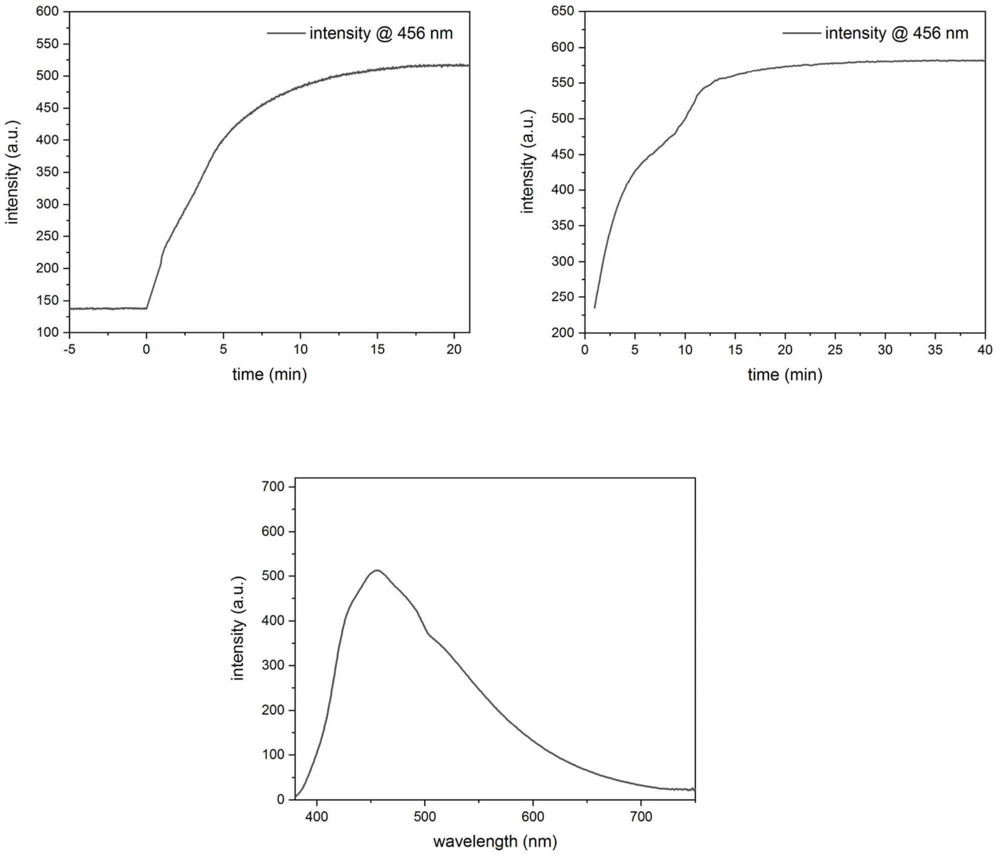

Figure S53. Upper: Intensity of the AIBSE-maximum at 456 nm (excitation at 365 nm) over time after addition of glucono-δ-lactone (12 equiv) of a solution of **1** (6.0 mg/mL) in 60% H_2O and 40% THF (left), or a solution of **1** (6.7 mg/mL) in 67% H_2O and 33% THF (right). Lower: Fluorescence emission spectrum of the resulting gel after 1 h (excitation at 365 nm).

1, H_2O sol

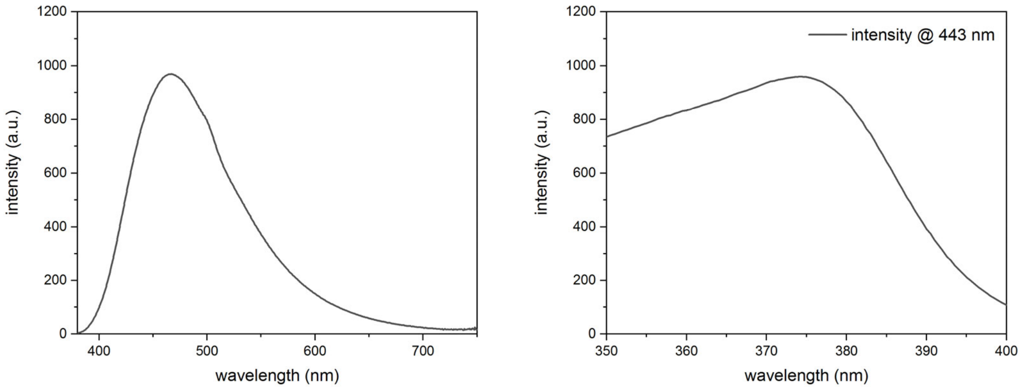

Figure S54. Fluorescence emission spectrum of the sol of **1** in water (1 wt%) after heating to 80 °C for 10 min and cooling to rt (excitation at 365 nm, left) and excitation spectrum (right). The absolute quantum yield of the fluorescence was determined to be Φ_F = 0.035 using an integrating sphere.

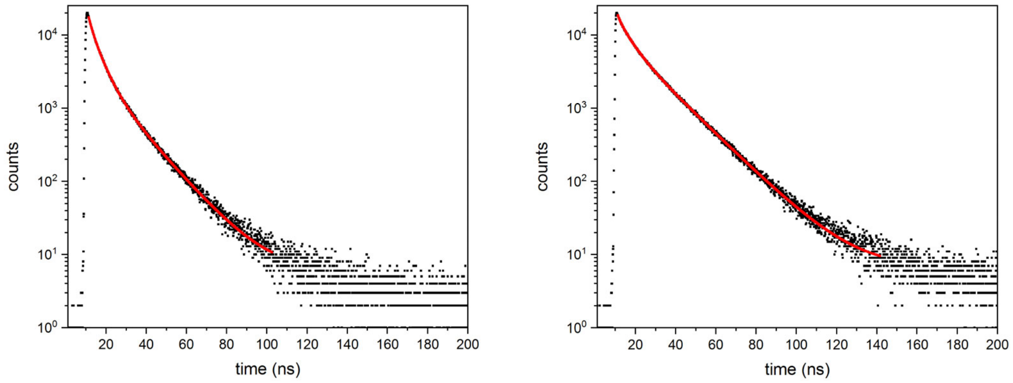

Figure S55. Fluorescence decay profiles with exponential tail fits (red) of the above sol under air (left), and deaerated under a nitrogen atmosphere (right) (excitation at 373 nm). Aerated sol: τ_1 = 1.9 ± 0.16 ns (21.3%); τ_2 = 4.93 ± 0.13 ns (60.7%); τ_3 = 14.1 ± 0.2 ns (18.0%); τ_{avg} = 5.93 ± 0.13 ns; χ^2 = 1.026. Deaerated sol: τ_1 = 1.33 ± 0.17 ns (10.2%); τ_2 = 5.79 ± 0.13 ns (43.1%); τ_3 = 16.3 ± 0.1 ns (46.6%); τ_{avg} = 10.25 ± 0.08 ns; χ^2 = 1.054.

1, gluconolactone (GdL) gel from H_2O

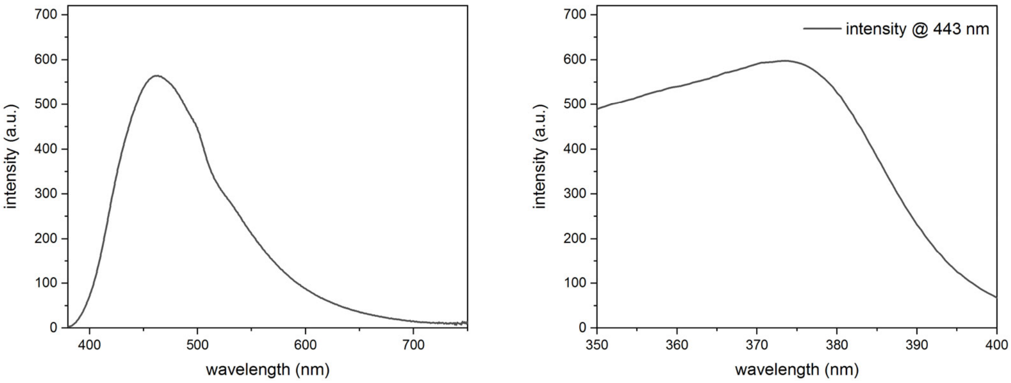

Figure S56. Fluorescence emission spectrum of a gel of **1** in water (1 wt%; gelation by 3.0 equiv gluconolactone), 17 h after gel formation (excitation at 365 nm, left) and excitation spectrum (right). The (normalized) spectra of the 4 weeks old gel are identical to the spectra shown above. The absolute quantum yield of the fluorescence was determined to be Φ_F = 0.025 using an integrating sphere.

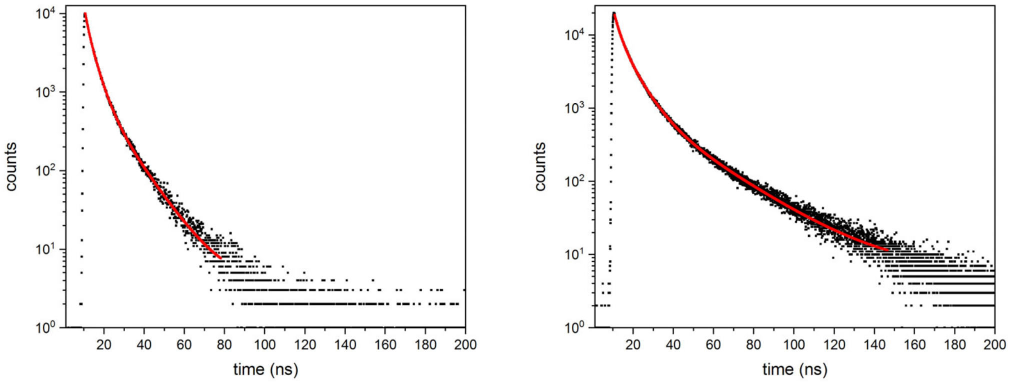

Figure S57. Fluorescence decay profiles of the above gel with exponential tail fits (red), 17 h after gelation (left), and four weeks after gelation (right) (excitation at 373 nm). 17 h old gel: τ_1 = 1.44 ± 0.16 ns (21.8%); τ_2 = 3.98 ± 0.12 ns (66.2%); τ_3 = 11.8 ± 0.3 ns (12.0%); τ_{avg} = 4.37 ± 0.14 ns; χ^2 = 1.051. Four weeks old gel:. τ_1 = 2.62 ± 0.05 ns (43.1%); τ_2 = 7.5 ± 0.1 ns (49.8%); τ_3 = 24.5 ± 0.3 ns (7.1%); τ_{avg} = 6.61 ± 0.08 ns; χ^2 = 1.097.

1, gel from H_2O/MeCN + meglumine

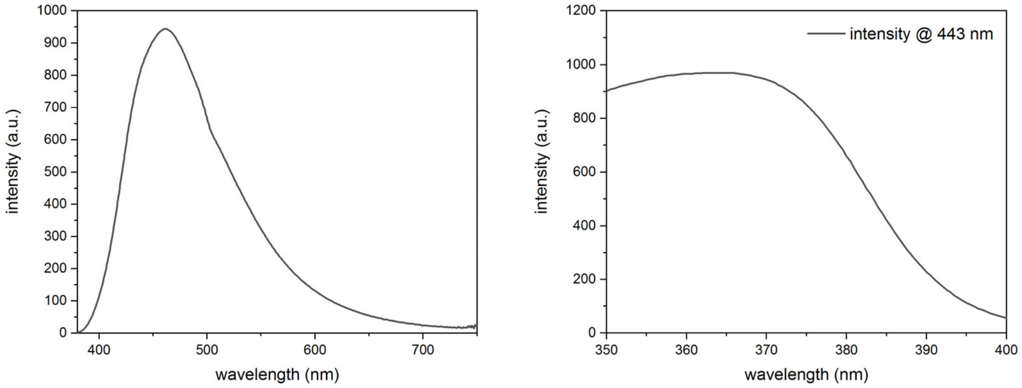

Figure S58. Fluorescence emission spectrum of a gel of **1** (10 mg/mL) from water:acetonitrile (1:1, with 10 mg/mL meglumine), 17 h after gel formation (excitation at 365 nm, left) and excitation spectrum (right). The absolute quantum yield of the fluorescence was determined to be Φ_F = 0.027 using an integrating sphere.

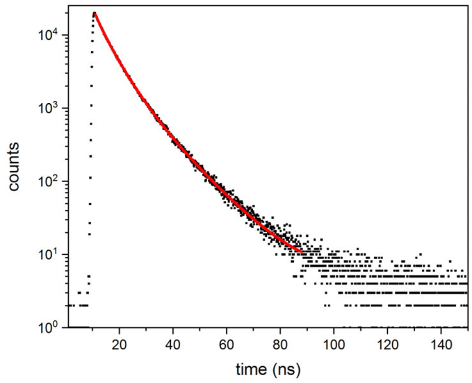

Figure S59. Fluorescence decay profile of the above gel with exponential tail fit (red) (17 h after gelation) (excitation at 373 nm). τ_1 = 2.01 ± 0.32 ns (10.9%); τ_2 = 5.6 ± 0.2 ns (69.9%); τ_3 = 12.1 ± 0.3 ns (19.2%); τ_{avg} = 6.45 ± 0.24 ns; χ^2 = 1.036.

2, HFIP/H_2O gel

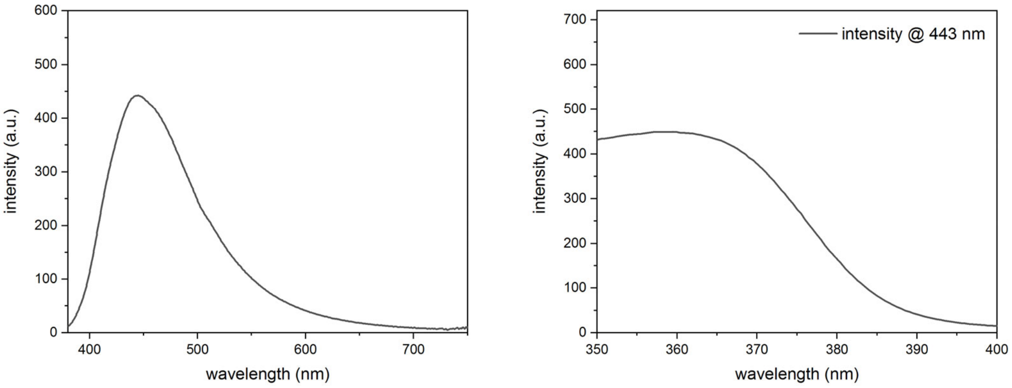

Figure S60. Fluorescence emission spectrum of a gel of **2** (10 mg/mL) from a 1:1 water/HFIP mixture containing 1% TFA (excitation at 365 nm, left) and excitation spectrum (right). The absolute quantum yield of the fluorescence was determined to be Φ_F = 0.019 using an integrating sphere.

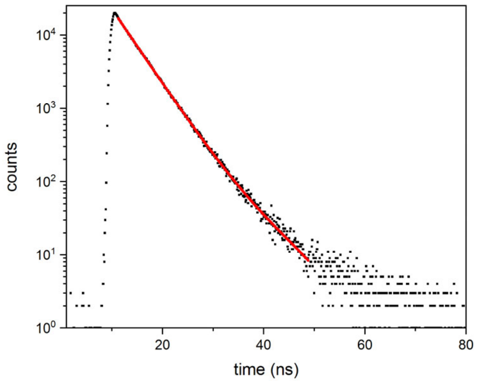

Figure S61. Fluorescence decay profile of the above gel with exponential tail fit (red) (excitation at 373 nm). τ_1 = 4.26 ± 0.02 ns (99.2%); τ_2 = 17.63 ± 10.06 ns (0.8%); τ_{avg} = 4.37 ± 0.11 ns; χ^2 = 1.070.

7.3 Temperature dependent fluorescence emission of gels of 1

1, gel from H_2O/MeCN + meglumine

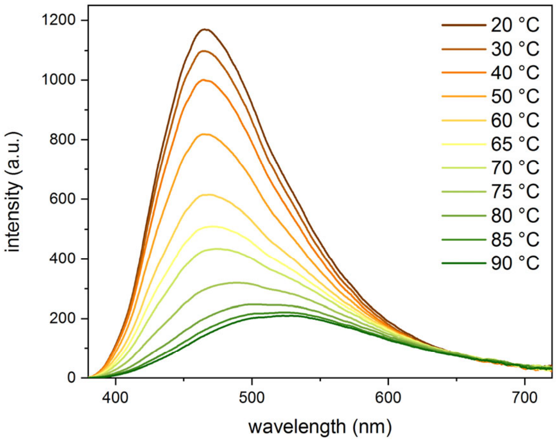

Figure S62. Temperature dependent fluorescence emission spectra of the gel of **1** (10 mg/mL) from water:acetonitrile (1:1, with 10 mg/mL meglumine) while heating (excitation at 365 nm). The gel had aged for 3 days prior to the measurement.

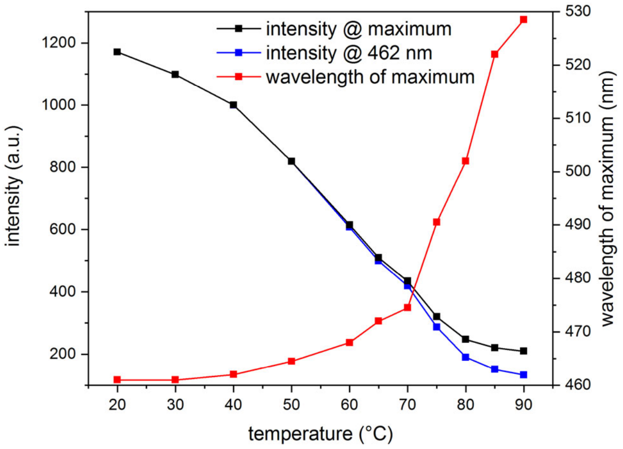

Figure S63. Temperature dependence of the maximum, the aggregation-maximum (462 nm) and the wavelength of the maximum of the gel above (excitation at 365 nm).

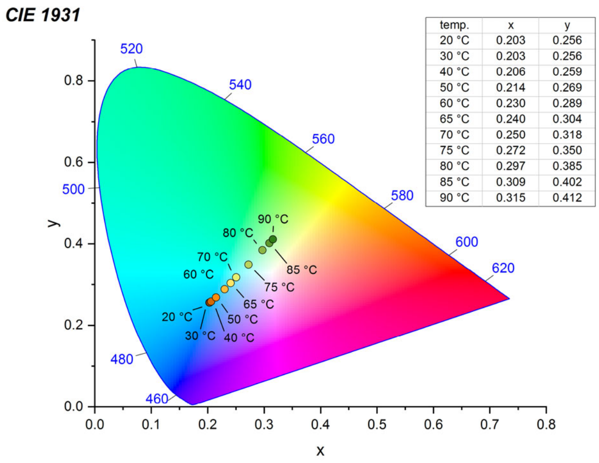

temp.	x	y
20 °C	0.203	0.256
30 °C	0.203	0.256
40 °C	0.206	0.259
50 °C	0.214	0.269
60 °C	0.230	0.289
65 °C	0.240	0.304
70 °C	0.250	0.318
75 °C	0.272	0.350
80 °C	0.297	0.385
85 °C	0.309	0.402
90 °C	0.315	0.412

Figure S64. CIE 1931 chromaticity plot of the temperature dependent fluorescence emission spectra above with table of emission color coordinates.

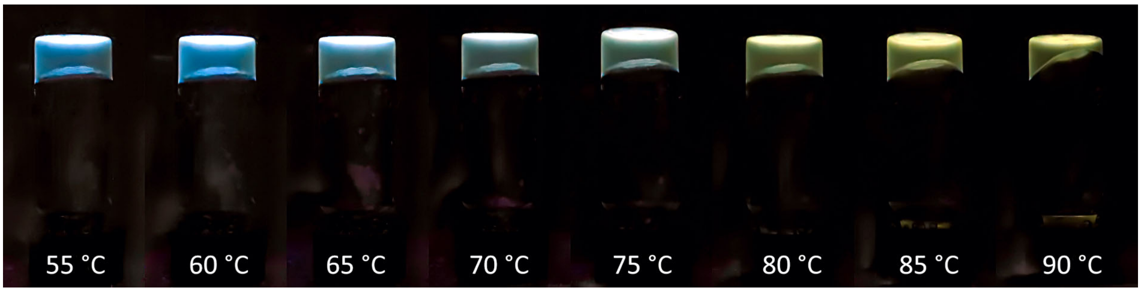

Figure S65. Fluorescence (excitation by a 365 nm UV lamp) of the H_2O/MeCN + meglumine gel of **1** at different temperatures while heating, showing a noticeable change in fluorescence before the gel melts. After more than 10 min at 90 °C, the gel liquefied.

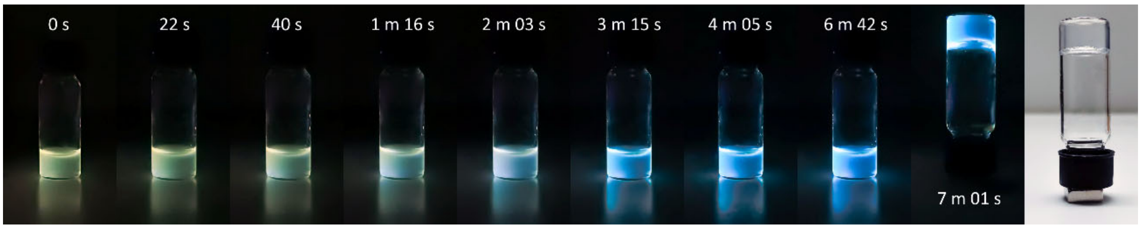

Figure S66. Cool-down process to rt of the hot sol from above after heating, under UV-light irradiation (365 nm). The annotated time is the time elapsed since the first picture, the estimated temperature of the at t = 0 s is ca. 80 °C - 85 °C.

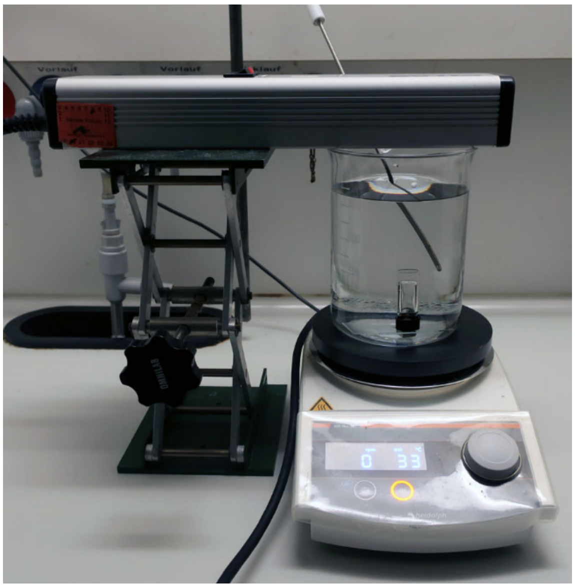

Figure S67. Setup with UV lamp, a beaker filled with water and a heating plate for taking pictures of the temperature dependent fluorescence of the gel. The vial is held down by a magnet inside the cap. Below the cap is a glass slide to ease positioning and under it is another magnet for fixation.

1, gluconolactone (GdL) gel from H_2O

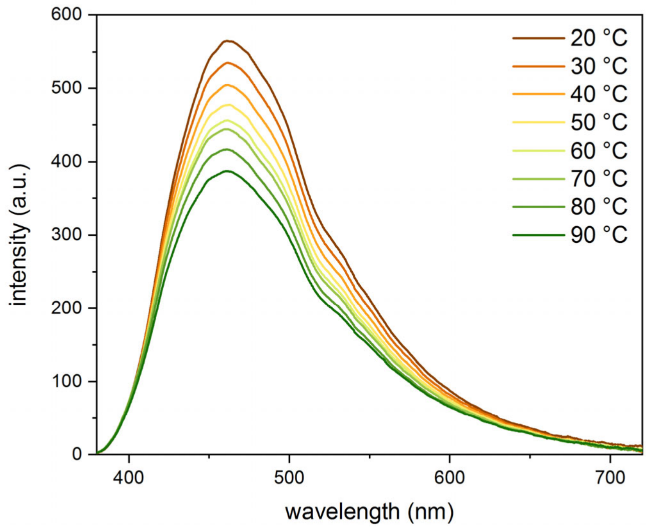

Figure S68. Temperature dependent fluorescence emission spectra while heating of the gel of **1** (1 wt%) obtained by adding GdL (5.0 equiv) to the sol of **1** in water. The gel had aged for 3 days prior to the measurement. Excitation at 365 nm.

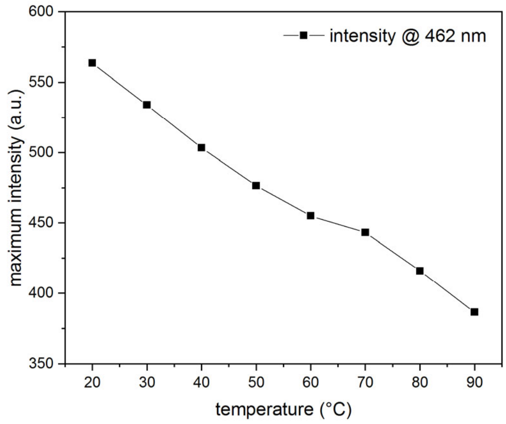

Figure S69. Temperature dependent intensity of the fluorescence emission maximum (462 nm) of the gel above.

7.4 Concentration dependent absorption- and fluorescence emission spectra of the aqueous sol of 1

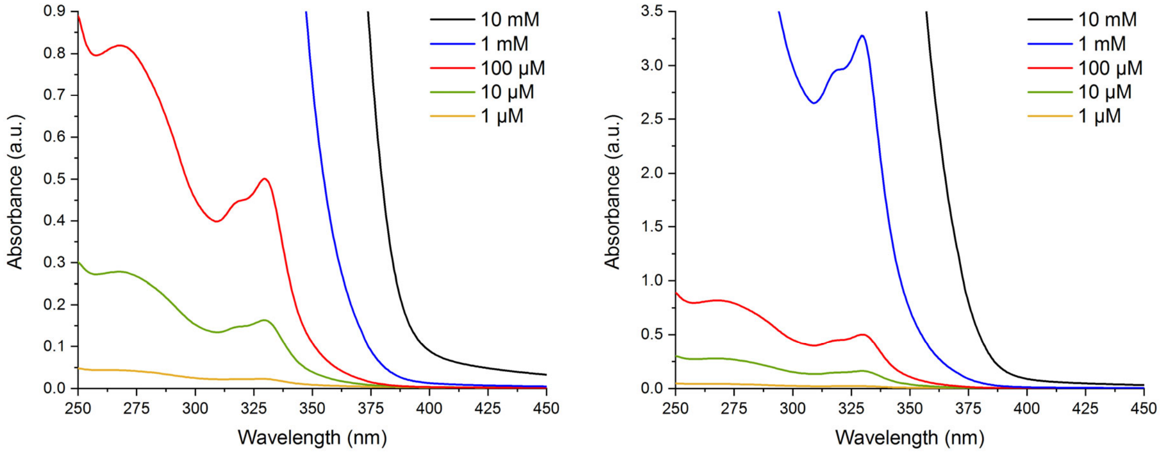

Figure S70. Concentration-dependent UV-absorption spectra of the sol of **1** in water (identical spectra with different scaling for better visualization). c = 10 mM, 1 mM were measured in 2x10 mm quartz cuvettes, c = 100 µM was measured in a 3x10 mm quartz cuvette, c = 10 µM and c = 1 µM were measured in 10x10 mm quartz cuvettes.

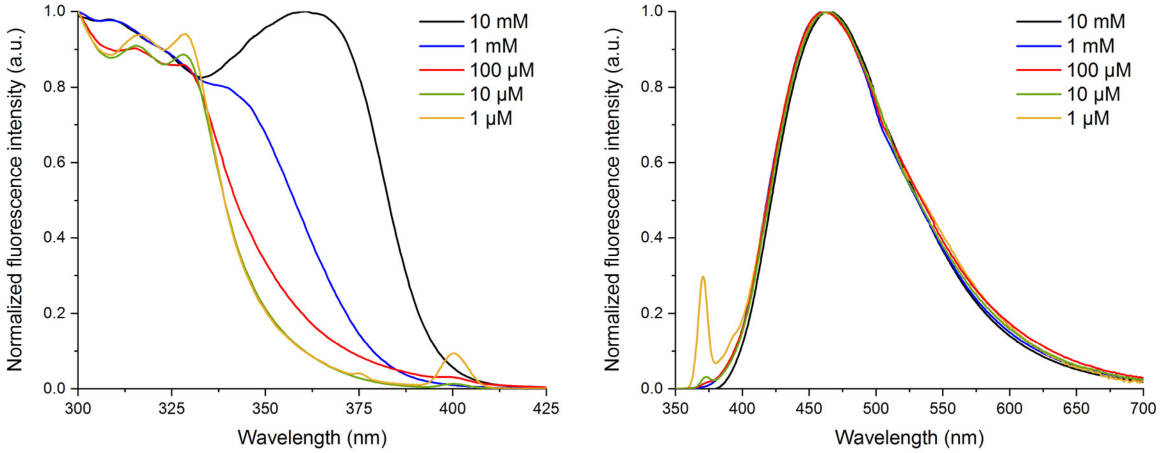

Figure S71. Normalized concentration-dependent excitation scans (emission at 466 nm, left) and normalized concentration-dependant fluorescence emission spectra of the sol of **1** in H_2O (right). Spectra on the right were excited at 365 nm (10 mM), 341 nm (1 mM) and 329 nm (100 µM, 10 µM, 1 µM). c = 10 mM, 1 mM were measured in 2x10 mm quartz cuvettes, c = 100 µM was measured in a 3x10 mm quartz cuvette, c = 10 µM and c = 1 µM were measured in 10x10 mm quartz cuvettes.

7.5 Overview and comparison of photophysical data for solids, gels and concentrated solutions

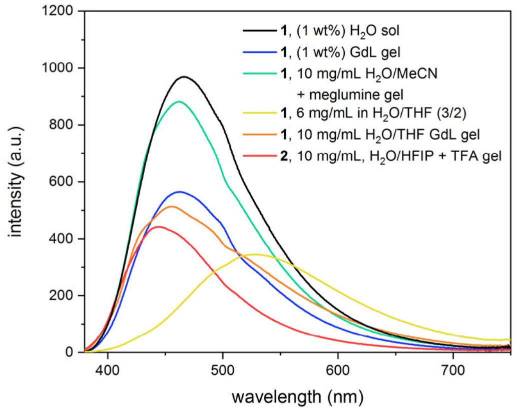

Figure S72. Comparison of fluorescence emission spectra (excitation at 365 nm) of non-solids. See above for details of the entries. See chapter 10.1 for details of gel compositions.

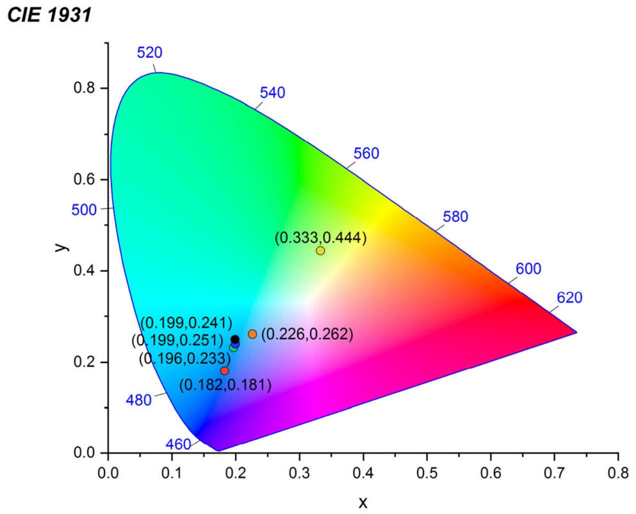

Figure S73. CIE 1931 chromaticity plot of the temperature dependent fluorescence emission spectra above with annotated emission color coordinates. The colors of the data points resemble the curves from graph above.

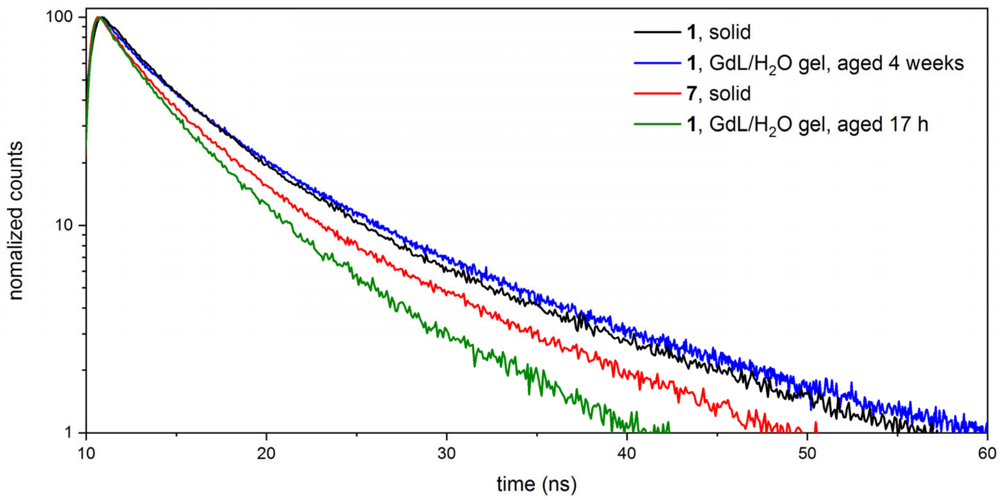

Figure S74. Comparison of fluorescence life-time decay profiles (excitation at 373 nm). Comparison of GdL gels of **1** to the solids **1** and **7**.

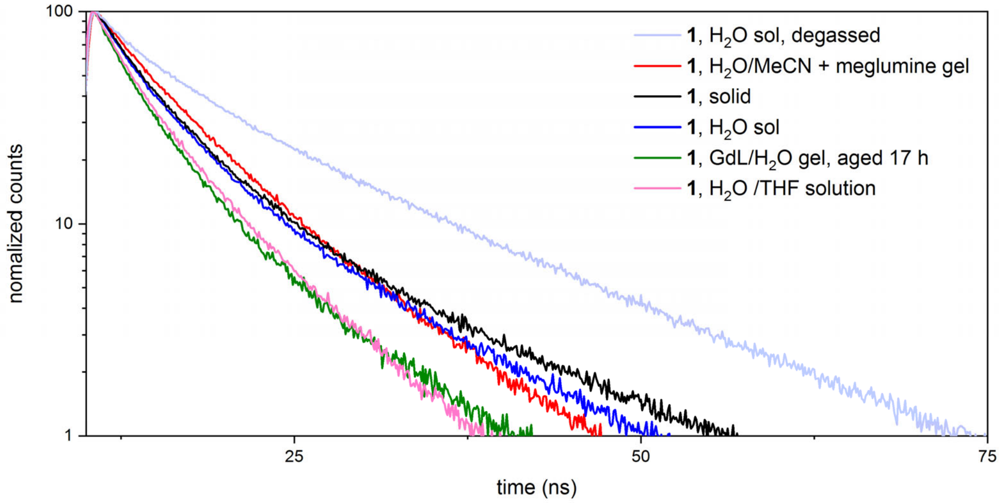

Figure S75. Comparison of fluorescence life-time decay profiles (excitation at 373 nm). Comparison of **1** as a solid, as a sol (aerated and deaerated), as a H_2O:THF solution and as two different gels.

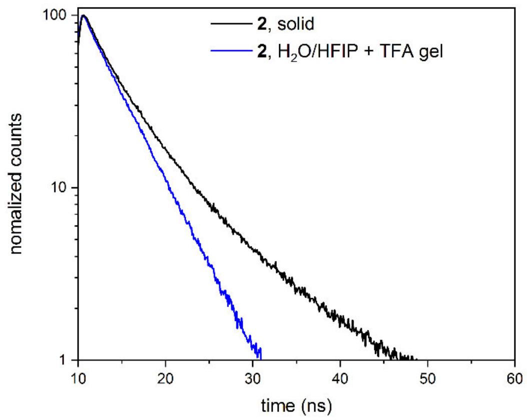

Figure S76. Comparison of fluorescence life-times of **2** as a solid and as a H_2O/HFIP (1:1) + TFA (1%) gel (excitation at 373 nm).

Table S7. Comparison of photophysical properties of gels, solids and concentrated solutions. See 10.1 for details of formulations.

Compound	Entry	λ_{em} [nm][a]	τ_{avg} [ns][b]	Φ_F [%][c]	k_r [10^8 s^{-1}][d]	k_{nr} [10^8 s^{-1}][d]
**1**	Solid	465	6.30 ± 0.16	3.9	0.062	1.53
**2**	Solid	466	5.54 ± 0.13	3.8	0.069	1.74
**7**	Solid	463	5.46 ± 0.17	3.0	0.055	1.78
**1**	Solution in THF/H_2O (2/3) (6.0 mg/mL)	527	4.67 ± 0.35	1.2	0.026	2.12
**1**	H_2O sol (1 wt%)	466	5.93 ± 0.13 (10.3 ± 0.1)[e]	3.5	0.059	1.63
**1**	GdL/H_2O gel 17 h old (1 wt%)	462	4.37 ± 0.14	2.5	0.057	2.23
**1**	GdL/H_2O gel 4 weeks old (1 wt%)	462	6.61 ± 0.08	2.3	0.035	1.48
**1**	H_2O/MeCN + meglumine gel (10 mg/mL)	462	6.45 ± 0.24	2.7	0.042	1.51
**2**	H_2O/HFIP + TFA gel 17 h old (10 mg/mL)	445	4.37 ± 0.11	1.9	0.043	2.24

[a] Excitation at 365 nm. [b] Excitation at 373 nm. [c] The absolute error of the measurement was $\Delta\Phi$ = ± 0.1%. [d] k_r and k_{nr} were calculated using the equations $k_r = \Phi_F/\tau_{avg}$ and $k_{nr} = (1–\Phi_F)/\tau_{avg}$. [e] Average fluorescence lifetime when deaerated.

8 SEM-images of hydro- and organogels

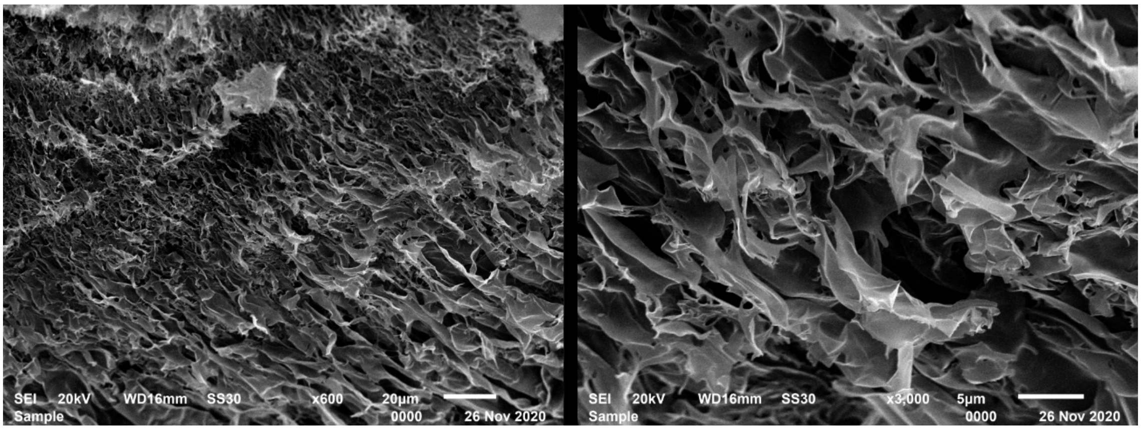

Figure S77. SEM images of the freeze-dried H_2O-sol of **1**. After freeze-drying the sol, a very fluffy solid was obtained. The thickness of the ribbons is ca. 60 nm - 200 nm

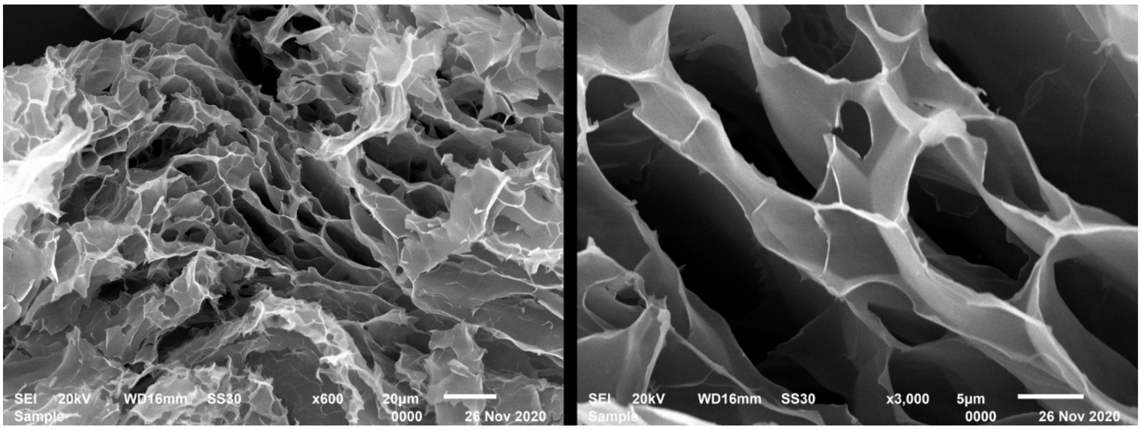

Figure S78. SEM images of the xerogel of the *n*Bu_4NBF_4/water gel of **1**. The thickness of the lamellar sheets in the right picture ranges approximately between 100 nm - 400 nm.

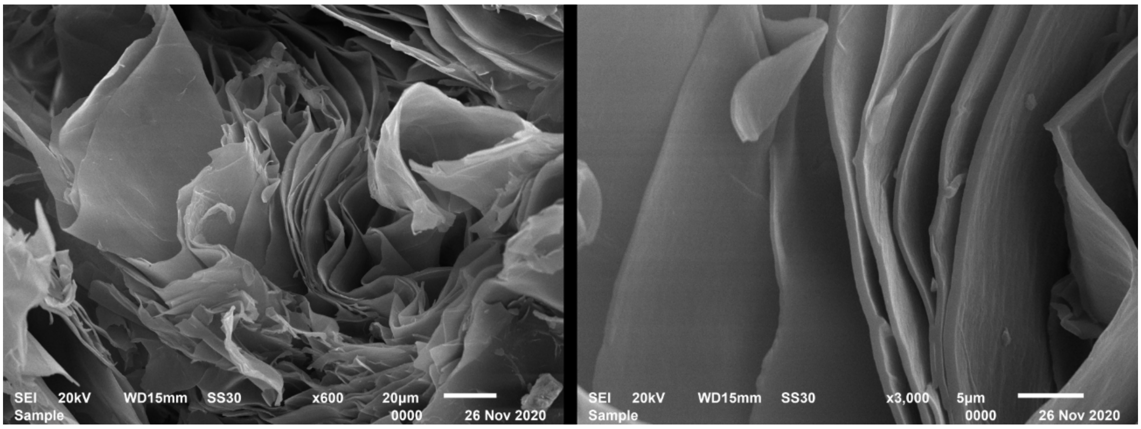

Figure S79. SEM images of the xerogel of the gluconolactone/water gel of **1**. The thickness of the lamellar sheets ranges approximately between 0.1 µm - 1 µm.

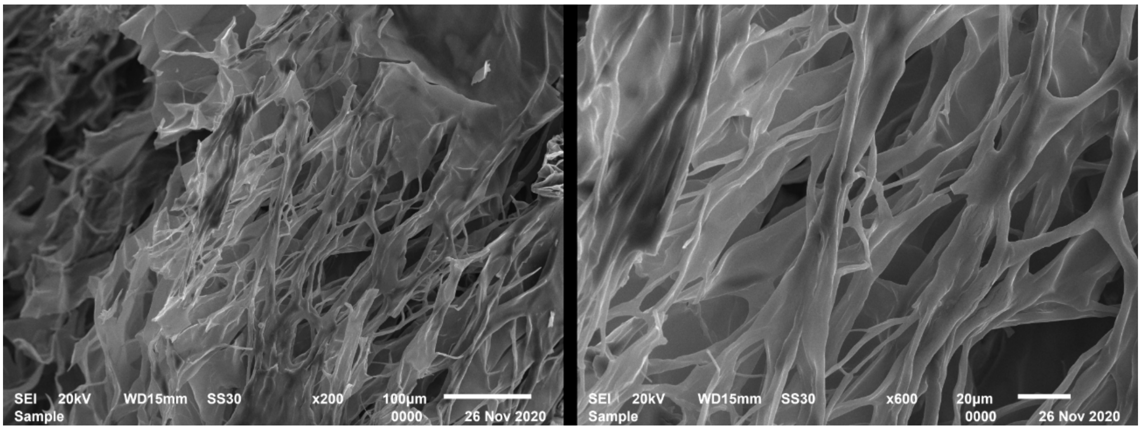

Figure S80. SEM images of the xerogel of the acetonitrile/water + meglumine gel of **1**. The thickness of the fibres ranges approximately between 1 μm - 10 μm.

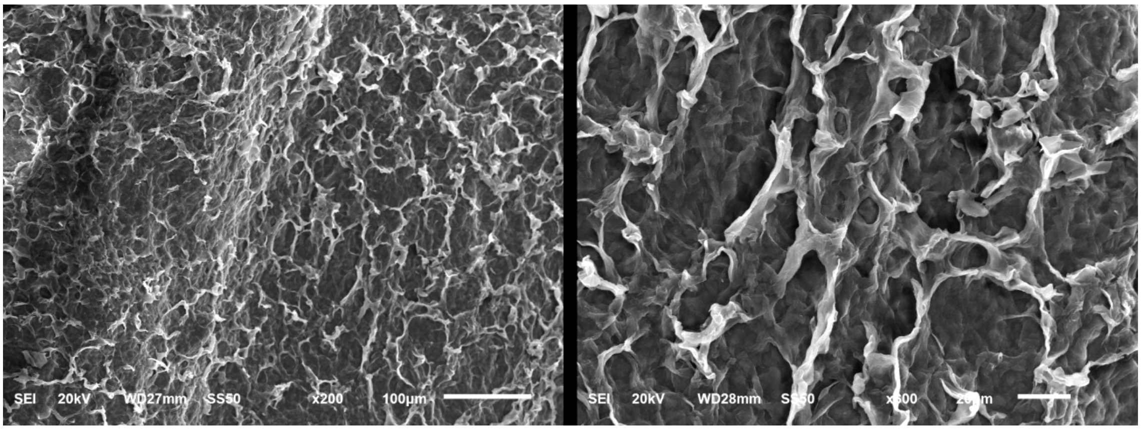

Figure S81. SEM images of the xerogel of the H_2O/HFIP + TFA gel of **2**.

9 Rheological experiments

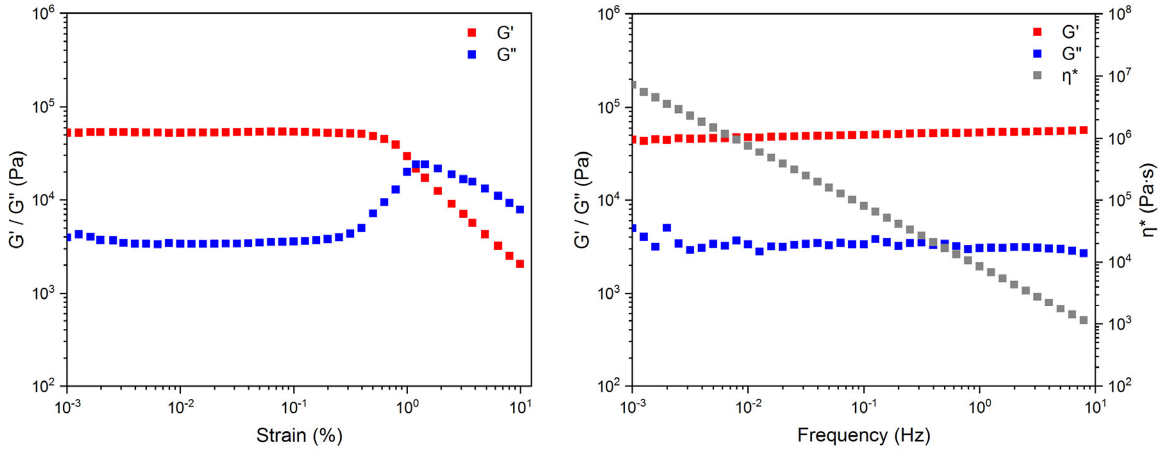

Figure S82. Amplitude sweep experiment (left) and frequency sweep experiment (right) of the GdL/H_2O gel of **1** (15 mg/mL, aged 7 days).

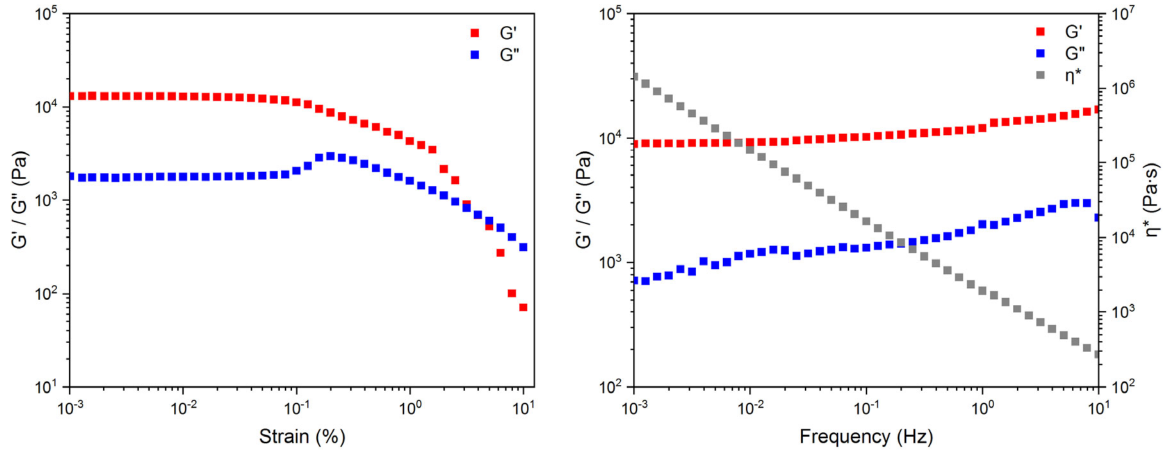

Figure S83. Amplitude sweep experiment (left) and frequency sweep experiment (right) of the H_2O/MeCN + meglumine gel of **1** (10 mg/mL, aged 7 days).

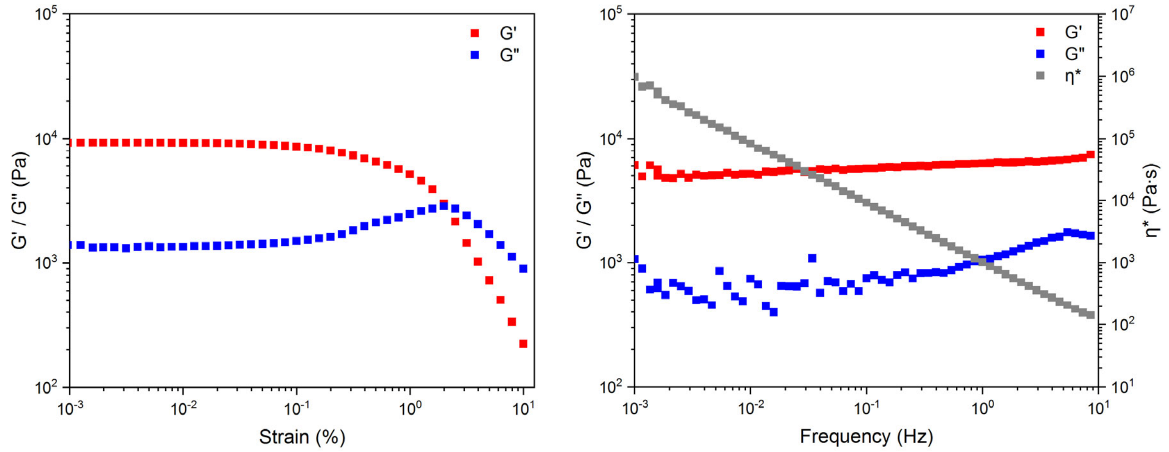

Figure S84. Amplitude sweep experiment (left) and frequency sweep experiment (right) of the H_2O/HFIP + TFA gel of **2** (15 mg/mL, aged 7 days).

10 Preparation of compounds

10.1 Overview and preparation of hydro- and organogels

Preparation of the H_2O sol and H_2O/THF solution of **1**:

1 (10 mg) was suspended in H_2O (1.0 mL) by vortexing until an opaque suspension was obtained. The suspension was heated at 80 °C for 10 min, which resulted in an almost clear sol.

The H_2O/THF solution of **1** was obtained by diluting the sol with the stated amount of THF and sonicating and vortexing it until no blue fluorescence could be seen anymore upon 365 nm UV-light irradiation.

Glucono-*δ*-lactone (GdL)/water gel of **1**:

GdL (>2.0 equiv, usually 3.0 - 5.0 equiv) was added to the sol of **1**. The mixture was quickly sonicated until GdL had completely dissolved and then vortexed for 10 s to homogenize it. In the same way, gels with different additives (e.g. $n$$Bu_4NBF_4$) were prepared.

H_2O/MeCN + *N*-Methyl-D-glucamine (meglumine) gel of **1**:

First, the sol of **1** was prepared as described above, using only 500 µL of H_2O. Then, meglumine (10 mg) and acetonitrile (500 µL) were added and the mixture was heated to 80 °C for 10 min. Upon cooling down, the gel formed, which was left standing for 17 h at rt.

H_2O/HFIP + TFA gel of **2**:

2 (10 mg) was dissolved in HFIP (500 µL) by adding TFA (15 µL), which led to a change in color of the suspension from a very slight yellow/green color to a bright yellow color, and sonicated until all solids had dissolved. Water (500 µL) was added and the mixture was very quickly vortexed for 10 s, then left standing for 17 h.

10.2 Preparation of L-serine methyl ester hydrochloride (H-Ser-OMe • HCl, S2)

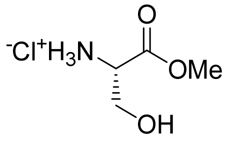

S2

A slightly modified literature procedure was used.[1] L-Serine (**S1**, 4.20 g, 40.0 mmol) was suspended in MeOH (50 mL) and cooled to 0 °C under a nitrogen atmosphere. Thionyl chloride (4.4 mL, 60.0 mmol) was added *via* a syringe pump at the rate of 0.5 mL/min, and the reaction mixture was stirred for 20 h while slowly warming to room temperature. The solvent and all volatiles were removed *in vacuo* to yield a colorless solid. Et_2O (100 mL) was added and the solid was filtered after stirring for 10 min at rt. The solid was washed with more Et_2O (2x 50 mL) and dried to yield **S2** as a colorless solid (5.86 g, 37.2 mmol, 93%).

^{1}H NMR (600 MHz, DMSO-d_6): δ = 8.66 (s, 3H), 6.10 – 4.99 (m, 1H), 4.05 (s, 1H), 3.81 (d, J = 3.5 Hz, 2H), 3.71 (s, 3H) ppm. ^{13}C NMR (151 MHz, DMSO-d_6): δ = 168.54, 59.50, 54.47, 52.80 ppm. IR (ATR): $\tilde{\nu}$ (cm^{-1}): 3340, 2917, 2662, 2634, 1926, 1745, 1592, 1509, 1472, 1444, 1431, 1382, 1344, 1297, 1251, 1159, 1129, 1094, 1038, 980, 967, 901, 845, 795. MS (APCI) m/z = 120.1 [M-Cl$^-$]$^+$. Mp. 159 °C.

10.3 Preparation of *N*-(*tert*-butoxycarbonyl)-L-serine methyl ester (Boc-Ser-OMe, S3)

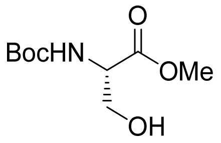

S3

A slightly modified literature procedure was used.[2] To a solution of H-Ser-OMe·HCl (**S2**, 5.60 g, 36.0 mmol) in DCM (100 mL) was added NEt_3 (10.0 mL, 72.0 mmol) and the resulting suspension was cooled to 0 °C after stirring for 10 min at rt. Boc_2O (8.73 g, 40.0 mmol) was added and the reaction mixture stirred for 20 h, slowly warming to rt. 1 M aqueous phosphoric acid (100 mL) was added and the phases were separated. The organic phase was washed with 1 M aqueous phosphoric acid and the combined aqueous phases were extracted with DCM (100 mL). The combined organic phases were washed with sat. $NaHCO_3$ (3x 100 mL) and brine (100 mL) and dried over

Na_2SO_4. After concentration, **S3** was obtained as a colorless oil (7.21 g, 32.9 mmol, 91%) and used directly without further purification.

^{1}H NMR (600 MHz, Chloroform-*d*): δ = 5.46 (s, 1H), 4.39 (s, 1H), 4.01 – 3.85 (m, 2H), 3.78 (s, 3H), 2.43 (t, *J* = 6.0 Hz, 1H), 1.45 (s, 9H) ppm. ^{13}C NMR (151 MHz, Chloroform-*d*): δ = 171.51, 155.90, 80.42, 63.55, 55.81, 52.74, 28.39 ppm. IR (ATR): $\tilde{\nu}$ (cm^{-1}): 3391, 2977, 1689, 1506, 1456, 1437, 1392, 1366, 1282, 1209, 1159, 1058, 1030, 851, 779, 759. MS (APCI) *m/z* = 252.1 [M+MeOH+H]$^+$.

10.4 Preparation of *N*-(*tert*-butoxycarbonyl)-3-iodo-L-alanine methyl ester (S4)

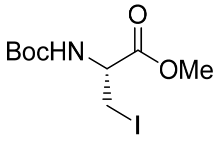

S4

A slightly modified literature procedure was used.[3] A dried Schlenk flask was charged with triphenylphosphine (18.5 g, 70.6 mmol), imidazole (4.81 g, 70.6 mmol) and dry DCM (140 mL). The flask was cooled to 0 °C and iodine (17.9 g, 70.6 mmol) was added in the dark in three portions over five minutes. After 30 min, the ice bath was removed and further 20 min later, the solution was cooled down to 0 °C again. and Boc-Ser-OMe (**S3**, 11.9 g, 54.3 mmol) in DCM (50 mL) was added *via* a syringe pump at a rate of 2 mL/min. 1 h after complete addition, the reaction mixture was stirred at rt for another 1.5 h. The reaction was quenched by the addition of sat. Na_2SO_3 (50 mL) and water (50 mL). The aqueous phase was extracted with DCM (5x 50 mL), and the combined organic phases were dried over Na_2SO_4. After removal of the solvent, Et_2O (150 mL) was added to the residue to precipitate triphenylphosphine oxide. The suspension was cooled to 5 °C for 30 min, then quickly filtered and washed with cold Et_2O (2x 50 mL). The filtrate was concentrated and the residue purified by column chromatography (cyclohexane/ethyl acetate 8+1), which was performed in the dark to avoid rapid light-triggered HI-elimination on silica. The fractions containing the product were combined, concentrated and crystallized from pentane at -20 °C to give **S4** as a colorless, crystalline solid (11.6 g, 35.2 mmol, 65%).

^{1}H NMR (600 MHz, Chloroform-*d*): δ = 5.50 – 4.92 (m, 1H), 4.51 (dt, *J* = 8.0, 4.0 Hz, 1H), 3.79 (s, 3H), 3.56 (qd, *J* = 10.1, 3.7 Hz, 2H), 1.45 (s, 9H) ppm. ^{13}C NMR (151 MHz, Chloroform-*d*): δ = 170.17, 154.94, 80.60, 53.79, 53.13, 28.40, 7.98 ppm. IR (ATR): $\tilde{\nu}$

(cm^{-1}): 3348, 2981, 1732, 1689, 1522, 1438, 1418, 1393, 1367, 1313, 1273, 1248, 1221, 1206, 1156, 1059, 1032, 1009, 980, 906, 863, 829, 791, 775, 760, 741, 647. MS (APCI) *m/z* = 330.0 $[M+H]^+$. Mp. 47 - 49 °C.

10.5 Preparation of 3-iodo-9*H*-carbazole (S6)

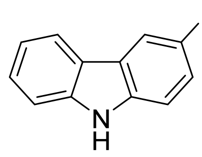

S6

A slightly modified literature procedure was used.[4] Carbazole (**S5**, 10.0 g, 60.0 mmol), $NaIO_4$ (1.93 g, 9.00 mmol), iodine (6.86 g, 27.0 mmol) and conc. H_2SO_4 (6.5 mL, 120 mmol) were suspended in *i*PrOH (240 mL) and heated to 65 °C for 6 h. After cooling to rt, the reaction mixture was diluted with water (100 mL), carefully neutralized with 5 N NaOH and then solid Na_2SO_3 (2.5 g, 20.0 mmol) was added. The reaction mixture was concentrated to remove most of the *i*PrOH and the volume adjusted to about 200 mL using water. DCM (250 mL) was added and the phases were separated. The aqueous phase was extracted with DCM (2x 50 mL) and the combined organic phases were concentrated. The solid residue was recrystallized from a mixture of 150 mL water and 500 mL EtOH to yield **S6** as pearlescent crystals (8.52 g, 29.0 mmol, 48%).

^{1}H NMR (601 MHz, DMSO-d_6): δ = 11.40 (s, 1H), 8.51 (s, 1H), 8.15 (d, *J* = 7.8 Hz, 1H), 7.64 (d, *J* = 8.4 Hz, 1H), 7.49 (d, *J* = 8.1 Hz, 1H), 7.41 (t, *J* = 7.6 Hz, 1H), 7.35 (d, *J* = 8.5 Hz, 1H), 7.17 (t, *J* = 7.5 Hz, 1H) ppm. ^{13}C NMR (151 MHz, DMSO-d_6): δ = 139.74, 138.78, 133.34, 128.67, 126.25, 125.17, 121.17, 120.62, 118.95, 113.40, 111.09, 81.32 ppm. IR (ATR): $\tilde{\nu}$ (cm^{-1}): 3401, 3048, 1595, 1465, 1442, 1331, 1270, 1239, 1133, 1044, 1016, 1001, 928, 880, 801, 745, 724. MS (APCI) *m/z* = 294.0 $[M+H]^+$. Mp. 192 °C.

10.6 Preparation of 2-chloro-4,6-diphenyl-1,3,5-triazine (S8)

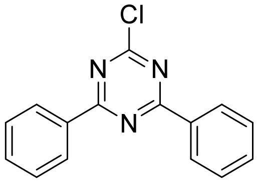

S8

A slightly modified literature procedure was used.[5] In an oven-dried 500 mL 2-neck flask with a dropping funnel, magnesium turnings (3.74 g, 154 mmol) were dried *in vacuo* using a heat gun. Then, under a N_2 atmosphere, dry THF (50 mL) and 1,2-dibromoethane (0.30 mL, 3.5 mmol) were added and the mixture was stirred for 15 min at rt. A solution of bromobenzene (17.6 mL, 168 mmol) in dry THF (80 mL) was added to the dropping funnel and about ten percent of the solution was added at once to the activated magnesium/THF mixture. After a noticeable exothermic reaction, a water bath was placed under the flask and the rest of the bromobenzene-solution was added dropwise over 15 min. The water bath was replaced by an oil bath and the reaction mixture was stirred for 1 h at 40 °C. It was cooled to rt and a solution of cyanuric chloride (**S7**, 12.9 g, 70.0 mmol) in dry THF (60 mL) was added over 15 min. The reaction mixture was stirred for 24 h at 35 °C and afterwards 1 N HCl (200 mL) was added to it. The phases were separated and the aqueous phase was extracted with cyclohexane (2x 100 mL). The combined organic phases were dried over Na_2SO_4 and concentrated. The solid residue was heated to reflux for 10 min in EtOH (200 mL), allowed to cool to rt and then cooled to 5 °C for 1 h. It was filtered quickly and washed with MeOH (100 mL) to yield **S8** as a brownish solid (10.1 g, 37.7 mmol, 54%).

^{1}H NMR (600 MHz, Chloroform-*d*): δ = 8.60 (d, J = 7.7 Hz, 2H), 7.62 (t, J = 7.4 Hz, 1H), 7.54 (t, J = 7.6 Hz, 2H) ppm. ^{13}C NMR (151 MHz, Chloroform-*d*): δ = 173.42, 172.24, 134.42, 133.65, 129.48, 128.90 ppm. IR (ATR): $\tilde{\nu}$ (cm^{-1}): 1537, 1492, 1443, 1369, 1327, 1245, 1078, 1001, 846, 749, 685, 667. MS (APCI) *m/z* = 268.1 [M+H]$^{+}$. Mp. 135 - 137 °C.

10.7 Preparation of 9-(4,6-diphenyl-1,3,5-triazin-2-yl)-3-iodo-9*H*-carbazole (3)

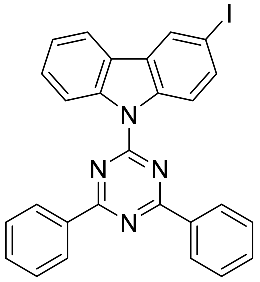

3

A slightly modified literature procedure was used.[6] 2-chloro-4,6-diphenyl-1,3,5-triazine (**S8**, 7.84 g, 29.3 mmol) and 3-iodo-9*H*-carbazole (**S6**, 8.60 g, 29.3 mmol) were placed in a dried Schlenk flask under a nitrogen atmosphere, dissolved in dry THF (90 mL) and cooled to 0 °C. Sodium hydride (60% dispersion in mineral oil, 1.29 g, 32.2 mmol) was added carefully over 10 min and the mixture was stirred for 22 h, warming slowly to rt. Acetic acid (0.5 mL) was added, and the precipitated solid was filtered and washed with water (200 mL). The filtered solid was heated to reflux in a 1:1 mixture of acetone and water (100 mL) and filtered while still hot. The same procedure was repeated with acetone (100 mL) and finally EtOAc (100 mL). After drying, **3** was obtained as an off-white solid (12.7 g, 24.1 mmol, 82%).

^{1}H NMR (600 MHz, Chloroform-*d*): δ = 8.96 (d, J = 8.4 Hz, 1H), 8.70 (d, J = 8.8 Hz, 1H), 8.35 (d, J = 7.8 Hz, 4H), 8.32 (d, J = 1.8 Hz, 1H), 7.96 (dd, J = 7.6, 1.3 Hz, 1H), 7.94 – 7.86 (m, 3H), 7.75 (t, J = 7.7 Hz, 4H), 7.63 (t, J = 7.8 Hz, 1H), 7.56 (t, J = 7.5 Hz, 1H) ppm. ^{13}C NMR (151 MHz, Chloroform-*d*): δ = 166.67, 161.66, 138.48, 137.87, 137.16, 136.94, 130.97, 130.31, 129.83, 129.65, 129.24, 128.55, 127.50, 127.42, 121.44, 120.76, 119.92, 91.75 ppm. HR-MS (ESI): calculated for $C_{27}H_{18}IN_4^+$[M+H]$^+$: m/z = 525.05707, found: 525. 05652. IR (ATR): $\tilde{\nu}$ (cm^{-1}): 3057, 1588, 1526, 1483, 1444, 1413, 1302, 1208, 1176, 817, 801, 764, 744, 717, 702, 684, 665, 655, 644. Mp. 275 - 280 °C (decomp.).

<u>Note:</u> When this reaction was performed using DMF as the solvent, the yield was 96%. The product of this reaction, however, led to unreliable yields for the following cross-coupling reaction.

10.8 Preparation of *N*-(*tert*-butoxycarbonyl)-3-(9-(4,6-diphenyl-1,3,5-triazin-2-yl)-9*H*-carbazol-3-yl)-L-alanine methyl ester (Boc-Ala(3-(DPhCz(3-)T))-OMe) (4)

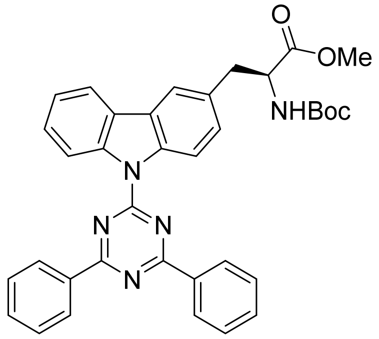

4

The cross-coupling was performed according to a slightly modified literature procedure.[7] Zinc was activated according to a slightly modified literature procedure:[8] Zinc dust (6.86 g, 105 mmol) was suspended in 1 N HCl (15 mL) for 3 min. It was washed with MeOH (3x 20 mL) and Et_2O (3x 20 mL) and then placed in a Schlenk flask. The activated zinc was rigorously dried *in vacuo* using a heat gun. Dry DMF (53 mL) and iodine (800 mg, 3.15 mmol) were added under a nitrogen atmosphere and stirred for 5 min. After cooling to 0 °C, methyl *N*-(*tert*-butoxycarbonyl)-3-iodo-L-alanine methyl ester (**S4**, 6.91 g, 21.0 mmol) was added and the cooling was removed. The mixture was stirred for 3 h at rt and afterwards $Pd_2(dba)_3$ (612 mg, 668 μmol), SPhos (550 mg, 1.34 mmol), 9-(4,6-diphenyl-1,3,5-triazin-2-yl)-3-iodo-9*H*-carbazole (**3**, 10.0 g, 19.1 mmol) and dry THF (64 mL) were added in this order. The mixture was stirred for 21 h at 30 °C under a positive pressure of nitrogen and then poured into a mixture of toluene (500 mL), water (100 mL) and sat. NH_4Cl (100 mL). The mixture was filtered and the organic phase of the filtrate was washed with brine (3x 100 mL). The filtered solid was extracted with DCM (3x 100 mL), and the DCM-filtrate was subsequently washed with brine (2x 50 mL). The combined organic phases were dried over Na_2SO_4. Silica gel was added, the suspension was concentrated, dried and purified by column chromatography (DCM). The fractions containing the product were combined, concentrated and acetone (200 mL) was added. The resulting suspension was stored for 2 hr at -20 °C, then filtered, washed with acetone (50 mL) and dried to yield **4** as a colorless solid (5.85 g, 9.77 mmol, 51%).

^{1}H NMR (600 MHz, Chloroform-*d*): δ = 9.14 (d, *J* = 8.4 Hz, 1H), 9.06 (d, *J* = 8.5 Hz, 1H), 8.74 (dt, *J* = 6.8, 1.5 Hz, 4H), 8.03 (d, *J* = 7.6 Hz, 1H), 7.82 (d, *J* = 1.8 Hz, 1H), 7.62 (tdd, *J* = 15.9, 7.7, 6.3 Hz, 7H), 7.43 (t, *J* = 7.4 Hz, 1H), 7.37 (dd, *J* = 8.6, 1.8 Hz,

1H), 5.08 (d, *J* = 8.2 Hz, 1H), 4.71 (q, *J* = 6.8 Hz, 1H), 3.75 (s, 3H), 3.41 – 3.06 (m, 2H), 1.44 (s, 9H) ppm. ^{13}C NMR (151 MHz, Chloroform-*d*): δ = 172.68, 172.55, 165.23, 155.34, 139.51, 138.37, 136.36, 132.86, 131.18, 129.25, 128.95, 128.26, 127.28, 127.07, 126.52, 123.46, 120.32, 119.72, 117.99, 117.97, 80.16, 54.97, 52.45, 38.47, 28.48 ppm. HR-MS (ESI): calculated for $C_{36}H_{34}N_5O_4^+[M+H]^+$: m/z = 600.26053, found: 600.26004. IR (ATR): $\tilde{\nu}$ (cm^{-1}): 3370, 2978, 1739, 1696, 1686, 1589, 1527, 1500, 1485, 1456, 1443, 1373, 1328, 1249, 1173, 1056, 1018, 833, 765, 704, 685. Mp. 206 °C.

Note: If aryl iodide **3** was synthesized from DMF and then used for this reaction, yields varied strongly between 0% and 50%. When **3,** synthesized by employing THF as reaction solvent, was used, yields were very consistent, around 45-51%, across milligram and gram scales as well.

10.9 Preparation of Boc-Lys(Z)-Ala(3-(DPhCz(3-)T))-OMe (S10) (*N*²-(*tert*-butoxycarbonyl)-*N*⁶-(benzyloxycarbonyl)-L-lysyl-3-(9-(4,6-diphenyl-1,3,5-triazin-2-yl)-9*H*-carbazol-3-yl)-L-alanine methyl ester)

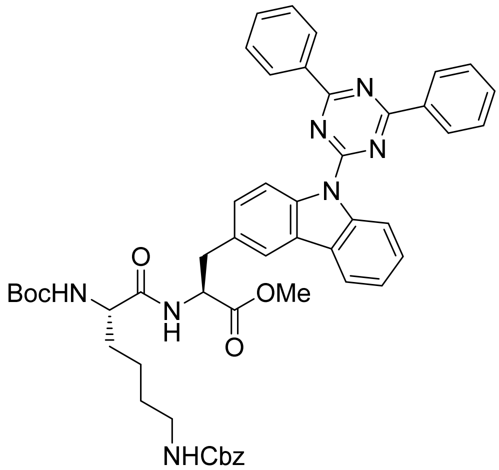

S10

Boc-Ala(3-(DPhCz(3-)T)-OMe (**4**, 1.20 g, 2.00 mmol) was suspended in dry DCM (20 mL) and TFA (1.54 mL, 20.0 mmol) was added. The suspension quickly turned into a bright yellow solution that was stirred for 18 h at rt. Next, the solvent was removed and remaining TFA was co-evaporated with DCM, MeOH and toluene until a colorless solid was obtained. The solid was dried and then placed in an oven-dried Schlenk flask under nitrogen atmosphere. It was dissolved in a mixture of dry DCM (25 mL) and dry DMF (8 mL). NEt_3 (616 µL, 4.40 mmol) and Boc-Lys(Z)-OH (761 mg, 2.00 mmol) were added and the solution was cooled to 0 °C. HBTU (759 mg, 2.00 mmol) was added

and the reaction mixture was stirred for 2.5 d, slowly warming to rt. Afterwards, MeOH (30 mL) was added and the precipitated solids were filtered and washed with acetone (100 mL). After drying, **S10** was obtained as a colorless solid (1.55 g, 1.80 mmol, 90%).

^{1}H NMR (600 MHz, DMSO-d_6) δ = 9.09 (d, J = 8.4 Hz, 1H), 8.99 (d, J = 8.4 Hz, 1H), 8.80 – 8.64 (m, 4H), 8.25 (t, J = 7.1 Hz, 2H), 8.12 (s, 1H), 7.81 – 7.70 (m, 6H), 7.69 – 7.63 (m, 1H), 7.52 (d, J = 8.3 Hz, 1H), 7.47 (t, J = 7.3 Hz, 1H), 7.39 – 7.23 (m, 5H), 7.13 (t, J = 5.6 Hz, 1H), 6.80 (d, J = 8.2 Hz, 1H), 4.94 (s, 2H), 4.67 (dt, J = 14.1, 6.9 Hz, 1H), 3.97 – 3.69 (m, 1H), 3.63 (s, 3H), 3.26 (d, J = 5.6 Hz, 1H), 3.17 (dd, J = 13.9, 8.8 Hz, 1H), 2.86 (q, J = 6.7 Hz, 2H), 1.52 – 1.38 (m, 2H), 1.28 (s, 11H), 1.17 – 1.08 (m, 2H) ppm. ^{13}C NMR (151 MHz, DMSO-d_6) δ = 172.40, 171.92, 171.80, 164.61, 155.99, 155.20, 138.55, 137.24*, 137.20*, 135.51, 133.22, 132.50, 129.20, 128.78, 128.59, 128.32, 127.71, 127.35, 126.07, 125.98, 123.58, 120.59, 120.21, 117.43, 117.13, 77.95, 65.06, 54.28, 53.58, 51.93, 40.01, 36.54, 31.61, 29.07, 28.08, 22.71 ppm.

HR-MS (ESI): calculated for $C_{50}H_{52}N_7O_7^+$[M+H]$^+$: m/z = 862.39227, found: 862.39159; calculated for $C_{50}H_{51}N_7NaO_7^+$[M+Na]$^+$: m/z = 884.37422, found: 884.37333. calculated for $C_{50}H_{51}KN_7O_7^+$[M+K]$^+$: m/z = 900.34816, found: 900.34736. IR (ATR): $\tilde{\nu}$ (cm^{-1}): 3319, 2943, 1733, 1684, 1648, 1525, 1483, 1456, 1374, 1347, 1267, 1248, 1171, 1025, 766, 742, 683, 644. Mp. 235 - 238 °C.

10.10 Preparation of cyclo-(Ala(3-(DPhCz(3-)T))-Lys(Z)) (5) (Benzyl (4-((2*S*,5*S*)-5-((9-(4,6-diphenyl-1,3,5-triazin-2-yl)-9*H*-carbazol-3-yl)methyl)-3,6-dioxopiperazin-2-yl)butyl)carbamate)

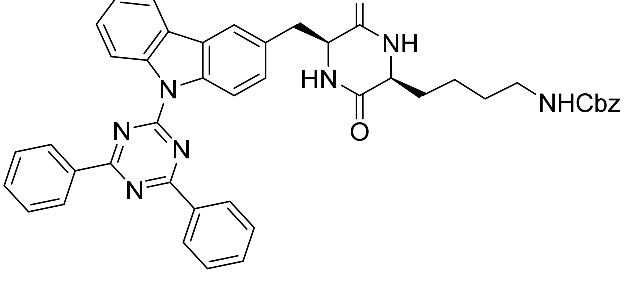

5

A slightly modified literature procedure was used.[9] Boc-Lys(Z)-Ala(3-(DPhCz(3-)T))-OMe (**S10**, 216 mg, 250 µmol) was suspended in dry DCM (5 mL) and TFA (191 µL, 2.50 mmol) was added. The suspension quickly turned into a bright yellow solution that

was stirred for 18 h at rt. Next, the solvent was removed and remaining TFA was co-evaporated with DCM, MeOH and toluene until a colorless solid was obtained. The solid was dried and rigorously ground to a fine powder. Next, the powder was suspended MeCN (2.5 mL) and 2-methylpropan-1-ol (2.5 mL) and NEt_3 was added (70 µL, 0.50 mmol). After stirring for 10 min at rt, AcOH was added (57 µL, 1.00 mmol) and the mixture was heated to 100 °C for 3 h. After cooling to rt, the suspension was filtered and washed with acetone (50 mL). After drying, **5** was obtained as a colorless solid (156 mg, 214 µmol, 90%).

^{1}H NMR (600 MHz, Chloroform-*d*+TFA): δ = 9.01 (d, J = 8.5 Hz, 2H), 8.37 (d, J = 7.7 Hz, 4H), 8.29 (s, 1H), 8.24 (s, 1H), 8.00 (d, J = 7.6 Hz, 1H), 7.96 – 7.87 (m, 3H), 7.74 (t, J = 7.7 Hz, 4H), 7.64 (t, J = 7.9 Hz, 1H), 7.56 (t, J = 7.5 Hz, 1H), 7.43 (d, J = 8.5 Hz, 1H), 7.40 – 7.33 (m, 3H), 7.29 – 7.23 (m, 2H, overlaid by residual $CHCl_3$ signal), 5.06 (s, 2H), 4.68 (s, 1H), 4.05 (t, J = 6.2 Hz, 1H), 3.58 – 3.36 (m, 2H), 2.84 (t, J = 6.6 Hz, 2H), 1.71 – 1.53 (m, 1H), 1.26 – 1.16 (m, 1H), 1.19 – 0.98 (m, 3H), 0.96 (dq, J = 9.0, 4.6 Hz, 1H) ppm. ^{13}C NMR (151 MHz, Chloroform-*d*+TFA): δ = 170.69, 169.71, 166.94, 158.98, 138.76, 138.10, 136.77, 135.07, 132.82, 130.20, 130.03, 129.80, 129.72, 129.16, 128.89, 128.84, 128.70, 128.27, 127.97, 127.35, 121.63, 120.51, 120.41, 119.90, 68.64, 56.34, 54.59, 40.73, 39.50, 33.51, 28.55, 21.58 ppm. HR-MS (ESI): calculated for $C_{44}H_{40}N_7O_4^+$[M+H]$^+$: m/z = 730.31363, found: 730.31275; calculated for $C_{44}H_{39}NaN_7O_4^+$[M+Na]$^+$: m/z = 752.29557, found: 752.29474; calculated for $C_{44}H_{39}KN_7O_4^+$[M+K]$^+$: m/z = 768.26951, found: 768.26866. IR (ATR): $\tilde{\nu}$ (cm^{-1}): 3313, 3055, 2955, 2896, 1675, 1587, 1525, 1484, 1452, 1371, 1330, 1250, 767, 705, 655, 644. Mp. 283 °C (decomp.).

Note: Without grinding, no full conversion to the DKP could be achieved due to the insolubility of the deprotected dipeptide.

10.11 Preparation of *cyclo*-(Ala(3-(DPhCz(3-)T))-Lys) hydrobromide (2) ((3*S*,6*S*)-3-(4-aminobutyl)-6-((9-(4,6-diphenyl-1,3,5-triazin-2-yl)-9*H*-carbazol-3-yl)methyl)piperazine-2,5-dione hydrobromide)

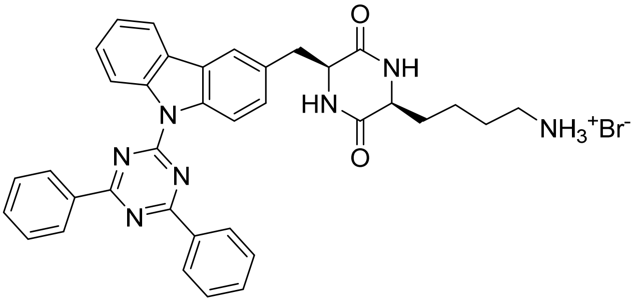

2

A slightly modified literature procedure was used.[10] *cyclo*-(Ala(3-(DPhCz(3-)T))-Lys(Z)) (**5**, 677 mg, 0.93 mmol) was dissolved in TFA (3 mL) using an ultrasonic bath, and AcOH (3 mL) and HBr (5.7 M in AcOH, 1.75 mL, 10.0 mmol) were added. The reaction mixture was stirred for 75 min at rt and then added dropwise to a solution of pyridine (3 mL) in EtOH (20 mL). After stirring for 10 min, the precipitated solid was filtered and washed with acetone (50 mL), EtOAc (50 mL) and DCM (50 mL). The washed solid was heated to reflux in EtOAc (50 mL), filtered while still hot and washed with DCM (20 mL). After drying, **2** was obtained as an off-white solid (540 mg, 0.80 mmol, 86%).

^{1}H NMR (601 MHz, Chloroform-*d*+TFA): δ = 9.04 (dd, *J* = 13.9, 8.4 Hz, 2H), 8.40 (d, *J* = 7.7 Hz, 4H), 8.37 (s, 1H), 8.20 (s, 1H), 8.02 (d, *J* = 7.6 Hz, 1H), 7.98 – 7.89 (m, 3H), 7.78 (t, *J* = 7.6 Hz, 4H), 7.69 (t, *J* = 7.8 Hz, 1H), 7.61 (t, *J* = 7.4 Hz, 1H), 7.46 (d, *J* = 8.4 Hz, 1H), 6.66 (s, 3H), 4.75 (t, *J* = 4.9 Hz, 1H), 4.09 (t, *J* = 6.1 Hz, 1H), 3.61 (dd, *J* = 14.5, 4.9 Hz, 1H), 3.39 (dd, *J* = 14.5, 4.5 Hz, 1H), 2.75 (q, *J* = 6.4 Hz, 2H), 1.45 (td, *J* = 9.8, 5.2 Hz, 1H), 1.36 (q, *J* = 7.3 Hz, 1H), 1.29 (q, *J* = 7.1 Hz, 1H), 1.17 – 0.98 (m, 2H), 0.96 – 0.82 (m, 1H) ppm. ^{13}C NMR (151 MHz, Chloroform-*d*+TFA): δ = 170.20, 169.25, 166.30, 161.75, 138.52, 137.82, 136.77, 132.57, 130.07, 129.94, 129.71, 129.52, 129.13, 128.25, 127.69, 127.35, 121.85, 120.33, 120.16, 119.79, 56.02, 53.98, 40.07, 38.82, 32.64, 25.80, 20.66 ppm. HR-MS (ESI): calculated for $C_{36}H_{34}N_7O_2^+[M-Br^-]^+$: m/z = 596.27685, found: 596.27597. IR (ATR): $\tilde{\nu}$ (cm^{-1}): 2895, 1673, 1587, 1524, 1443, 1370, 1213, 833, 765, 740, 704, 685, 655, 644. Mp. 273 °C (decomp.).

10.12 Preparation of Boc-Asp(OBn)-Ala(3-(DPhCz(3-)T))-OMe (S11) (***N*-(*tert*-butoxycarbonyl)-*O*[4]-benzyl-L-aspartyl-3-(9-(4,6-diphenyl-1,3,5-triazin-2-yl)-9*H*-carbazol-3-yl)-L-alanine methyl ester**)

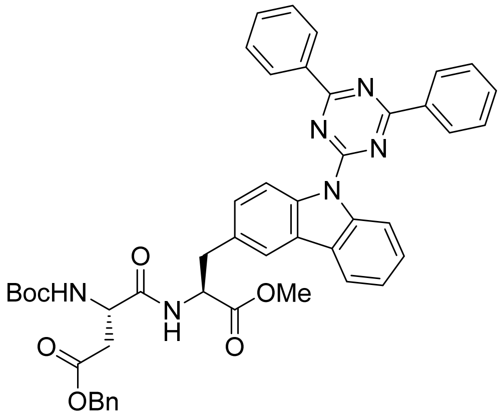

S11

Boc-Ala(3-(DPhCz(3-)T)-OMe (**4**, 1.20 g, 2.00 mmol) was suspended in dry DCM (20 mL) and TFA (1.54 mL, 20.0 mmol) was added. The suspension quickly turned into a bright yellow solution that was stirred for 18 h at rt. The solvent was removed and remaining TFA was co-evaporated with DCM, MeOH and toluene until a colorless solid was obtained. The solid was dried and then placed in an oven-dried Schlenk flask under nitrogen atmosphere. It was dissolved in a mixture of dry DCM (25 mL) and dry DMF (8 mL). NEt_3 (616 µL, 4.40 mmol) and Boc-Asp(OBn)-OH (647 mg, 2.00 mmol) were added and the solution was cooled to 0 °C. HBTU (759 mg, 2.00 mmol) was added and the reaction mixture was stirred for 2.5 d, slowly warming to rt. Afterwards, MeOH (30 mL) was added and the precipitated solids were filtered and washed with acetone (100 mL). After drying, **S11** was obtained as a colorless solid (1.40 g, 1.74 mmol, 87%).

^{1}H NMR (601 MHz, Chloroform-*d*): δ = 9.12 (d, *J* = 8.4 Hz, 1H), 9.07 (d, *J* = 8.5 Hz, 1H), 8.73 (dt, *J* = 6.9, 1.5 Hz, 4H), 8.14 (d, *J* = 7.6 Hz, 1H), 7.91 (s, 1H), 7.68 – 7.57 (m, 7H), 7.42 (td, *J* = 7.5, 0.9 Hz, 1H), 7.35 (dd, *J* = 8.5, 1.8 Hz, 1H), 7.33 – 7.20 (m, 5H, overlaid by residual $CHCl_3$), 7.08 (d, *J* = 7.6 Hz, 1H), 5.69 (d, *J* = 8.9 Hz, 1H), 5.12 (d, *J* = 12.3 Hz, 1H), 5.05 (d, *J* = 12.3 Hz, 1H), 4.94 (dt, *J* = 7.5, 5.8 Hz, 1H), 4.57 (s, 1H), 3.73 (s, 3H), 3.39 – 3.26 (m, 2H), 3.12 (dd, *J* = 17.7, 3.9 Hz, 1H), 2.71 (dd, *J* = 17.4, 6.0 Hz, 1H), 1.28 (s, 9H) ppm. ^{13}C NMR (151 MHz, Chloroform-*d*): δ = 172.50, 172.10, 171.67, 170.61, 165.20, 155.60, 139.49, 138.39, 136.36, 135.47, 132.83,

130.85, 129.24, 128.93, 128.67, 128.43, 128.29, 128.25, 127.22, 127.14, 126.66, 123.50, 120.70, 119.97, 117.96, 80.61, 66.95, 53.99, 52.52, 50.68, 37.90, 36.01, 28.21 ppm. HR-MS (ESI): calculated for $C_{47}H_{45}N_6O_7^+$[M+H]$^+$: m/z = 805.33442, found: 805.33364; calculated for $C_{47}H_{44}NaN_6O_7^+$[M+Na]$^+$: m/z = 827.31637, found: 827.31559; calculated for $C_{47}H_{44}KN_6O_7^+$[M+K]$^+$: m/z = 843.29031, found: 843.28943. IR (ATR): $\tilde{\nu}$ (cm^{-1}): 3311, 1738, 1689, 1653, 1589, 1525, 1500, 1483, 1374, 1346, 1298, 1170, 1020, 766, 726, 684, 645. Mp. 216 °C.

10.13 Preparation of *cyclo*-(Ala(3-(DPhCz(3-)T))-Asp(OBn)) (6) (Benzyl 2-((2*S*,5*S*)-5-((9-(4,6-diphenyl-1,3,5-triazin-2-yl)-9*H*-carbazol-3-yl)methyl)-3,6-dioxopiperazin-2-yl)acetate)

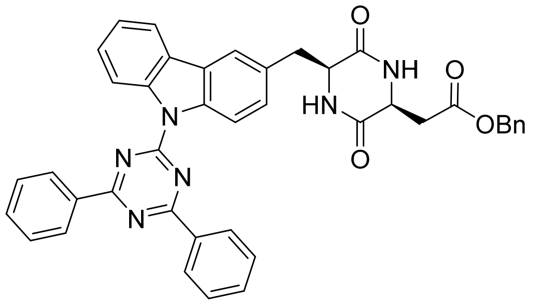

6

A slightly modified literature procedure was used.[9] Boc-Asp(OBn)-Ala(3-(DPhCz(3-)T))-OMe (**S11**, 940 mg, 1.15 mmol) was suspended in dry DCM (15 mL) and TFA (1.0 mL, 13.1 mmol) was added. The suspension quickly turned into a bright yellow solution that was stirred for 18 h at rt. Next, the solvent was removed and remaining TFA was co-evaporated with DCM, MeOH and toluene until a colorless solid was obtained. The solid was dried and rigorously ground to a fine powder. Next, the powder was suspended MeCN (15 mL) and 2-methylpropan-1-ol (10 mL) and NEt_3 was added (0.4 mL, 2.88 mmol). After stirring for 10 min at rt, AcOH (0.5 mL, 8.47 mmol) was added and the mixture was heated to 100 °C for 4 h. After cooling to rt, the suspension was filtered and washed first with acetone (75 mL), then with a hot mixture of acetone (45 mL) and DCM (5 mL). After drying, **6** was obtained as a colorless solid (633 mg, 932 µmol, 81%).

^{1}H NMR (600 MHz, Chloroform-*d*+TFA): δ = 8.98 (dd, J = 17.3, 8.5 Hz, 2H), 8.38 (d, J = 7.7 Hz, 4H), 8.23 (s, 1H), 8.13 (s, 1H), 8.01 (d, J = 7.6 Hz, 1H), 7.95 – 7.85 (m, 3H), 7.75 (t, J = 7.6 Hz, 4H), 7.65 (t, J = 7.9 Hz, 1H), 7.57 (t, J = 7.5 Hz, 1H), 7.43 (d, J =

8.6 Hz, 1H), 7.22 (d, *J* = 6.2 Hz, 3H), 7.09 (d, *J* = 6.9 Hz, 2H), 5.04 (d, *J* = 12.3 Hz, 1H), 4.75 (d, *J* = 12.2 Hz, 1H), 4.67 (t, *J* = 5.5 Hz, 1H), 4.45 (d, *J* = 8.9 Hz, 1H), 3.48 (ddd, *J* = 54.0, 14.4, 5.4 Hz, 2H), 2.74 (dd, *J* = 17.4, 3.5 Hz, 1H), 1.82 (dd, *J* = 17.4, 9.1 Hz, 1H) ppm. ^{13}C NMR (151 MHz, Chloroform-*d*+TFA): δ = 170.50, 169.29, 169.10, 167.15, 161.88, 138.84, 138.19, 136.55, 134.38, 132.57, 130.12, 129.86, 129.75, 129.05, 129.02, 128.87, 128.84, 128.76, 128.12, 127.84, 127.10, 121.80, 120.55, 120.40, 119.78, 68.02, 56.44, 51.44, 39.40, 37.89 ppm. HR-MS (ESI): calculated for $C_{41}H_{33}N_6O_4^+$[M+H]$^+$: m/z = 673.25578, found: 673.25530; calculated for $C_{41}H_{32}NaN_6O_4^+$[M+Na]$^+$: m/z = 695.23772, found: 695.23740; calculated for $C_{41}H_{32}KN_6O_4^+$[M+K]$^+$: m/z = 711.21166, found: 711.21126. IR (ATR): $\tilde{\nu}$ (cm^{-1}): 3058, 2889, 1737, 1675, 1589, 1526, 1501, 1485, 1455, 1372, 1328, 1265, 1175, 833, 765, 731, 702. Mp. 265 °C (decomp.).

Note: Without grinding, no full conversion to the DKP could be achieved due to the insolubility of the deprotected dipeptide.

10.14 Preparation of *cyclo*-(Ala(3-(DPhCz(3-)T))-Asp) (7) (2-((2*S*,5*S*)-5-((9-(4,6-diphenyl-1,3,5-triazin-2-yl)-9*H*-carbazol-3-yl)methyl)-3,6-dioxopiperazin-2-yl)acetic acid)

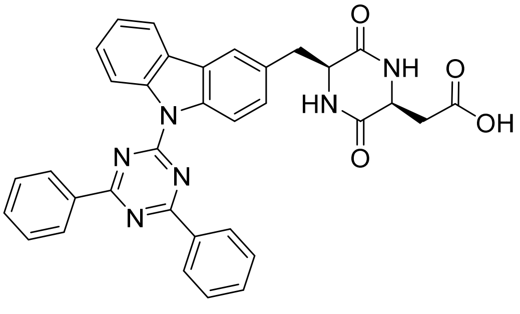

7

A slightly modified literature procedure was used.[11] *cyclo*-(Ala(3-(DPhCz(3-)T))-Asp(OBn) (**6**, 800 mg, 1.19 mmol) was placed in a dried Schlenk flask under a nitrogen atmosphere and suspended in dry DCM (11 mL). After cooling to -15 °C, BBr_3 (1 M in DCM, 4.76 mL, 4.76 mmoL) was added and the suspension was stirred for 20 h, slowly warming to rt. Then, after cooling to 0 °C, a mixture of water (1 mL) and *N*-methylmorpholine (1 mL) was added to quench the reaction and the mixture was stirred for 10 min at rt. MeOH (10 mL) and conc. H_3PO_4 (2 mL) were added to the suspension and it was stirred for additional 10 min at rt. It was filtered and washed with water

(100 mL), then suspended in water (50 mL) and sonicated for 5 min. After filtration, it was washed with more water (50 mL). It was then suspended in a mixture of acetone (20 mL) and MeOH (20 mL), sonicated for 5 min, filtered and washed with acetone (30 mL). After drying, **7** was obtained as an off-white solid (580 mg, 1.00 mmol, 84%).

^{1}H NMR (600 MHz, Chloroform-*d*+TFA): δ = 9.04 (dd, *J* = 13.1, 8.5 Hz, 2H), 8.38 (d, *J* = 7.8 Hz, 4H), 8.30 (d, *J* = 2.3 Hz, 1H), 8.19 (d, *J* = 2.4 Hz, 1H), 7.97 (d, *J* = 7.6 Hz, 1H), 7.94 – 7.87 (m, 3H), 7.74 (t, *J* = 7.7 Hz, 4H), 7.67 (t, *J* = 7.9 Hz, 1H), 7.57 (t, *J* = 7.5 Hz, 1H), 7.42 (dd, *J* = 8.6, 1.9 Hz, 1H), 4.72 (d, *J* = 5.2 Hz, 1H), 4.40 (d, *J* = 10.3 Hz, 1H), 3.59 (dd, *J* = 14.4, 5.3 Hz, 1H), 3.41 (dd, *J* = 14.4, 4.5 Hz, 1H), 2.69 (dd, *J* = 17.7, 3.4 Hz, 1H), 1.46 (dd, *J* = 17.6, 10.0 Hz, 1H) ppm. ^{13}C NMR (151 MHz, Chloroform-*d*+TFA): δ = 175.84, 169.31, 168.73, 167.07, 138.91, 138.29, 136.70, 132.34, 130.17, 129.93, 129.88, 129.82, 129.30, 128.79, 127.76, 127.23, 121.93, 120.57, 120.40, 119.93, 56.39, 51.06, 39.44, 37.58 ppm. HR-MS (ESI): calculated for $C_{34}H_{27}N_6O_4^+[M+H]^+$: m/z = 583.20883, found: 583.20880; calculated for $C_{34}H_{26}NaN_6O_4^+[M+Na]^+$: m/z = 605.19077, found: 605.19084. IR (ATR): $\tilde{\nu}$ (cm^{-1}): 3060, 1672, 1527, 1501, 1485, 1444, 1373, 834, 766, 705, 686, 644. Mp. 288 °C (decomp.).

10.15 Preparation of *cyclo*-(Ala(3-(DPhCz(3-)T))-Asp) *N*-methyl-D-glucamine salt (1) ((2*S*,3*R*,4*R*,5*R*)-2,3,4,5,6-pentahydroxy-*N*-methylhexyl-1-ammonium 2-((2*S*,5*S*)-5-((9-(4,6-diphenyl-1,3,5-triazin-2-yl)-9*H*-carbazol-3-yl)methyl)-3,6-dioxopiperazin-2-yl)acetate)

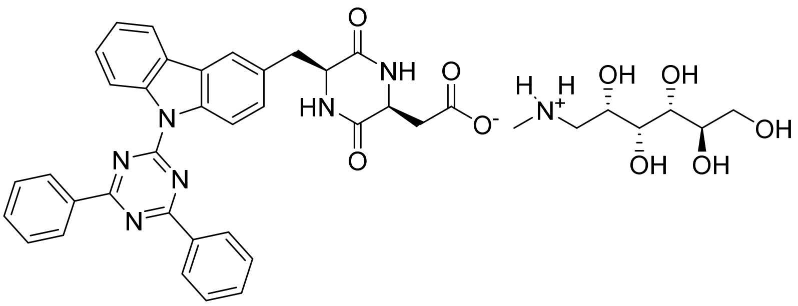

1

A slightly modified literature procedure was used.[12] *cyclo*-(Ala(3-(DPhCz(3-)T))-Asp) (**7**, 402 mg, 0.69 mmol) and *N*-methyl-D-glucamine (162 mg, 0,83 mmol) were suspended in abs. EtOH (20 mL) and heated to 50 °C for 3 h. The suspension was

filtered while still hot and washed with MeOH (50 mL) and DCM (50 mL). After drying, **1** was obtained as a colorless solid (456 mg, 0.59 mmol, 85%).

^{1}H NMR (600 MHz, D_2O/THF-d_8 1:1): δ = 8.96 (d, J = 7.1 Hz, 2H), 8.55 (d, J = 7.5 Hz, 4H), 8.15 (d, J = 7.4 Hz, 1H), 8.02 (s, 1H), 7.71 (t, J = 7.3 Hz, 2H), 7.60 (t, J = 7.4 Hz, 4H), 7.47 (dt, J = 22.4, 7.8 Hz, 3H), 4.51 – 4.32 (m, 2H), 4.18 (s, 1H), 3.93 – 3.86 (m, 2H), 3.82 (s, 0H), 3.73 (s, 2H), 3.46 (d, J = 14.4 Hz, 1H), 3.38 – 3.20 (m, 3H), 2.85 (s, 3H), 2.78 (d, J = 15.7 Hz, 1H), 2.23 (dd, J = 16.0, 10.1 Hz, 1H) ppm. ^{13}C NMR (151 MHz, D_2O/THF-d_8 1:1) δ = 178.23, 174.02, 171.27, 170.48, 166.72, 140.90, 139.72, 137.63, 135.08, 133.96, 130.76, 130.61, 128.92, 128.77, 128.49, 125.66, 122.91, 122.13, 119.76, 119.51, 73.09, 72.76, 72.63, 70.26, 64.91, 58.31, 54.86, 53.25, 43.21, 41.64, 35.05 ppm. HR-MS (ESI): calculated for $C_{34}H_{25}N_6O_4^-$[M-(meglumine)$^+$]$^-$: m/z = 581.19428, found: 581.19402; calculated for $C_{41}H_{42}N_7O_9^-$[M-H]$^-$: m/z = 776.30495, found: 776.30471. IR (ATR): $\tilde{\nu}$ (cm^{-1}): 3056, 2871, 1672, 1588, 1526, 1455, 1372, 1328, 1212, 1175, 1070, 1025, 834, 766, 705, 686, 644. Mp. 198 °C (decomp.).

11 Difficulties during and troubleshooting of the synthesis

This section is supposed to help and guide researchers who want to carry out similar syntheses.

In general, all lysine-derived compounds were incredibly insoluble in organic solvents. For aspartic acid-derived compounds, the DKPs were extremely insoluble, while only the protected dipeptide **S11** was readily soluble in DCM and $CHCl_3$.

Insolubility also led to small issues with the cyclization of the deprotected derivatives of **S10** and **S11** to DKPs **5** and **6** (Schemes S3, S4). The cyclization reactions only resulted in full conversion when the deprotected dipeptides were ground to a fine powder before the reaction.

Deprotection of Cbz-protected DKP **5** proved to be difficult at first, as it was simply too insoluble for hydrogenation reactions. Attempts to perform the hydrogenation using solvent mixtures, with a little amount of TFA for full solution or with increased hydrogen pressure all failed. From our group's experience, many Cbz-protected, lysine-derived DKPs tend to be extremely insoluble. The HBr-mediated deprotection[10] might therefore be a practical alternative to hydrogenation reactions for insoluble DKPs with CBz protected side chains.

The deprotection of benzyl ester containing DKP **6** also proved to be difficult, as palladium-catalyzed hydrogenation attempts failed due to the same reasons as for **5**. Saponification attempts also failed, either due to insolubility or gelation very early on during the reaction, giving subsequent purification problems. For the BBr_3 mediated deprotection,[11] a long reaction time and an excess of BBr_3 were necessary because **6** was almost completely insoluble in DCM. We found that it was paramount to use relatively fresh BBr_3 (<1 year after opening) for the deprotection, as racemization presumably at the aspartic acid side chain of about 10% - 20% took place with older charges.

Attempts to synthesize **2** from the doubly Boc-protected derivative of **S10**, similar to the literature,[13] failed and led only to decomposition during the cyclization reaction. We also tried to synthesize the glutamic acid derivative of **6**, but cyclization of the deprotected dipeptide precursor failed to yield the desired product. In this case, cyclization happened not as usual on the α-carboxylic acid ester, but selectively on the *δ*-carbonyl of the side chain ester, which led to more side reactions.

12 References

[1] R. G. Vaswani, A. R. Chamberlin, *J. Org. Chem.* **2008**, *73*, 1661–1681.

[2] M. Brandstätter, F. Roth, N. W. Luedtke, *J. Org. Chem.* **2015**, *80*, 40–51.

[3] B. M. Trost, M. T. Rudd, *Org. Lett.* **2003**, *5*, 4599–4602.

[4] S. M. Bonesi, R. Erra-Balsells, *J. Heterocycl. Chem.* **2001**, *38*, 77–87.

[5] Z.-F. An, R.-F. Chen, J. Yin, G.-H. Xie, H.-F. Shi, T. Tsuboi, W. Huang, *Chem. Eur. J.* **2011**, *17*, 10871–10878.

[6] M. Hu, Y. Liu, Y. Chen, W. Song, L. Gao, H. Mu, J. Huang, J. Su, *RSC Adv.* **2017**, *7*, 7287–7292.

[7] A. J. Ross, H. L. Lang, R. F. W. Jackson, *J. Org. Chem.* **2010**, *75*, 245–248.

[8] E. Erdik, *Tetrahedron* **1987**, *43*, 2203–2212.

[9] K. Suzuki, Y. Sasaki, N. Endo, Y. Mihara, *Chem. Pharm. Bull.* **1981**, *29*, 233–237.

[10] D. Ben-Ishai, A. Berger, *J. Org. Chem.* **1952**, *17*, 1564–1570.

[11] P. D. Leeson, J. C. Emmett, *J. Chem. Soc., Perkin Trans. 1* **1988**, 3085–3096.

[12] M. Ohno, Y. Tanaka, M. Miyamoto, T. Takeda, K. Hoshi, N. Yamada, A. Ohtake, *Bioorg. Med. Chem.* **2006**, *14*, 2005–2021.

[13] 13a) C. Balachandra, T. Govindaraju, *J. Org. Chem.* **2020**, *85*, 1525–1536; 13b) Z. L. Pianowski, J. Karcher, K. Schneider, *Chem. Commun.* **2016**, *52*, 3143–3146; 13c) J. Karcher, Z. L. Pianowski, *Chem. Eur. J.* **2018**, *24*, 11605–11610.

13 NMR spectra

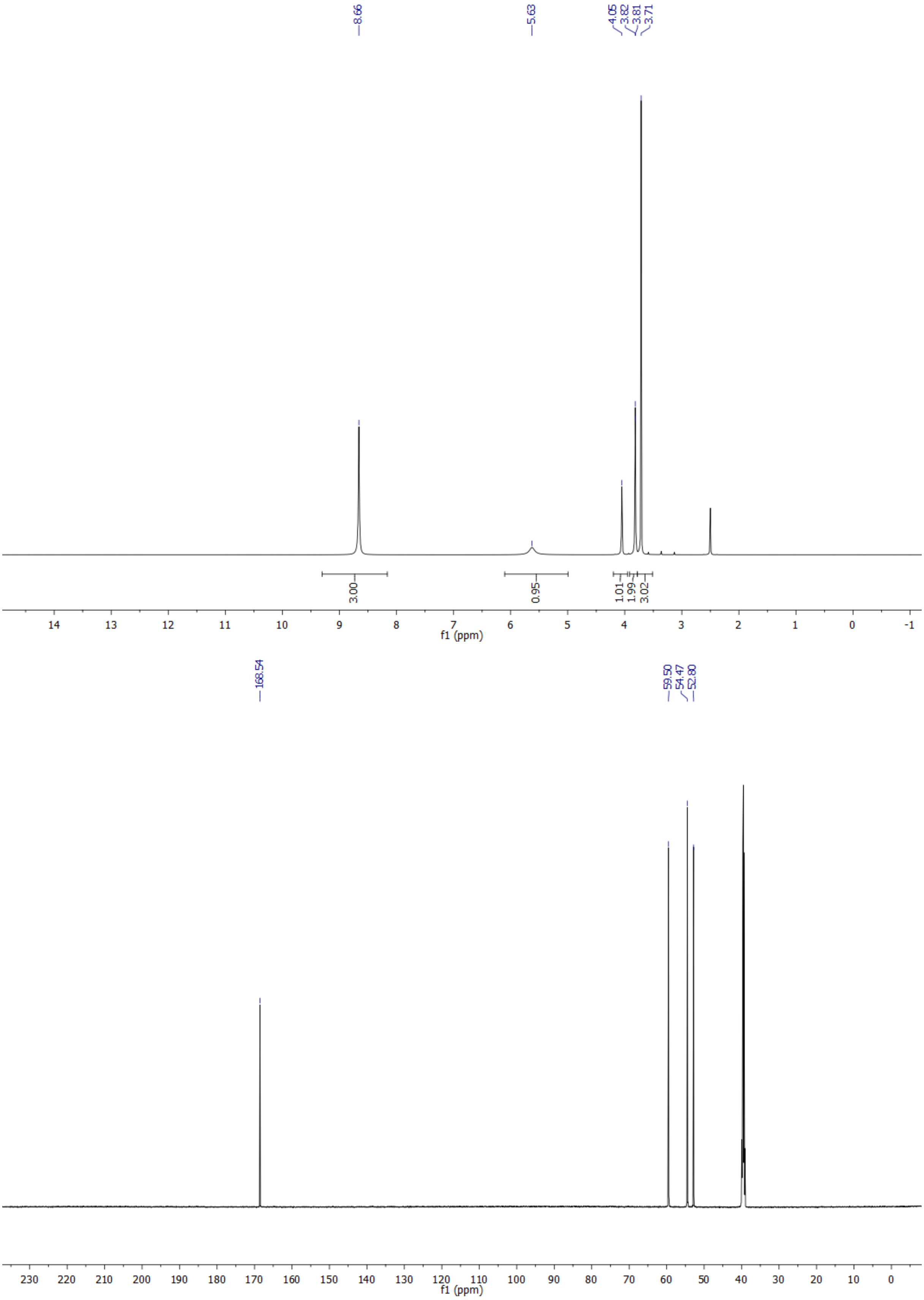

Figure S85. ^{1}H- and ^{13}C-NMR spectra of **S2** in DMSO-d_6.

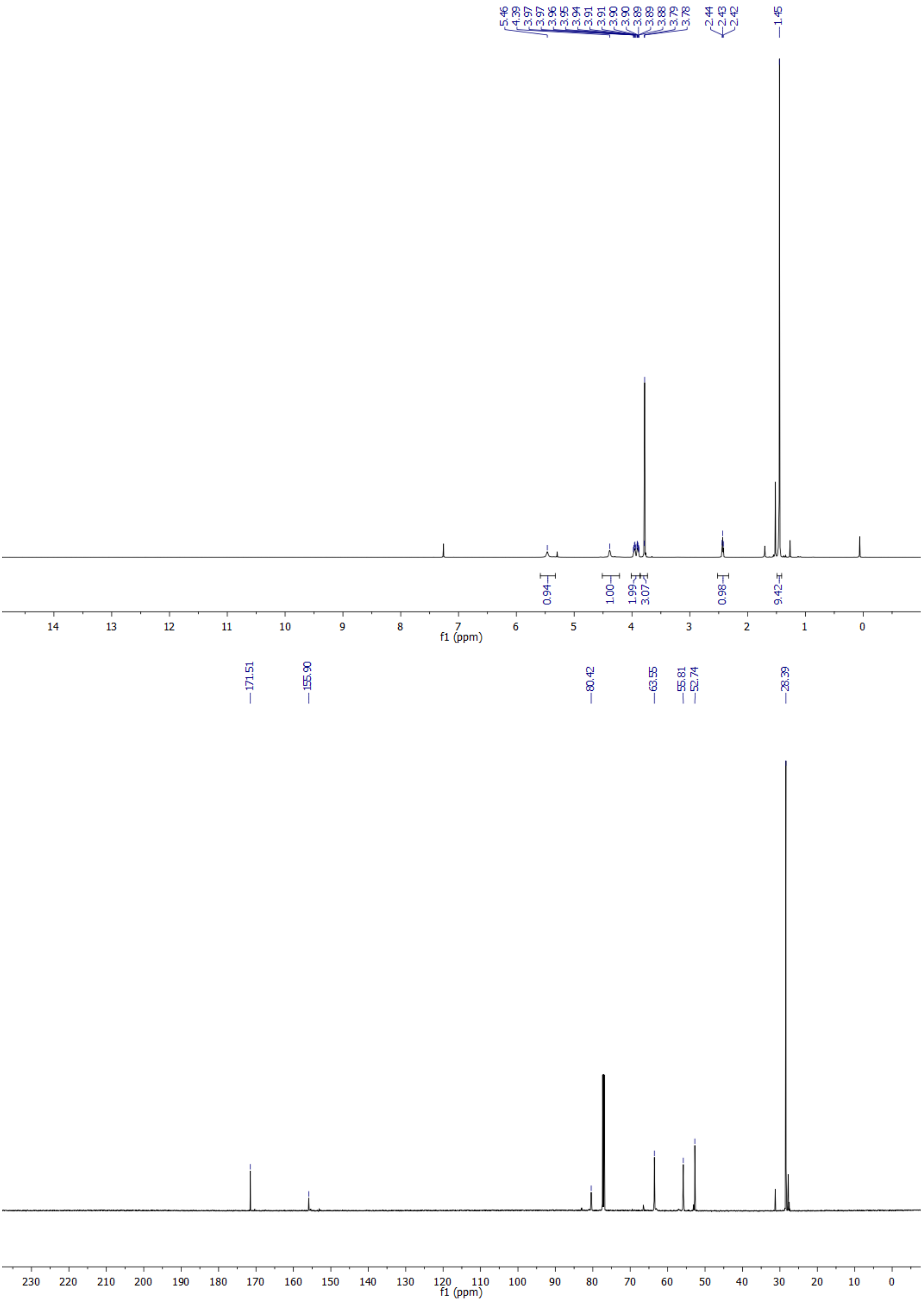

Figure S86. ^{1}H- and ^{13}C-NMR spectra of **S3** in $CDCl_3$.

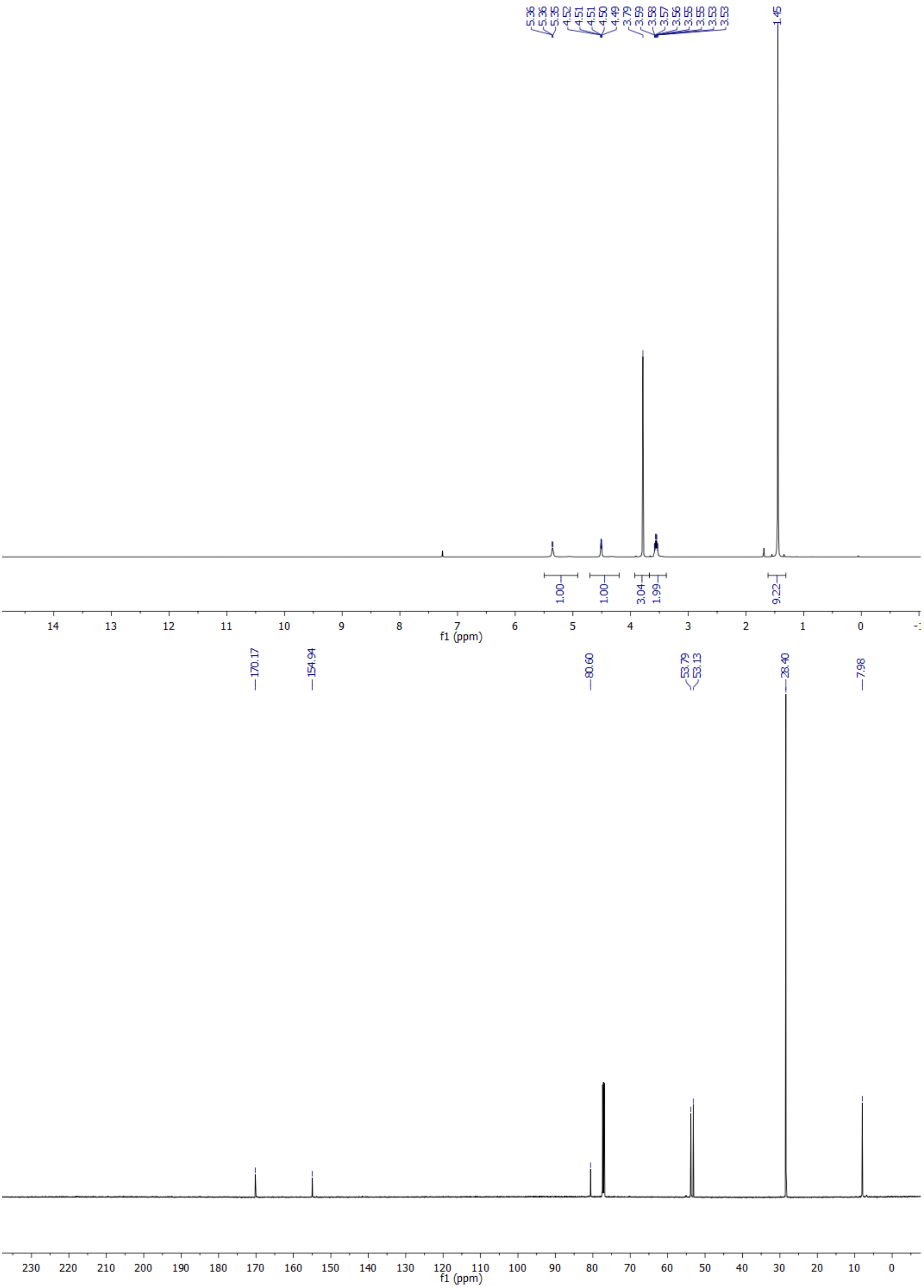

Figure S87. ^{1}H- and ^{13}C-NMR spectra of **S4** in $CDCl_3$.

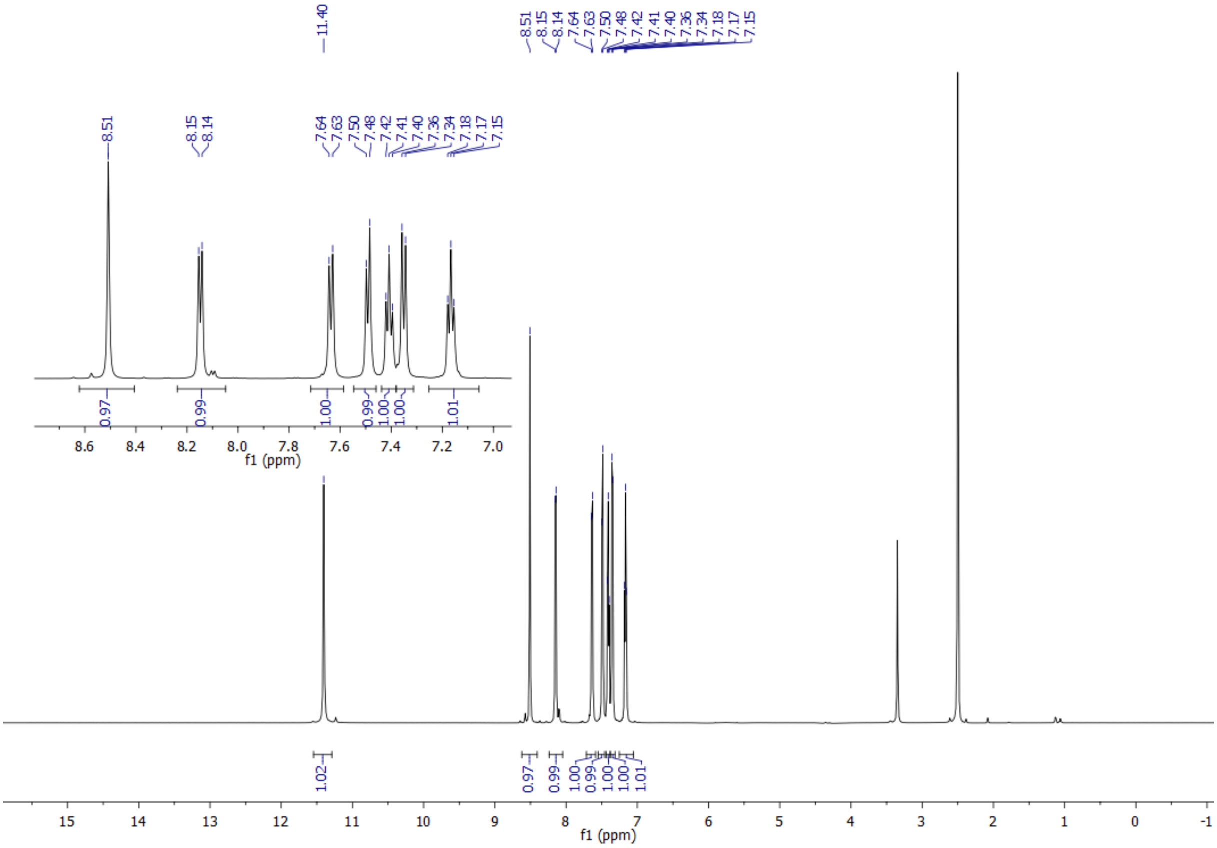

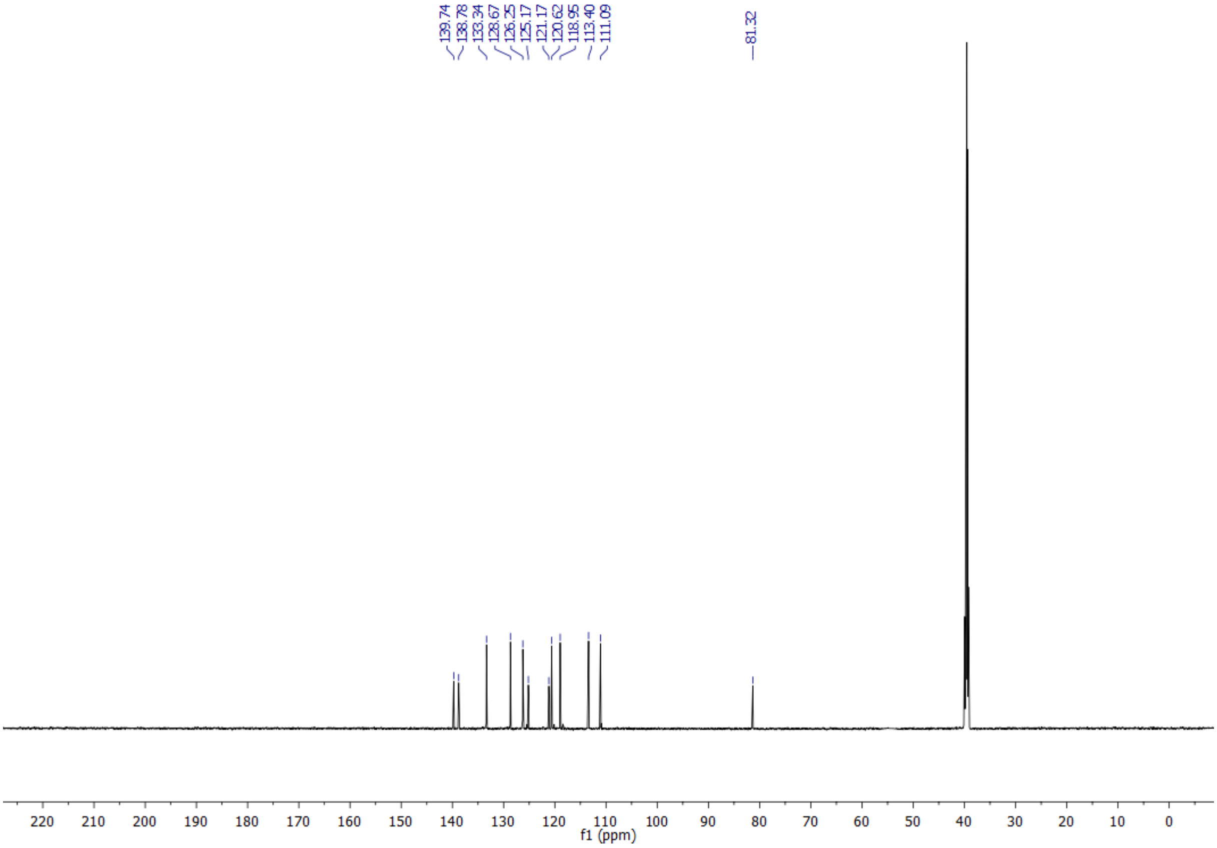

Figure S88. ^{1}H- and ^{13}C-NMR spectra of **S6** in DMSO-d_6.

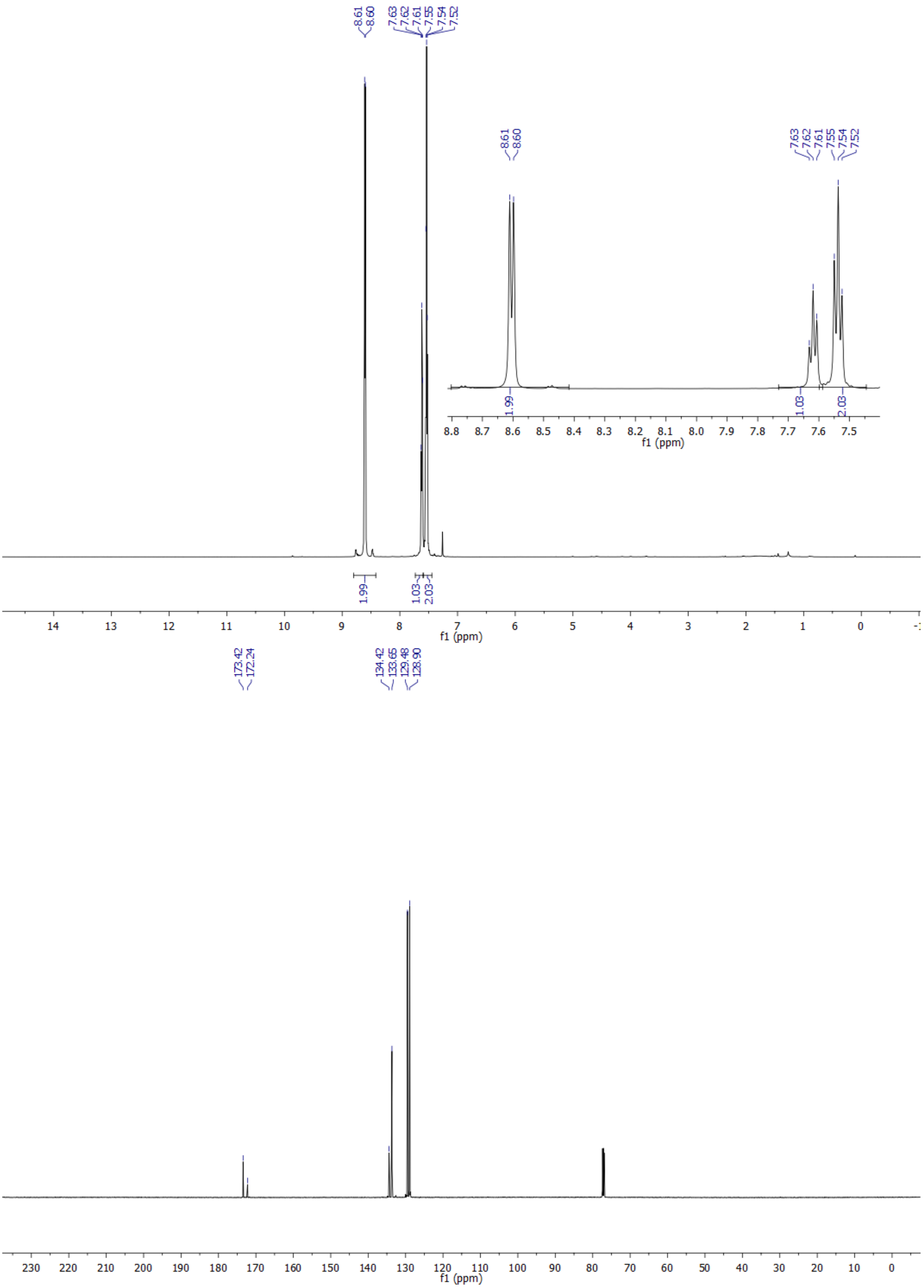

Figure S89. ^{1}H- and ^{13}C-NMR spectra of **S8** in $CDCl_3$.

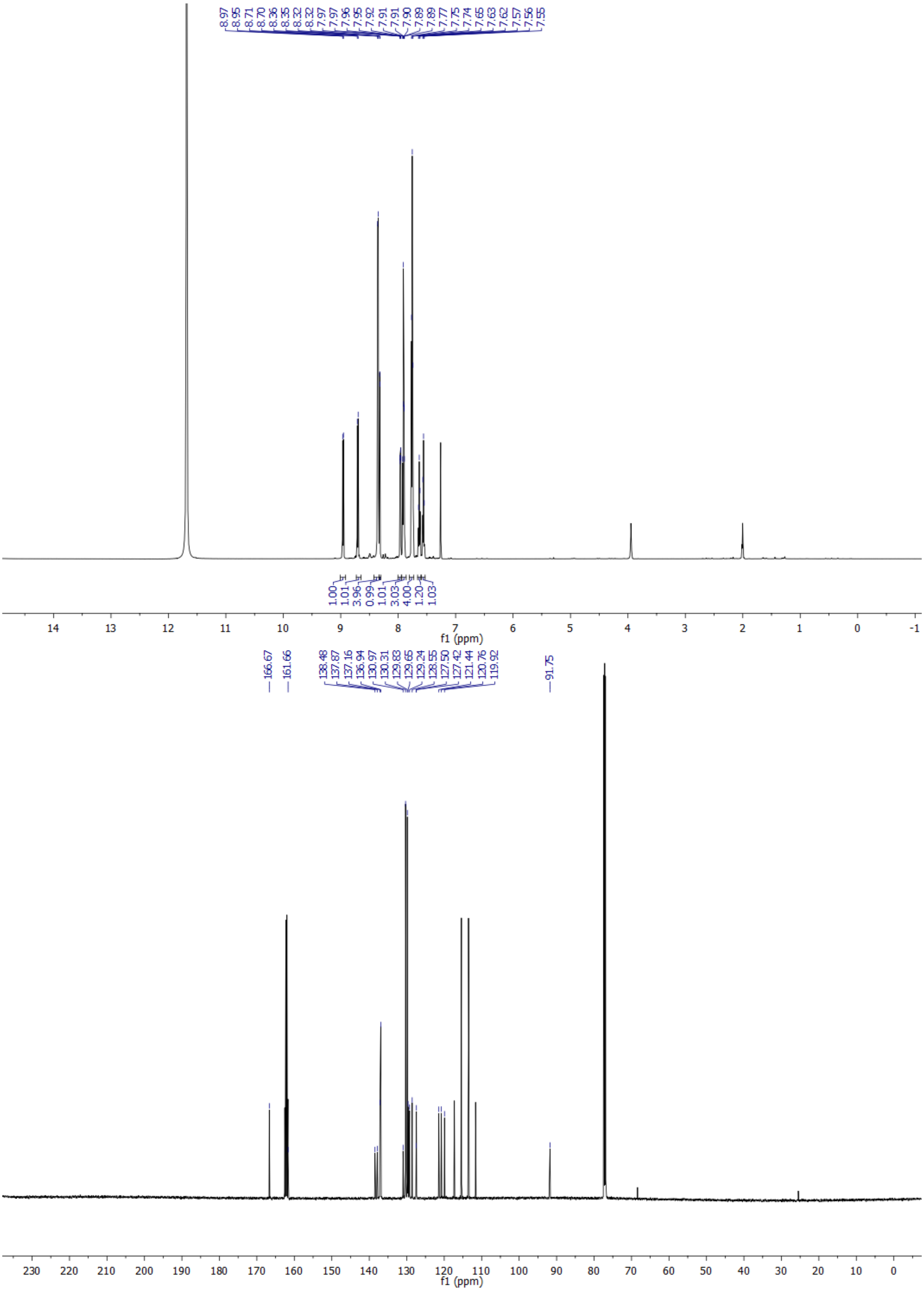

Figure S90. ^{1}H- and ^{13}C-NMR spectra of **3** in $CDCl_3$+TFA.

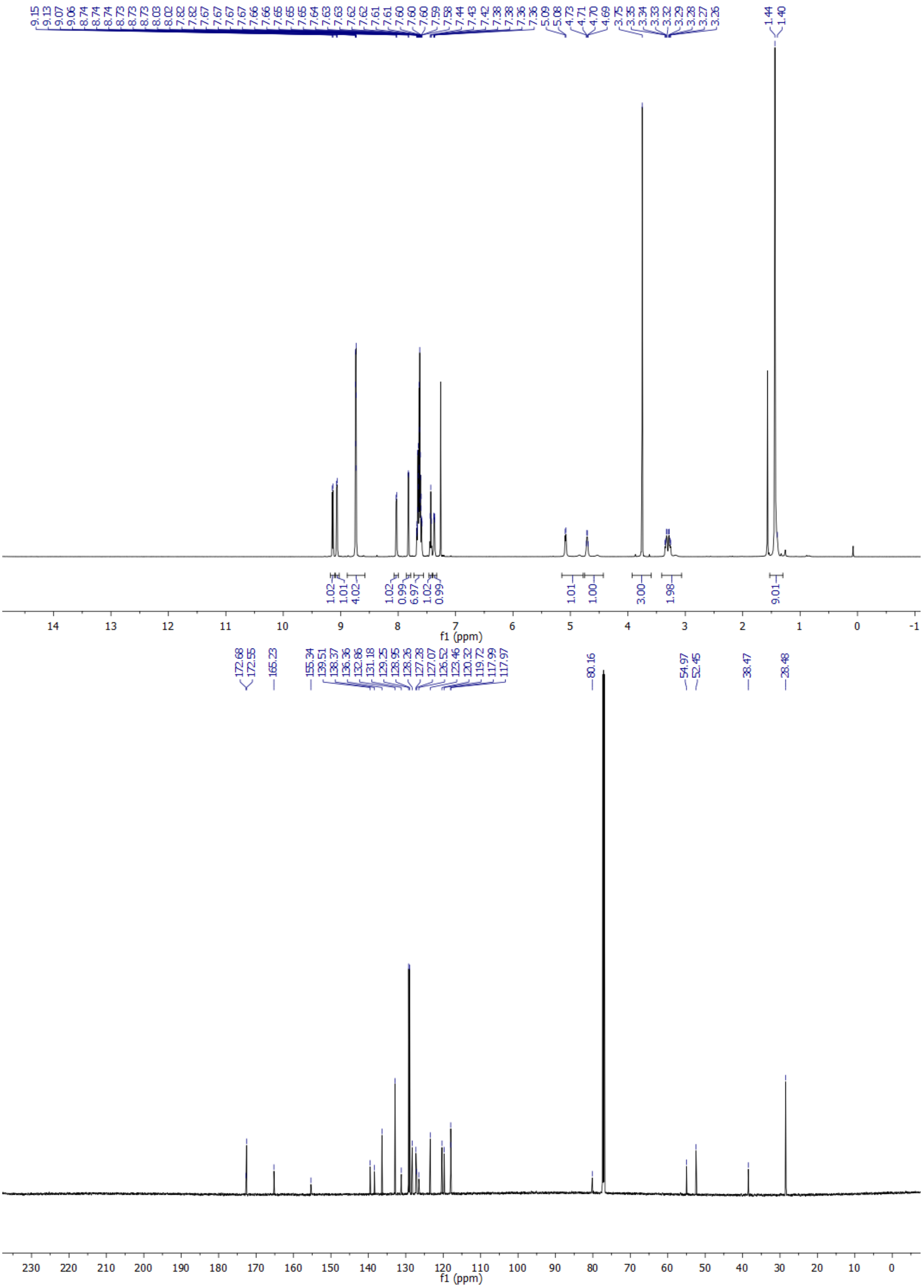

Figure S91: ^{1}H- and ^{13}C-NMR spectra of **4** in $CDCl_3$.

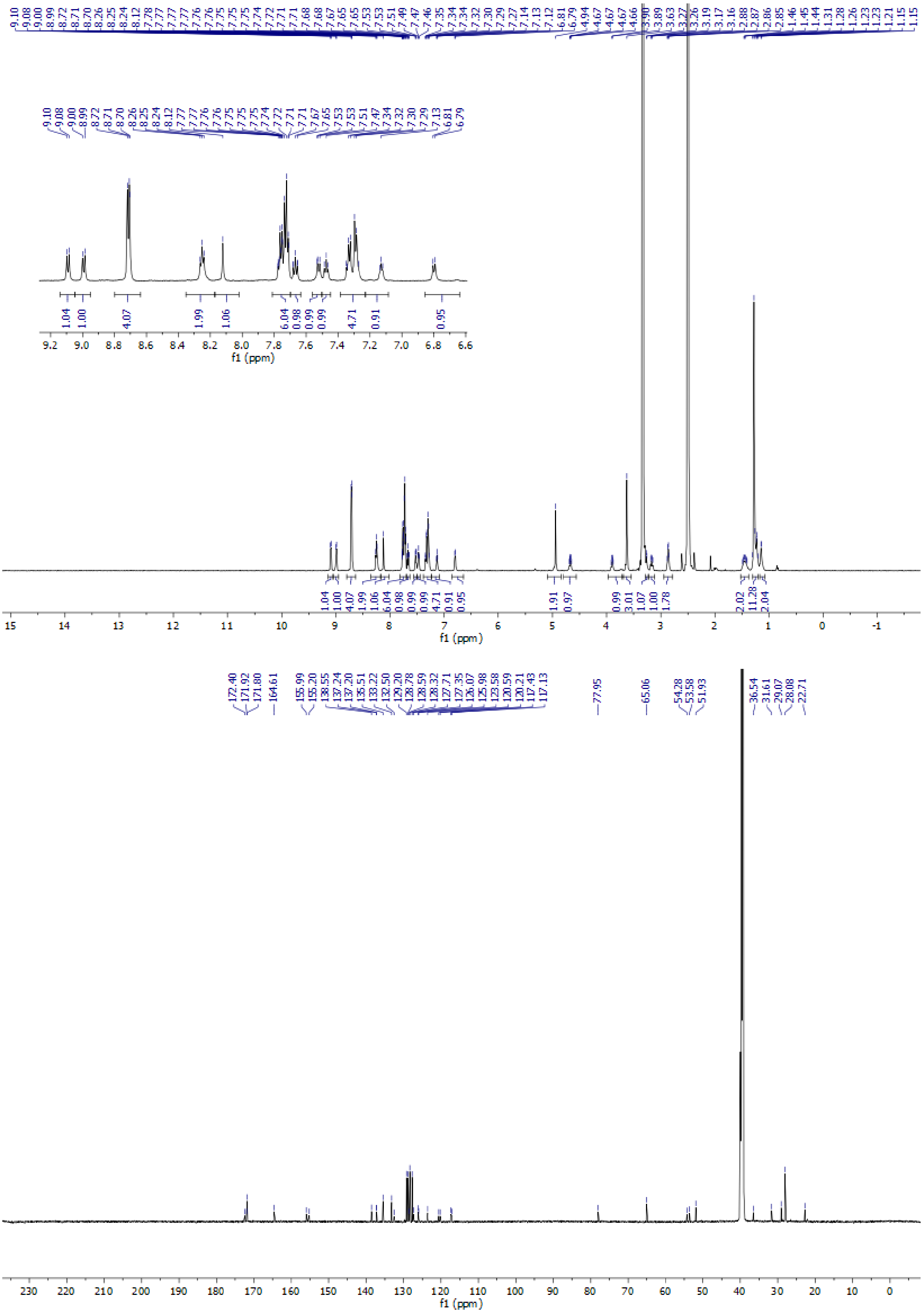

Figure S92. ^{1}H- and ^{13}C-NMR spectra of **S10** in DMSO-d_6.

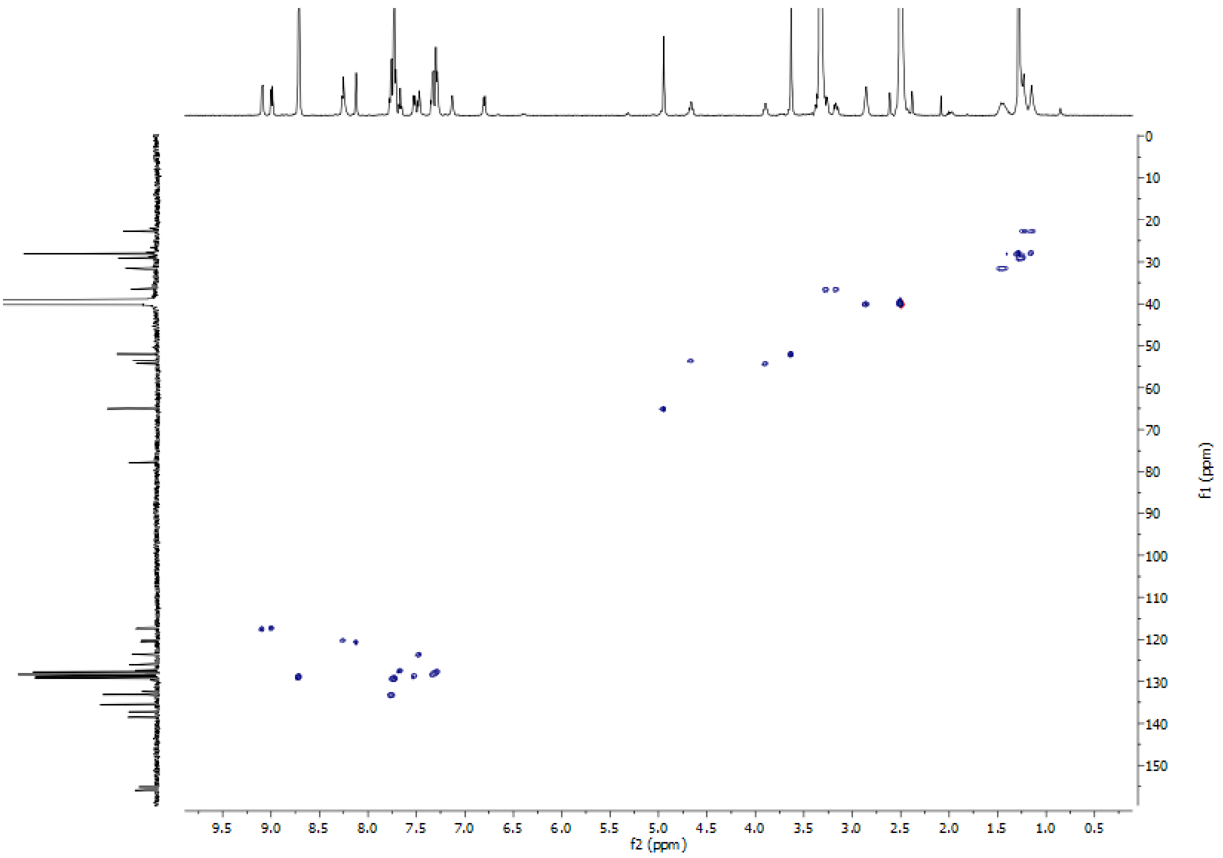

Figure S93. ^{1}H -^{13}C-HSQC spectrum of **S10** in DMSO-d_6.

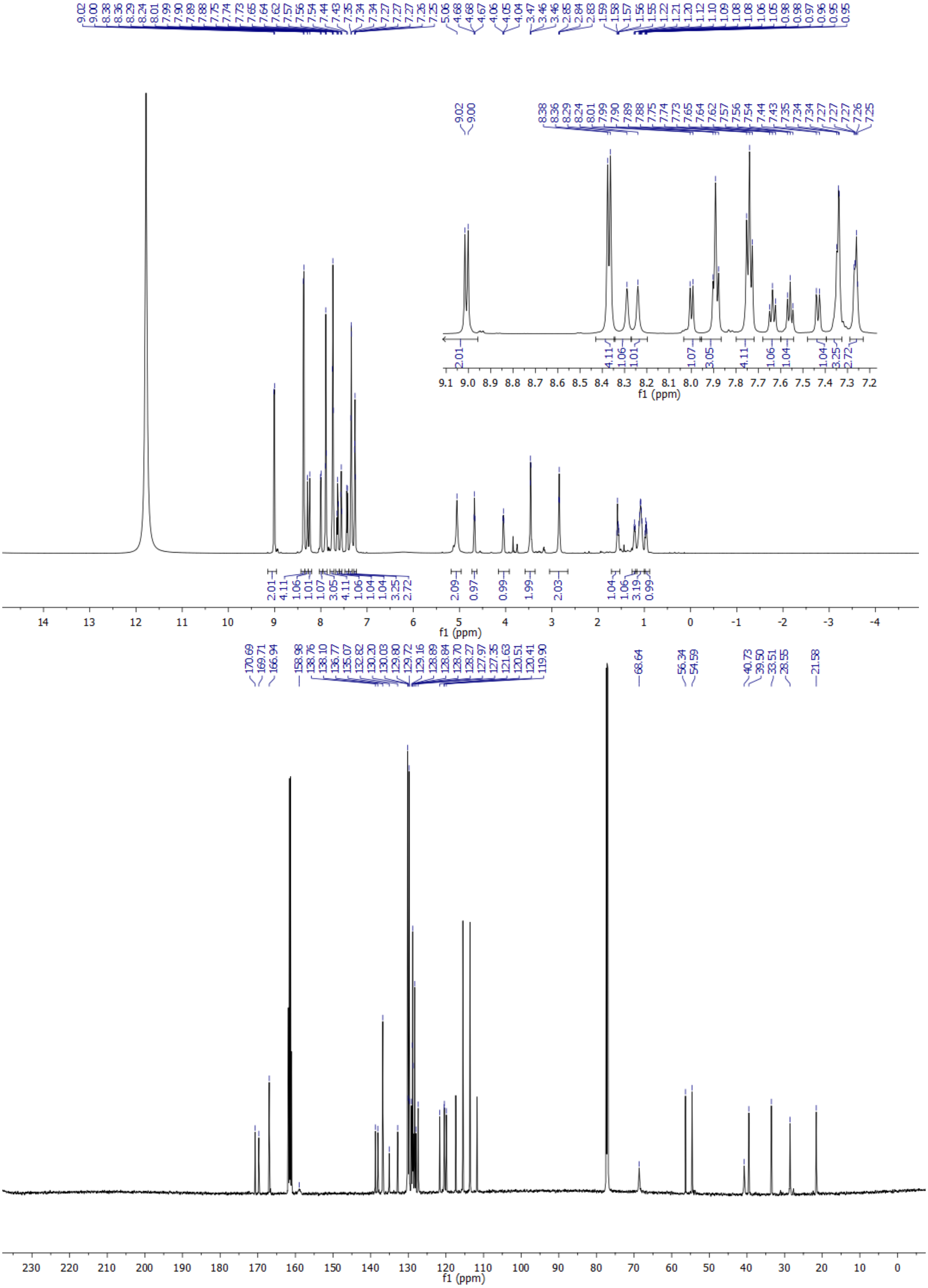

Figure S94. ^{1}H- and ^{13}C-NMR spectra of **5** in $CDCl_3$+TFA.

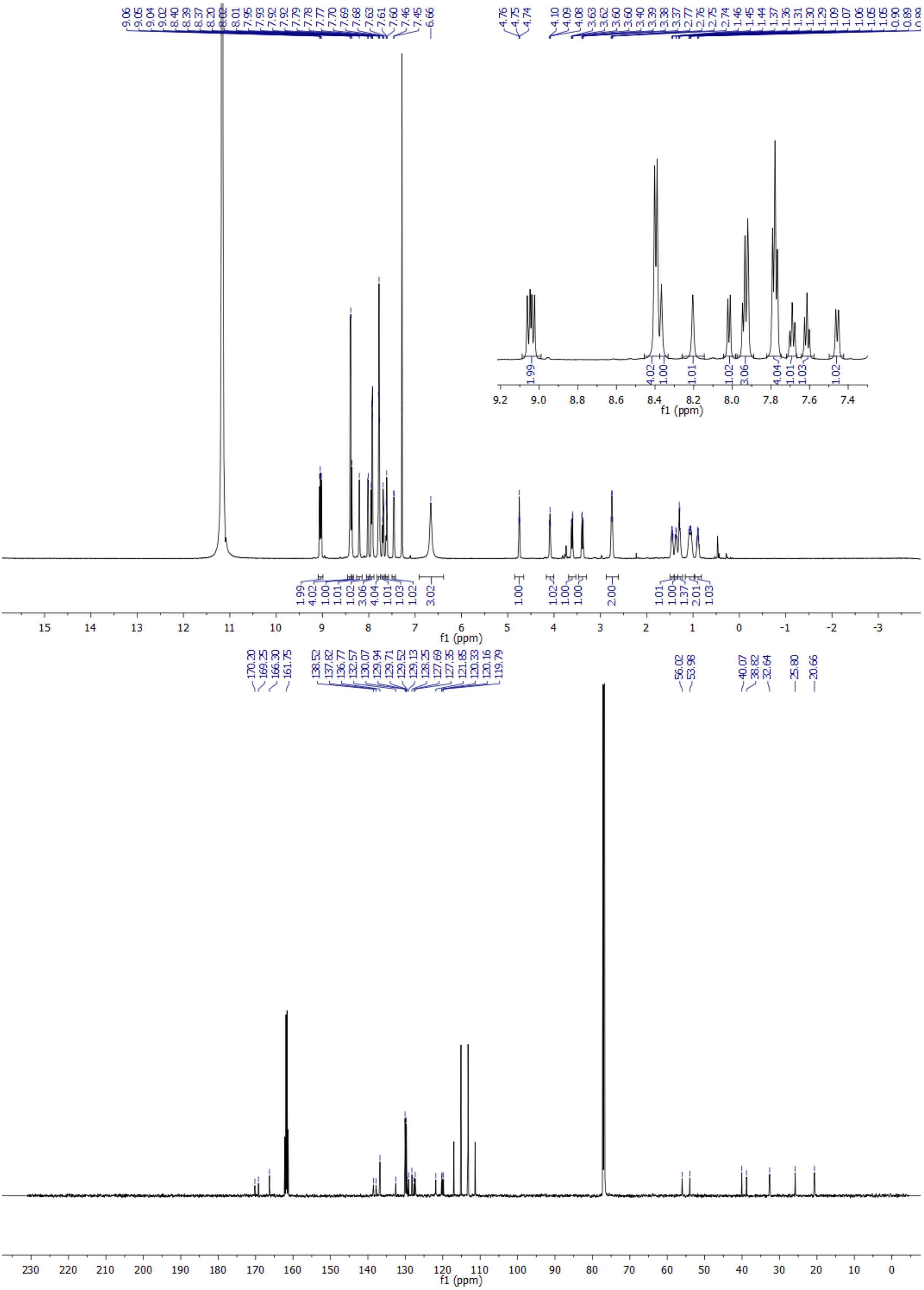

Figure S95: ^{1}H- and ^{13}C-NMR spectra of **2** in $CDCl_3$+TFA.

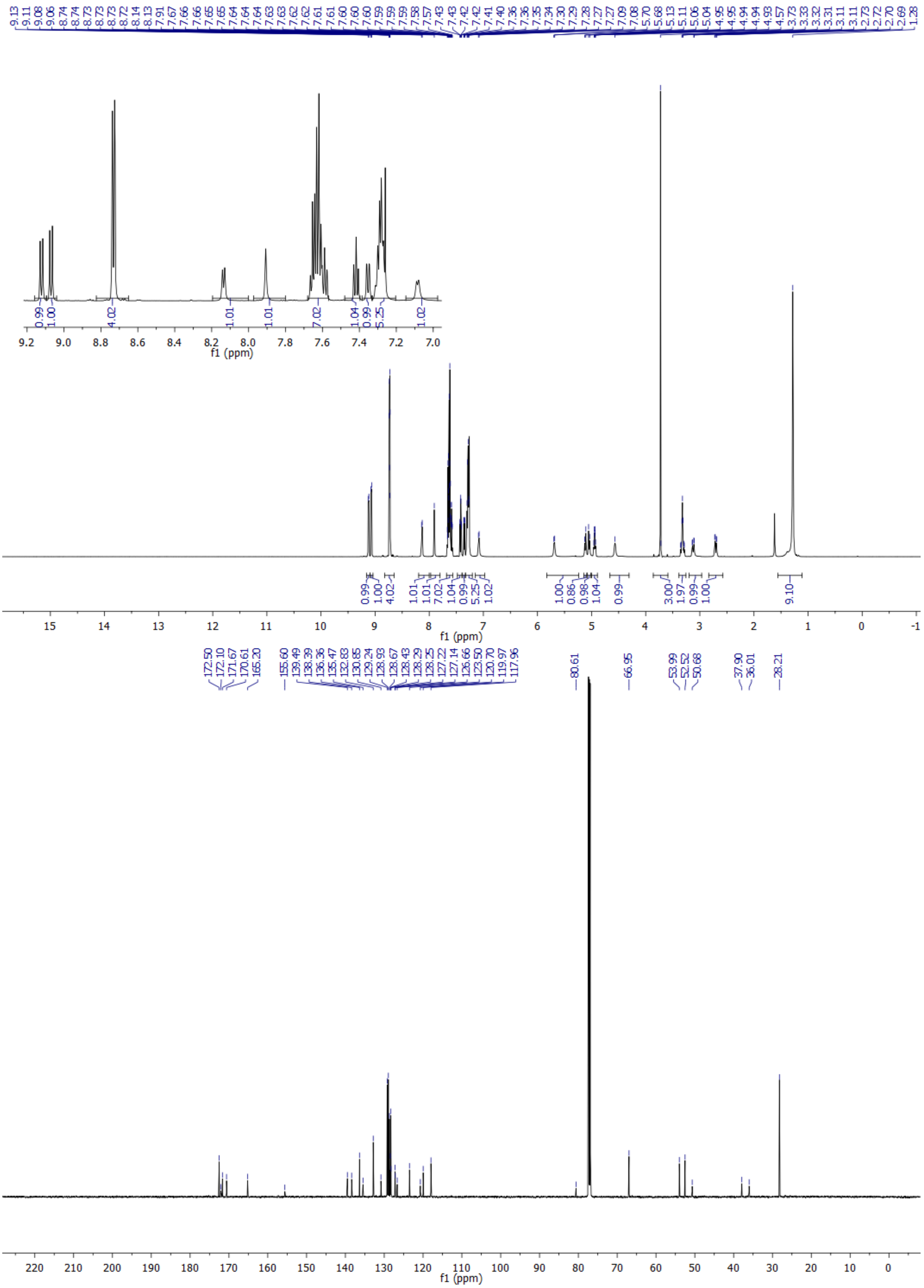

Figure S96. ^{1}H- and ^{13}C-NMR spectra of **S11** in $CDCl_3$.

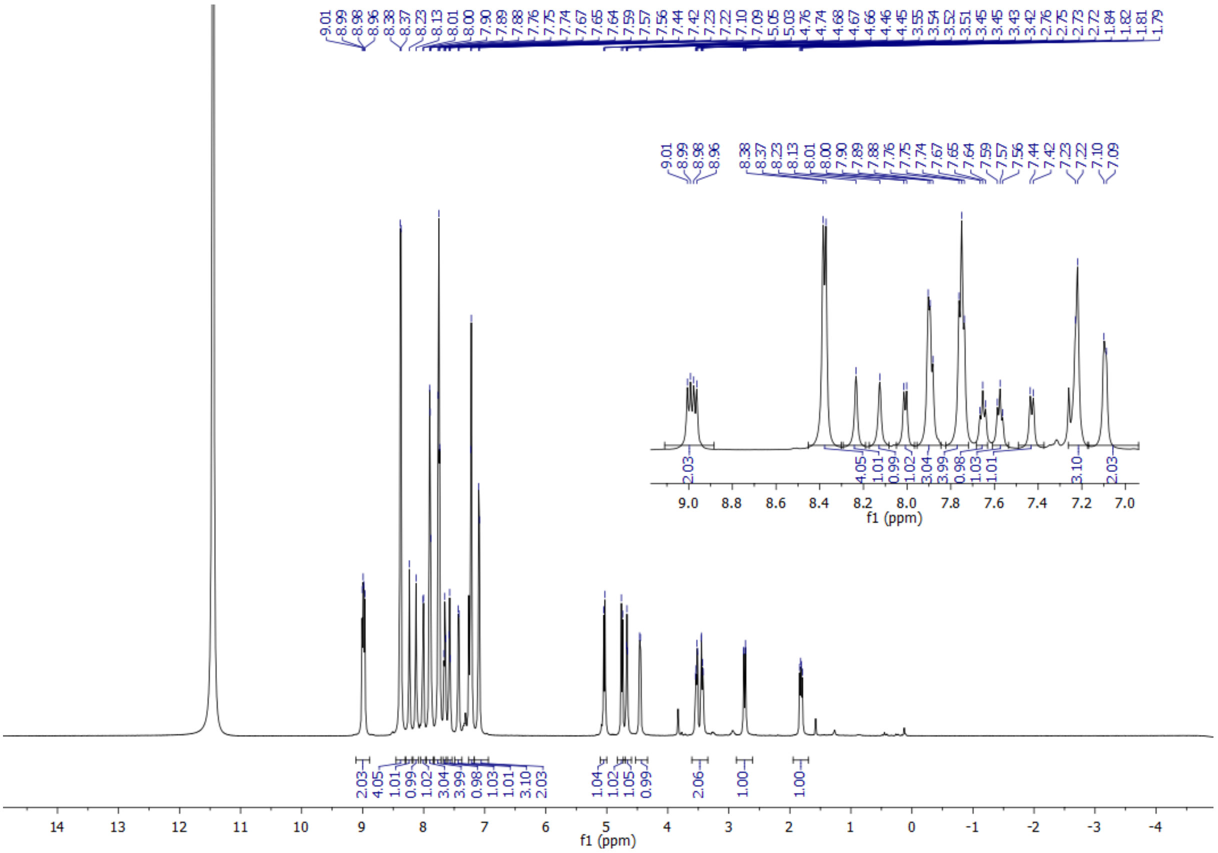

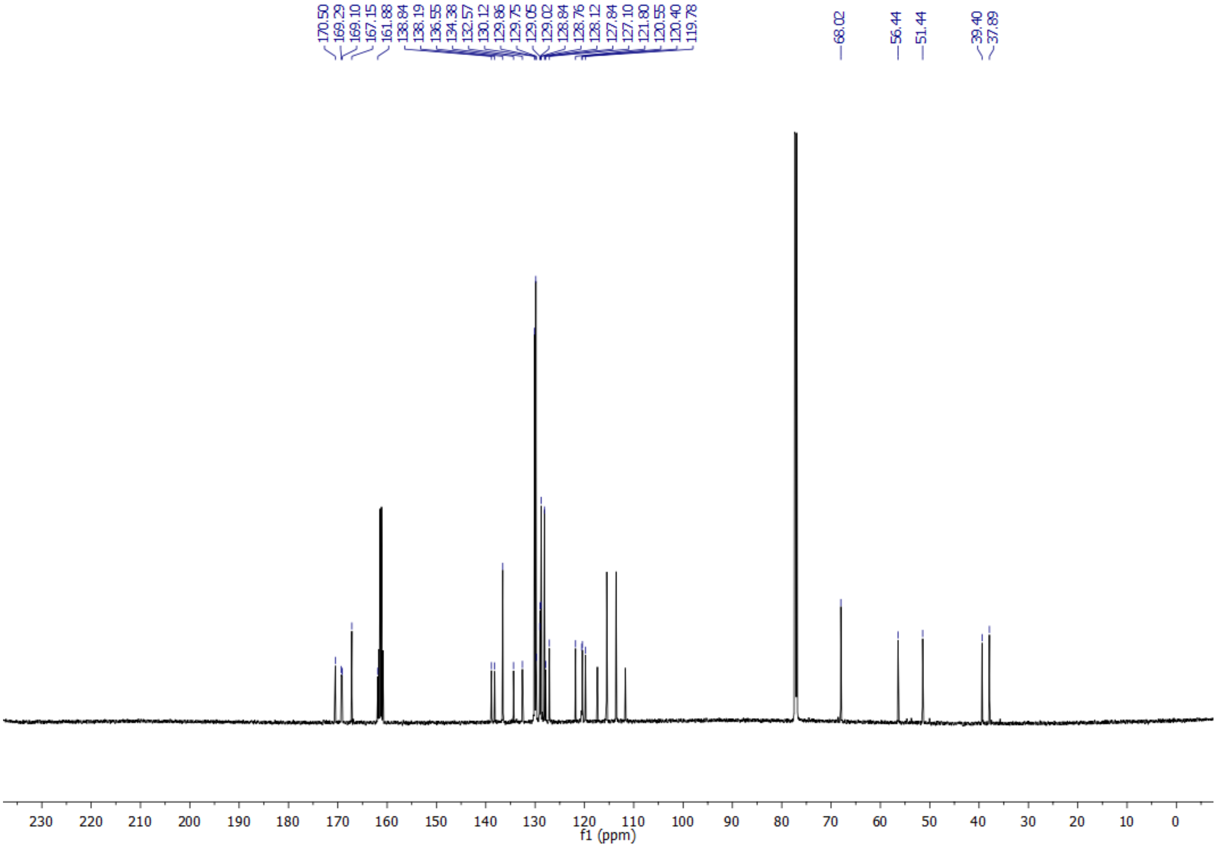

Figure S97. ^{1}H- and ^{13}C-NMR spectra of **6** in $CDCl_3$+TFA.

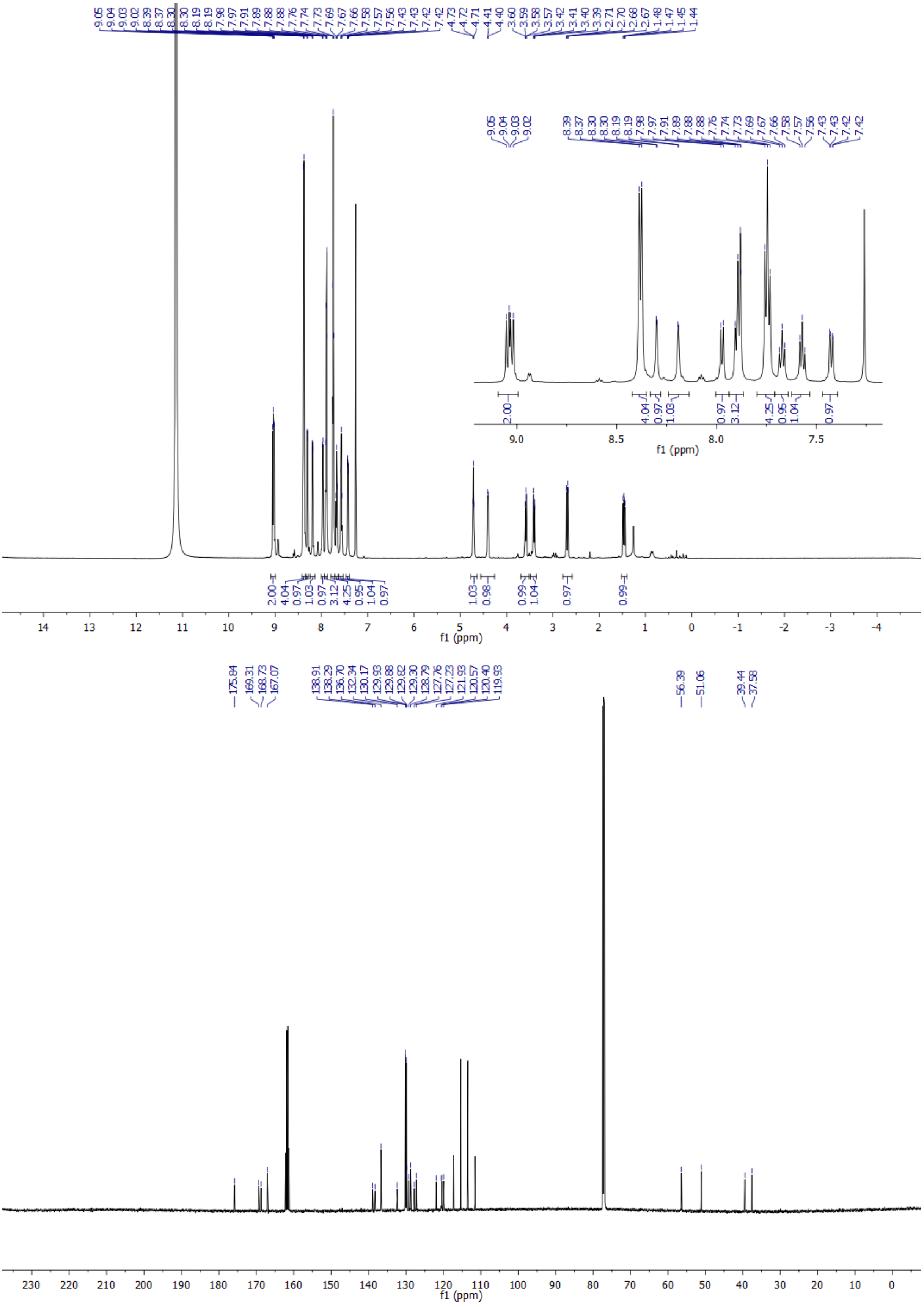

Figure S98. ^{1}H- and ^{13}C-NMR spectra of **7** in $CDCl_3$+TFA.

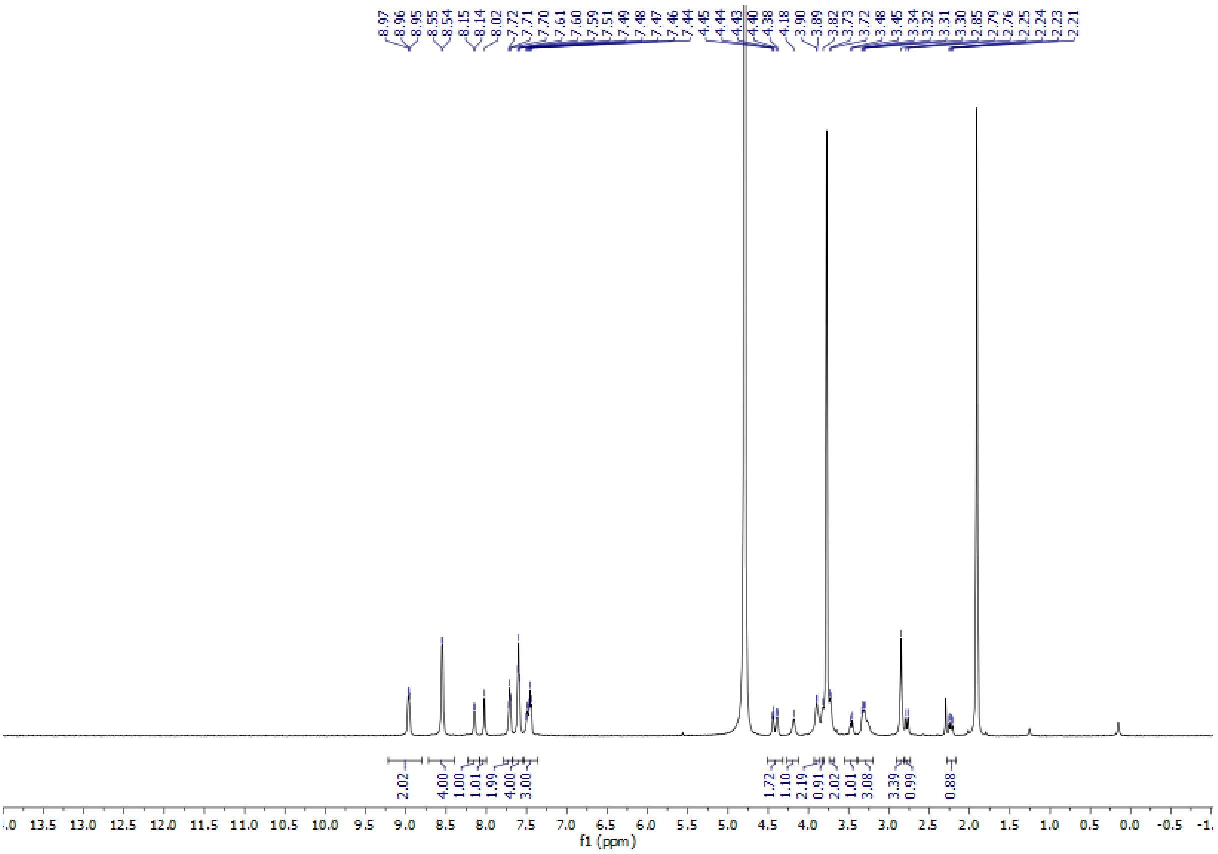

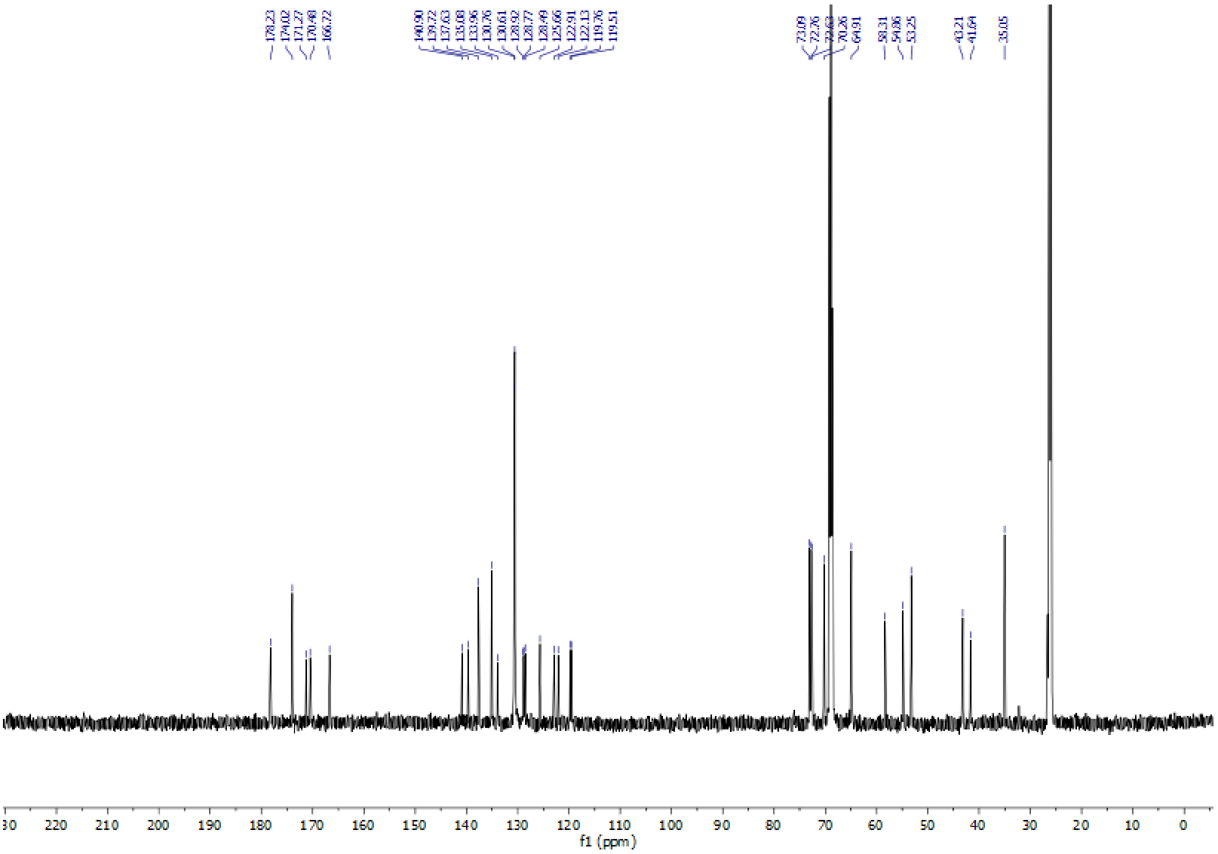

Figure S99. ^1H- and ^{13}C-NMR spectra of **1** in D_2O/THF-d_8.

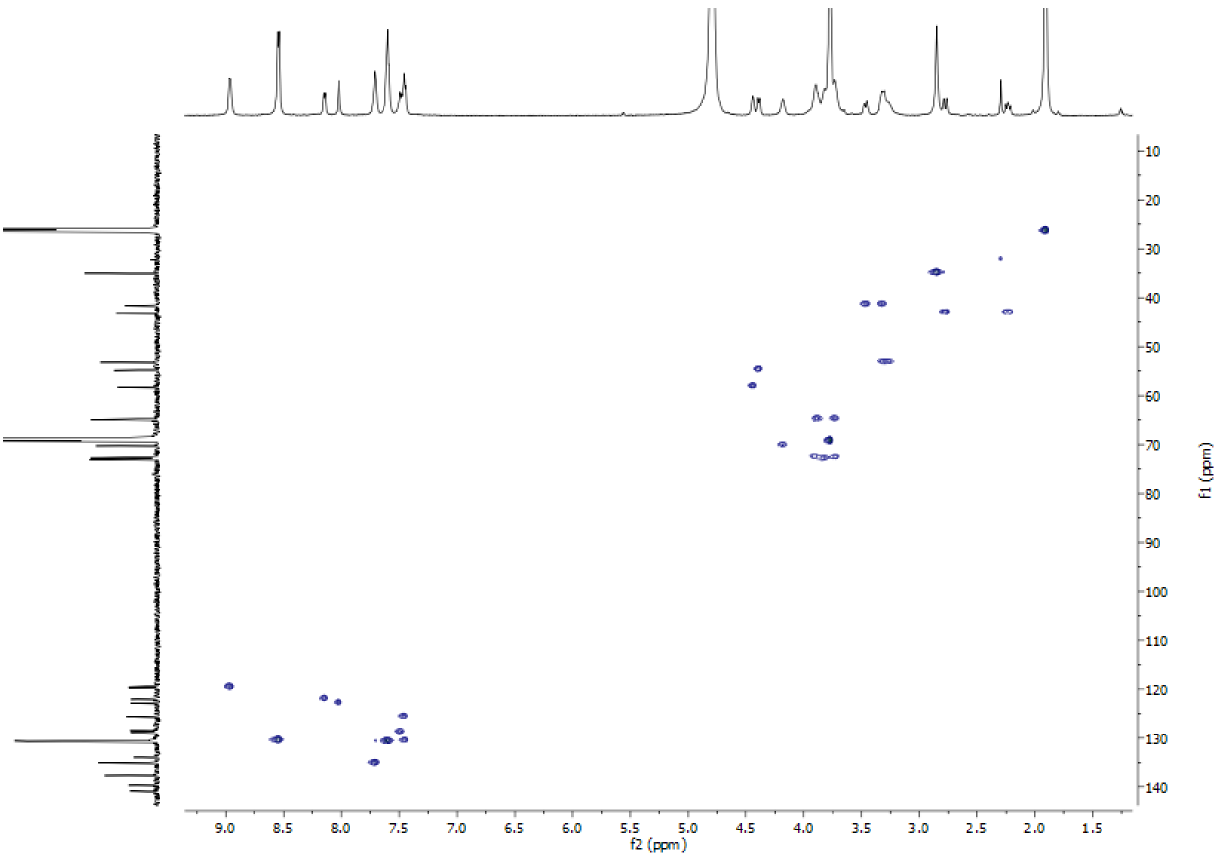

Figure S100. ^{1}H -^{13}C-HSQC spectrum of **1** in D_2O/THF-d_8.
